# Improving the design of epidemiology studies that use biomonitoring for exposure assessment: a SciPinion panel recommendation

**DOI:** 10.1186/s12874-025-02753-5

**Published:** 2026-01-12

**Authors:** Igor Burstyn, Louis Anthony Cox, Yang Cao, Guy Eslick, Shelley Harris, Leeka Kheifets, Michael Kramer, Peter Langlois, Paul Lee, Boris Reiss, Trudy Voortman, Tyler M. Carneal, Sean M. Hays

**Affiliations:** 1 Drexel University, Philadelphia, PA USA; 2 University of Colorado, Denver, CO USA; 3 Örebro University, Örebro, Sweden; 4 The University of Newcastle, Callaghan, Australia; 5 University of Toronto, Toronto, ON Canada; 6 University of California Los Angeles, Los Angeles, CA USA; 7 McGill University, Montreal, QC Canada; 8 University of Texas School of Public Health, Dallas, TX USA; 9 University of Southampton, Southampton, England; 10 KU Leuven, Leuven, Belgium; 11 Stanford University, Stanford, CA USA; 12 SciPinion, Bozeman, MT USA

**Keywords:** Epidemiology, Biomonitoring, Power, Bias, Software, Measurement error

## Abstract

**Background:**

Epidemiological studies that rely on biomarkers of exposure typically estimate each subject’s exposure from measurements on that individual. If repeated measurements of biomarkers of exposure are obtained on an individual, they are typically averaged. This averaging helps to reduce error from within-person variability if average exposure is a better measure of the biologically effective dose than the instantaneous one. However, these analyses then often ignore the residual within-person variation in the averages of measurements. Not considering this variation can bias effect estimates and lead to inaccurate risk assessment.

**Methods:**

We developed software (“calculators”) that help design studies of continuous and binary outcomes that rely on biomarkers of exposure. An independent panel of experts was employed to peer review the models and answer questions regarding their use and best practices for the design of epidemiology studies that utilize biomonitoring data for the exposure assessment.

**Results:**

Web-based tools were developed to estimate the required sample sizes, number of repeated measurements, and the trade-offs between power and bias in simple linear and logistic regression models under classical (independent, additive, normally distributed, homogeneous variance) measurement error assumptions. Application of the calculators was illustrated in case studies of investigation of the associations between urinary levels of bisphenols during pregnancy and fetal growth, and urinary levels of triclosan and neurodevelopment in children. Best practices are recommended for the design of epidemiology studies that utilize biomonitoring data for the exposure assessment.

**Conclusions:**

Calculators have been developed and vetted by a panel of experts. They are designed to estimate sample size (number of individuals sampled and number of samples per individual), power and bias in epidemiological studies that use biomonitoring to assess each subject’s exposure in the presence of classical measurement errors. These user-friendly tools account for measurement error and allow researchers to design more accurate and appropriately powered studies, ultimately improving quality of public health research.

**Supplementary Information:**

The online version contains supplementary material available at 10.1186/s12874-025-02753-5.

## Background

Epidemiological studies seek to estimate associations between exposures and health outcomes. Some epidemiologic studies compared risk and exposures on an aggregate basis for populations of many individuals (e.g. comparing health outcomes across communities or occupations with different exposures). The appeal of individual-based exposure and outcome evaluation lies in the promise of more accurate measurement of exposure and outcomes. It also controls -- via study design or data analysis -- for differences among individuals that can distort exposure-outcome relationships. Biomarkers of exposure can be unbiased measure of the biologically effective dose, but it is important to consider whether there is inter-individual variation in this relationship. Biomarkers of exposure, and exposure measurements in general, hold considerable promise for reducing errors in exposure estimates, especially when collected on all persons enrolled in a study. However, individual-based exposure estimates are often measured imprecisely, owing to temporal variations in exposure and biomarker levels [1], and, to a lesser extent, analytical errors (as is commonly assumed). The resulting biases can be large enough to change study conclusions, especially when variability in exposure and biomarkers within persons make it difficult to distinguish persons with different exposure levels, i.e. if between-person variability is not much larger than within-person variability. This was recognized as early as the 1990 s with clear expositions of the impact of within-person vs. between-person variability on bias and design of epidemiologic studies, motivated by problems in nutritional and occupational epidemiology [2–4]. These study design considerations led to numerous developments in optimization of exposure assessment approaches, which are applicable (and were informed by) consideration of biomarkers of exposure [5]. Classical measurement error in exposure degrades statistical power, resulting in a need to increase sample sizes to reflect the expected correlation between true and measured exposures (validity coefficient) [6, 7]; the loss of power also occurs due to misclassification of binary classifiers of exposure [8, 9]. In univariate regression models, classical non-differential error in estimated exposures causes the expected slope that we estimate to be shallower (closer to zero) than the true one, implying that measurement error tends to make it harder to detect associations in such models. It is less well appreciated (although not controversial) that classical measurement error can also produce spurious associations [9, 10]. With respect to the impact of low power per se, it is important to recall that it both reduces “the chance of detecting a true effect” and “the likelihood that a statistically significant result reflects a true effect” [11]. Despite these issues being known to specialists for decades, there is a lack of user-friendly software to design studies with sufficient power accommodate the potential biases that arise during the use of individual-based exposure assessment.

Our work is specifically motivated by epidemiologic studies that use biomarkers of exposures. The use of biomonitoring to estimate exposure in epidemiology has exploded in recent decades as new analytical techniques have become available to measure increasingly small concentrations of analytes in biological media [12]. We assume here that sampling and analytical techniques have negligible errors, orders of magnitude smaller than those related to between- and within-person variability in exposure and exposure biomarker values. Biomarkers of exposure are often seen as advantageous over measures of external exposure because they provide an integrated measure of absorbed dose and can be less variable, fluctuating at random around true long-run average values to a much lesser degree than the changes in environmental conditions due to “physiological dampening” [13, 14].

When biomonitoring first started being used in epidemiology studies, the compounds of interest had biomarkers with relatively long half-lives ranging from months (lead and other heavy metals) to years (dioxins, PCBs, etc.). More recently, studies have started to use biomonitoring to quantify exposures for substances with short-lived biomarkers that have half-lives in the order of minutes to a few days [15]. Many of these short-lived compounds have substantial intra-individual variability, especially in urine [16–19]. For some compounds, including phthalates and bisphenol-A (BP-A), the concentration of parent compounds or their metabolites can vary by several thousand-fold within an individual in a single day. Thus, for some compounds, taking a single urine sample from an individual is likely to yield highly uncertain estimates of the person’s typical (average) exposures over the time intervals of interest for most types of effects being studied, e.g., cancer (lifetime) and birth outcomes (months).

To describe different combinations of within- and between-person variability, it is convenient to compute the intraclass correlation coefficient (ICC), defined as the ratio of between-person to total variance [20]:

1 $$\mathrm{I}\mathrm{C}\mathrm{C}=\sigma_\mathrm{B}^2/(\sigma_\mathrm{B}^2+\sigma_\mathrm{W}^2)$$

where σ^2^_B_ is between-person and σ^2^_W_ is within-person variance. The value of the ICC can range from 0 (if σ^2^_B_ = 0) or near 0 (as σ^2^_W_ becomes very large compared to σ^2^_B_) to 1 (as σ^2^_B_ becomes very large compared to σ^2^_W_). Importantly, ICC is related to the correlation of true and measured value. The correlation is measured using a validity coefficient (ρ). The validity coefficient is given by

2 $$\rho^{2}=\sigma_\mathrm{B}^2/(\sigma_\mathrm{B}^2+\sigma_\mathrm{W}^2/m)$$

and is equal to the ICC only when one measurement is collected (m = 1). As the equation indicates, the more exposure measurements collected for a person the closer the validity coefficient approaches 1, since the impact of within person variation on the estimate of the person’s average exposure is reduced, and tends towards zero. Pleil & Sobus [21] discussed implications of ICC value in biomarkers for lifetime risk assessment, including consideration of the value of adequate number of repeated measurements. While the ICC is specific to a chemical, it can also vary as a function of the length of time the biomarker was measured (such as a 24-hour composite urine sample versus a spot urine sample) and method used for individual hydration status correction. For example, LaKind et al. [15] reported ICCs that ranged from 0.18 to 0.91 for monoethyl phthalate.

The availability of practical software to determine the sample sizes needed for studies of individuals’ exposures when there is within-person variance are crucial in epidemiological research. Traditional approaches to power and sample size calculations account for variability between individuals but do not account for the variability of exposures both within and between individuals. Not accounting for the interplay of between- and within-person variability in exposure can lead to study designs that fail to achieve desired power and that may be unacceptably biased by the expected impact of measurement error in exposures. To address these concerns, we aimed to developed specialized online tools (“calculators”) to estimate power and bias in linear and logistic exposure-response models, accounting for classical measurement error with independent, normally distributed, additive, and homogeneous errors in exposure. The primary problem we addressed is the need for calculators that enable researchers to determine adequate sample sizes in terms of the number of individuals sampled (*n*) and number of repeated samples per individual (*m*) in the presence of measurement error in exposure, and to anticipate biases in effect estimates that may arise from given study design choices. The calculators should also help in designing a study by selecting the optimal (*n* and *m*) combination to maximize power given a budget and time constraint.

The purpose of this paper is to consider practical statistical methods for estimating sample sizes (numbers of individuals and number of samples per individual) to obtain desired control for errors and acceptable bias in the estimates of exposure-response associations from individual-level based exposure assessment in epidemiology. We assume a classical measurement error model throughout. Additional file 1 presents the formal measurement error model that we consider.

To ensure that the calculators were useful and accurate, a panel of experts in epidemiology, biostatistics and exposure assessment reviewed the calculators to ensure user-friendly design, proper use of statistical approaches and vetted results. We further asked the panel of experts to help develop best practices for the use of biomonitoring in epidemiology studies. The findings of the panel (reported in the appendices of this paper) along with the publicly available calculators will provide researchers with valuable tools to help assure epidemiology studies are conducted with sufficient power to support the tested hypotheses.

## Methods

The project proceeded in three phases. First, the calculators were developed by two of the authors (IB and LAC). Next, an independent panel of experts reviewed the calculators and answered charge questions about best practices for the use of biomonitoring in epidemiology studies. Lastly, the calculators were refined based on input from the expert panel.

### Phase 1: development of calculators

Additional file 2 provides the R [22] computer code and user guides for the following four calculators, and hyperlinks to each calculator are given in text. The Digital Object Identifiers (DOI) for calculators are 10.63565/scipinion.resource.dvc for the calculator for the desired validity coefficient (Calculator V),10.63565/scipinion.resource.lin-n for the minimum detectable effect for linear regression: selecting the number of subjects, n, (LIN-N), 10.63565/scipinion.resource.lin-m for the minimum detectable effect calculator for linear regression: selecting the number of samples per individual, m, (LIN-M), and 10.63565/scipinion.resource.logit-pb for power and bias of logistic regression (LOGIT-PB).

#### The Desired Validity Coefficient (calculator V)

 (https://scipinion.shinyapps.io/RepeatsForDVC/*)*

Suppose that a researcher planning a study’s budget and logistics wants to know how many samples (e.g., voided urine samples) must be collected from an individual to achieve a desired level of validity in the person’s true mean exposure (e.g. typical urine concentration during specified time). This calculator estimates the number of repeated measurements per person (*m*) needed to achieve a desired validity coefficient (ρ) for a given value of ICC. The key formula used in this calculator is given by Fleiss [20] in expression (1.31):

3 $$m=\left(\mathrm \rho^2\times\left(1-\mathrm{ICC}\right)\right)/\left(\mathrm{ICC}\times\left(1-\mathrm \rho^2\right)\right)$$

#### Minimum Detectable Effect (MDE) Calculators for Linear Regression: optimizing number of subjects (calculator LIN-N) and Minimum Detectable Effect (MDE) Calculators for Linear Regression: optimizing number of samples per individual (calculator LIN-M)

(https://scipinion.shinyapps.io/LinearRegressionN/*)*

(https://scipinion.shinyapps.io/LinearRegressionM/)

Imagine that a researcher is designing a study to estimate the relationship between exposure and a health outcome where both are measured on a continuous scale and both measurements can be transformed to follow normal distribution. Such a study would have to demonstrate that it can achieve sufficient power without placing undue burden on participants to be deemed ethical. How many subjects should one enroll in (e.g. newborns, *n*) and how often (*m*) should one measure a biomarker of exposure of interest in the mother during pregnancy at the time of etiological significance. This calculator evaluates the tradeoff between the number of subjects (*n*_*z*_), the number of repeated measurements per subject (*m*) and the minimum detectable effect size (when the null hypothesis is slope = 0) in linear regression models, accounting for classical additive measurement error. If we want to achieve user-specified levels of statistical significance and power (Type 1 and Type 2 errors), the primary formula [6, 7] is.

4 $$n_z=n_x/\rho^2$$

where *n*_*x*_ is the number of subjects in a sufficiently powered study if there was no measurement error [23].

If we fix *n*_*z*_, we can then calculate corresponding minimum detectable effect size as a function of number of repeats per person (*m*) via rearrangement of the equation that gives us the sample size (see Additional file 2 for details). In essence, when there is classical measurement error in exposure, a study can only detect an effect that is larger than that implied by the study of the same size without measurement error in exposure. This is consistent with a well-known property of non-differential classical measurement error that causes the observed slope of a simple linear regression line to be closer to zero (attenuation) than the true slope in proportion to the ratio of within- and between-person variances in exposure and *m*. If the true slope is equal to β, then the expected slope is.

5 $$\beta/\left[1+\sigma_w^2/\left(m\times \sigma_\mathrm{B}^2\right)\right]$$

Thus, the greater the *m* is, the closer the observed slope is to β [4]. Simply put, classical measurement error in exposure makes it harder to detect smaller slopes, making the minimum detectable effect greater than if there were no measurement error. We can re-arrange equation from [4] for expected bias due to random error to solve for percent bias:

6 $$\left(1-{m\times \sigma_B^2}/\left(m{\mathit\times \sigma_B^2}+ \sigma_W^2\right)\right)\times100\%$$

and again note that bias increases with greater within-person variance and decreases with larger number of repeated measurements *m*. It is essential to realize that the equation does not address the impact of measurement error on the variance of the estimated slope, which is too narrow if we ignore measurement error [24] (Sect. 2.5, p21-23).

#### Power and Bias Calculator for Logistic Regression (calculator LOGIT-PB)

 (https://scipinion.shinyapps.io/SensitivityAnalysisExplorer/*)*

Now suppose that a researcher is designing a study to estimate the relationship between a continuous exposure variable that can be transformed to follow a normal distribution and some health outcome that is captured as either present or absent (e.g. pre-eclampsia). This calculator examines the sensitivity of sample size and power to measurement error in exposure in logistic regression models, typically employed for binary outcomes in epidemiology. Unlike linear regression, there is no closed form solution for bias in the logistic regression under classical measurement error model; we therefore use numerical simulations [24]. This calculator simulates the power and bias of parameter estimates under varying conditions of between-person variance, within-person variance, true odds ratio (OR), and background incidence (p_0_), by adapting the simulation framework and code approach of Lee & Burstyn [25]; for simplicity, we fix Type 1 error at less than 5%. The simulation process involves generating data based on specified parameters and fitting logistic regression models to estimate the power and bias.

### Phase 2: external expert peer review and recommendations

An expert panel was recruited and engaged utilizing the methods described in Kirman et al. (2019) as modified in Additional file 3. Multiple design elements were included in this review to minimize potential sources of bias and groupthink, and to improve transparency of the review. These elements include the following: (1) a triple-blinded process, in which the review sponsor was blinded to the panel members, panel members were blinded to the sponsor, and panel members were blinded to one another (e.g., identified only as “Expert 1”, “Expert 2”, etc.) during all online deliberations; (2) a three-round, modified Delphi format was adopted for the review to collect both independent and deliberative input from the topic experts in an effort to minimize potential groupthink; (3) individual responses and comments from the panelists were recorded and are provided in their entirety (Additional file 3) to ensure transparency, and minimize potential reporting bias; and (4) although individual responses are provided, they are attributed to panelists’ anonymous display names (e.g., to Expert 1, Expert 2, etc.) rather than to specific panelist identities in an effort to provide psychological safety (i.e., scientists should feel free to express their scientific opinions without fear of negative repercussions).

We provided the experts with a white paper that summarized the calculators, the science behind them, and a brief synopsis of the scientific issues associated with the use of biomonitoring data in epidemiology studies (Additional file 4). The experts were also provided with links so they could try each of the calculators for themselves. The experts took part in three rounds of review. Round 1 involved the experts reviewing the white paper and answering charge questions centered on the validity and usefulness of the calculators. During Round 2, the experts reviewed each other’s answers and debated with each other. Round 3 allowed the experts to revisit their answers from Round 1 in case they learned anything from other panelists that made them want to change their answers. In addition, Round 3 included additional charge questions designed to elicit opinions on the best practices for the use of biomonitoring data in epidemiology studies.

Participating experts were from Australia, North America, and Western Europe. All had doctoral degrees, and hold or held (retired) academic positions ranging from Assistant Professor to senior level administrative and research posts. These experts had 13 to 45 years of experience and individually have published from 23 to 550 peer-reviewed manuscripts at the time of recruitment. The experts included Yang Cao (Örebro University, Sweden), Guy Eslick (The University of Sydney, Australia), Shelley Harris (University of Toronto, Canada), Leeka Kheifets (University of California at Los Angeles), Michael Kramer (McGill University, Canada), Peter Langlois (University of Texas, USA), Paul Lee (University of Southampton, UK), Boris Reiss (KU Leuven, Belgium), Trudy Voortman (Erasmus University Medical Center, The Netherlands).

## Results

### Recommendations of expert pannel

The full record of input from the experts is given in Additional file 3; key recommendations for best practices are summarized in Tables 1 and 2.

**Table 1:** Summary of responses of the nine experts to “How important would you consider the following practices for the design of epidemiology studies that rely on biomonitoring for exposure assessments? (rate from not at all important (1) to very important (5) on visual analogue scale)”

Response Option	Mean	Standard Deviation
Conduct a pilot study to estimate variance components of the biomarker of exposure (when not already known)	4.4	1.0
Identify a hypothesized minimal effect size (in the outcomes measure) that the researcher intends to be able to identify	4.3	1.3
Estimate the impact on bias and power of measurement error in outcome, i.e. within-person variability in outcome	4.1	0.9
For a given exposure of interest, select biomarkers with the largest intraclass correlation coefficient (ICC) (e.g., select biomarkers with the longest half-life)	3.9	0.6
Consider study designs that do not rely only on contrasts in exposures among individuals but instead seek groups of individuals with very different exposures	4.0	0.7
Articulate the hypothesized causal diagram (DAG) that includes variability in biomarkers of exposure between- and within-persons	3.6	1.0
Calculate the efficiency, in terms of costs, of investing into more measurements within person versus more persons to minimize bias (as was done by Armstrong (1996))	3.8	1.0
Use pools of biofluids from each individual to help minimize within-person variability and increase ICC	2.8	1.0

**Table 2:** Summary of responses of the nine experts to “How important would you consider the following practices for the analysis of epidemiology studies that rely on biomonitoring for exposure assessments? (rate from not at all important (1) to very important (5) on visual analogue scale)”

Response Option	Mean	Standard Deviation
Adjust for bias due to measurement error in exposure	4.3	1.3
Adjust for bias due to measurement error in covariates	3.8	1.0
Adjust for bias due to measurement error in outcome	4.3	1.1
Adjust for multiple comparisons	3.3	1.3
Quantitatively account for unmeasured confounding	3.3	1.1
Employ Bayesian methods that quantitatively integrate prior knowledge on the topic	3.7	0.7

The experts opined that our calculations were sound and well-motivated, and that the graphical user interfaces (GUIs) and usability of the calculators were of high quality. We implemented several of the experts’ recommendations to improve the GUI, including greater clarity on units of input and output parameters. The calculator for assessing sensitivity of power and bias on simple logistic regression model to errors in exposure estimates was refined based on the panel’s suggestion for greater clarity in the results. Some additional suggestions, namely developing calculators that can address multiplicative measurement error, were implemented in a follow-up effort (to be published later). No other substantiative recommendations were made by the panel (Additional file 3). The experts agreed that our calculators can help to improve the design of epidemiological studies, and that they address an unmet need of practicing epidemiologists without easy access to the expertise of statisticians or appropriate statistical software.

Several experts expressed interest in adding consideration of confounding to our calculators, including the consideration of measurement error in confounders, as well as additional measurement error models (e.g. Berkson), exposure distributions (e.g. log-normal), and exposure-outcome models (e.g. Poisson and Cox Proportional Hazards regressions). There was a discussion of how to incorporate measurement error in outcome measures, which is critical to validity of any epidemiological study. The matter of measurement error in effect modifiers was also a concern but there was recognition that such matters may be best dealt with on a case-by-case basis.

### Best practices for epidemiology studies that utilize biomonitoring

The best practices recommended by the experts are summarized in Table 1. The experts agreed that pilot studies that assess ICC would be an essential component of the best practices in design of epidemiologic studies that use biomarkers; any such studies should report estimated variance components. There was strong support among the experts for identifying a minimal effect size that the researcher intends to be able to identify with stated statistical power during study design. The experts believed it is important to estimate the impact on bias and power of measurement error in outcome, i.e. within-person variability in outcome. Currently, measurement error in the outcome is captured by the variance of the outcome in our calculator for linear regression and therefore its impact on power can be studied, if uncertainty in outcome measure is not related to the level of exposure or any other sources of error. The experts favored a priori selection of the most accurate outcome measure as well as biomarkers of exposure with the largest ICC. Experts were challenged to consider study designs that do not rely only on contrasts in exposures among individuals but instead seek groups of individuals with very different exposures. They support consideration of such group-based exposure assessments if they reduce bias and improve power, along the lines of arguments in [4, 26, 27].

The experts agreed on several methodological principles, including the following:

- Adjusting for all known sources of error is advisable, with adjustment for errors in exposures and outcomes appearing to have the most support (Table 2).
- The typical amount of expected bias due to measurement error in effect estimate was agreed by the experts to lie between 5 and 10% of the hypothesized true value, with larger bias being seen as an impetus to adjust for measurement error in analysis (e.g., via methods summarized in [28, 29]).
- Rigid adherence to cutoffs of p-values in data analysis is ill-advised. Instead, the experts recommend considering Type 1 error rates in the study design between 0.01 (confirmatory study in which previously suspected association is evaluated) to 0.1 (exploratory study in which we assess associations that may deserve more extensive evaluation).
- Experts expressed preference to rely on confidence intervals instead of p-values in interpreting results of epidemiologic studies.
- With respect to power, experts recommended designing studies that rely on biomarkers of exposure to have more power than the usual default of 80% and strive for power to be closer to 90%.

### Illustrative application to environmental exposure and reproductive health

Birth cohort studies collect data on environmental exposures and pregnancy outcomes. These datasets include repeated measurements of biomarkers of exposure in urine, with samples collected at each of the three trimesters (*m* = 3).

First, we focus on the paper by Sol et al. [30] as an illustration of how our calculators may be useful. Sol et al. [30] analyzed impact of bisphenols (BP) on the outcomes of 1,379 pregnancies, reporting (among others):

The effects (slopes) on standard deviation of fetal head circumference from linear models for an interquartile range increase in each natural log-transformed pregnancy-averaged concentrations of urinary biomarkers of bisphenols are shown in Table 3 of [30], with the most notable effect estimate for BP-S of 0.18 (95% confidence interval (CI): 0.01, 0.34) (*m* = 2); for BP-A, the estimated slope was − 0.12 (95% CI: −0.27, 0.03) (*m* = 3).

**Table 3:** Key parameters of the two case studies and recommendations for better design for continuous outcomes; n = number of subjects, m = number of repeated measurements per subject, ICC = intraclass correlation coefficient, MDE = minimum detectable effect; see text for details and results with expanded range of values

Study	Actual				repeats (m) required for validity coefficient		bias (%) in slope for actual *n* for design m		*n* for power of 90% and cut-off *p*-value of ≤ 0.01?			MDE for actual *n*	
					Validity coefficient		m	bias	m	ICC	*n*	m	
	*n*	m	ICC	effect	0.7	0.9							
Sol et al. [30]	1379	2	0.2	0.1	4	18	4	46%	2	0.2	3027	2	0.3
Guo et al. [31]	377	1	0.6	0.25	1	3	1	40%	1	0.6	122,062	1	4.2
		1	0.4	−0.37	2	7	1	60%	1	0.4	174,640	1	−8.7

ORs that reflect the risk of preterm birth and small size for gestational age at birth for an interquartile range increase in each natural log-transformed pregnancy-averaged concentrations of urinary biomarkers of bisphenols are shown in Table S6 of [30], with the most notable estimated OR for BP-S of 0.76 (95% CI: 0.58, 1.00) (*m* = 2) for risk of being small for gestational age; for BP-A, the estimated OR was 1.10 (95% CI: 0.88, 1.37) (*m* = 3).

The interquartile ranges of pregnancy-averaged levels of BP-S and BP-A are not reported, but we take them to be approximated by the first trimester values: 0.13 to 2.4 for BP-S and 1.1 to 12 for BP-A; this is roughly equal to 2.5-unit change in natural logarithm of concentrations across the interquartile range.

According to [19], for BP-A $\sigma_{\mathrm B}^2\;=\;0.21\;$ and $\sigma_{\mathrm W}^2\;=\;0.72$, yielding ICC = 0.21/(0.21 + 0.72) = 0.23. We will assume that this level of variability is a reasonable estimate for BP-S as well in [30].

Second, we consider a prospective birth cohort analyzed by Guo et al. [31], who analyzed the impacts of in-utero and early life exposure to triclosan on neurodevelopment at 3 years among 377 mother-child pairs. Exposure to triclosan was estimated from spot urinary measurements, one collected from mothers on the delivery day, and around age of 3 years on children, at the same time as neurodevelopment was evaluated. Separate statistical analyses by linear regression were conducted to examine the effects of maternal and children’s exposure (*m* = 1). Guo et al. [31] reported that they observed associations to be confined to 184 boys:

- Maternal urinary triclosan levels were associated with motor function, with the estimated slope of 0.25 (95%CI: 0.01, 0.50).
- Urinary triclosan concentrations at 3 years of age were with social development, with a slope of −0.37 (95%CI: −0.72, −0.03).

The above-cited estimated slopes are per 1 unit of log-transformed biomarker measurements. From a related publication from the same cohort [32], we learn that outcome measures in question had a standard deviation of 6. Guo et al. [31] reviewed literature that claimed that the ICC of urinary triclosan concentration during pregnancy takes on values of 0.4 to 0.6, while lower values are expected for children, on the order of 0.2 to 0.4. We note that these estimates are lower than those quoted in LaKind et al. [15] but accept that the ICC may depend on population. In our analysis, we assume best case scenario (in a sense of higher expect power and lower bias) and fix ICC for measurements of pregnant women at 0.6, and their 3-year-old children -- at 0.4. Guo et al. [31] report levels of triclosan by sex of child in a manner that allows us to estimate total logarithmic variances from the reported 95% CI of urinary measurements as 0.019 for mothers and 0.0095 for their sons. This is a total variance (σ^2^_B_ + σ^2^_W_) that is not separated into its within- and between-person components. Yet again, assuming the best-case scenarios, we will treat these variances as being equal to between-person variances (σ^2^_B_). This implies that for mothers, the σ^2^_W_ = 0.013 for mothers and 0.014 for their sons.

The summary of key analyses for continuous outcomes that employed our software is given in Table 3. The table lists “actual” values used in the two case studies and various recommended design feature that would be sought for a confirmatory study based on these analyses. We observe that both Sol et al. [30] and Guo et al. [31]

- required more repeated measurements per subject in order to achieve validity coefficients of 0.7 or greater,
- have expected bias in the slope of linear regression that is at least four times larger than the 10% threshold that is deemed acceptable by our expert panel,
- require many more subjects than were studied using actual number of repeated measurements to achieve recommended power, and.
- were designed to detect much larger effect sizes than those that were actually reported.

Details are elaborated below. Similar table for binary outcomes (logistic regression) was not constructed, because that analysis is far simpler than that for continuous outcomes.

#### How many repeats m do we need to obtain a desired validity coefficient of either 0.7 or 0.9

Our *Calculator V* allows for inputs of ICC of 0.2 or 0.3 (for analysis of Sol et al. [30], and 0.4 and 0.6 (for analysis of Guo et al. [31]).

If ICC = 0.2, the required numbers of repeats are 4 and 18, respectively. If ICC = 0.3, the required numbers of repeats are 3 and 10, respectively. This implies that 3 to 4 repeats per woman during pregnancy is the minimum for a high-quality study of the sort conducted by Sol et al. [30] for a desired validity coefficient of 0.7, with 10 to 18 repeats for a validity coefficient of 0.9.

If ICC = 0.4, the required numbers of repeats are 2 and 7, respectively. If ICC = 0.6, the required numbers of repeats are 1 and 3, respectively. This implies that 1 to 3 repeats per woman during pregnancy (ICC = 0.6) is the minimum for a high-quality study of the sort conducted by Guo et al. [31] for a desired validity coefficient of 0.7 to 0.9. Likewise, this implies that 2 to 7 repeats per child (ICC = 0.4) is the minimum for a high-quality study of the sort conducted by Guo et al. (2020) for a desired validity coefficient of 0.7 to 0.9.

#### How many repeats m do we need to have no more than 10% expected attenuation bias in slope of a linear regression?

To have an attenuation bias of no more than 10% in the slope of simple linear regression, we must have 1- *m ×* σ^2^_B_/(*m ×* σ^2^_B_ + σ^2^_W_) ≤ 10%. In the case study of Sol et al. [30], when *m* = 4, percent bias is 1 - (4* × *0.72/(*4 × *0.72 + 0.21)) = 46%. A bias of 10% or less is achieved with m ≥ 30. In the case study of Guo et al. [31] when *m* = 1, the expected percent bias in slopes for analysis of exposure in boys is at least 60% and, in their mothers, – 40%.Therefore, based on the recommendations of the experts we consulted, it would be necessary to adjust for bias due to measurement error when data in the two case studies are analyzed.

**Fig. 1:**
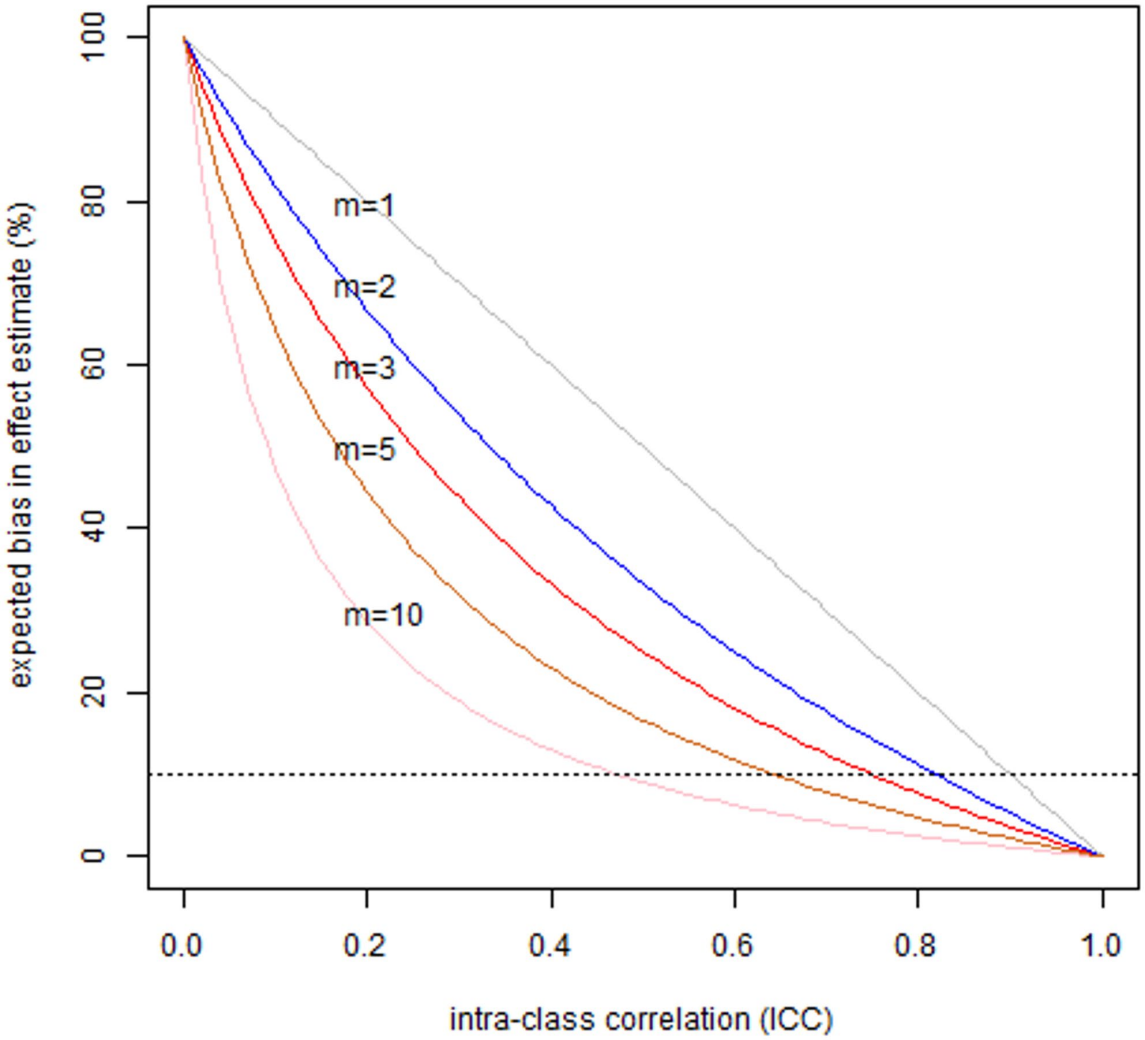
Expected bias in slope of linear regression in relation to intra-class-correlation coefficient of exposure measure, modulated by the number of repeated measurements of exposure on a person (*m*); the horizontal dashed line denotes 10% bias above which the expert panel recommends adjustment for bias during data analysis

Figure 1 illustrates a more general case of the interplay between the number of repeated measurements, ICC and expected bias in slope of linear regression. It seems apparent that with realistic (or typical) numbers of repeated measurements of less than 5, ICC should be at least 0.7 to have expected bias in slope under 10%.

#### How many subjects do we need to enroll in the study, if we are contemplating having 1 to 10 repeats with a continuous outcome, and aim to have power of 90% and cut-off *p*-value of ≤ 0.01?

To answer this question, we use *Calculator LIN-N*.

For analysis of Sol et al. [30], we input variance of the outcome as 1, given that z-scores of birth weight standardized for sex and gestational age, by definition, have mean zero and variance 1, and follow a normal distribution. Within- and between-variances in BP-A are as above. We fix the minimum detectable effect at 0.25 (one quarter of a standard deviation per unit of *ln*(exposure)), a far stronger one than those reported by [30]. Then we fix Type 1 and 2 error rates and alter *m*.

- If *m* = 1, then we require 4,955 subjects (and measurements),
- If *m* = 2, then we require 3,037 subjects (and 3,037 × 2 = 6,074 total measurements),
- if *m* = 10, then we require 1,502 subjects (and 1502 × 10 = 15,020 total measurements).

It appears that an unusually large study with intense data collection would be needed to detect a strong effect in a confirmatory study of the effect of BP-A on fetal growth, e.g. twice the number of pregnancies than those collected in [30], if only two repeated measurements were collected per woman.

For analysis of Guo et al. [31], we input variance of the outcome as 6 (see above). Within- and between-variances for the two triclosan measurements are as above. We fix the minimum detectable effect at 0.25 for association with exposure in mothers and − 0.37 for association with exposure in boys.

For study of associations with exposure in mothers:

- If *m* = 1, then we require 122,062 subjects (and measurements),
- If *m* = 2, then we require 97,650 subjects (and 97,650 × 2 = 195,300 total measurements),
- if *m* = 10, then we require 78,120 subjects (and 78,120 × 10 = 781,200 total measurements).

For study of associations with exposure in the boys:

- If *m* = 1, then we require 174,640 subjects (and measurements),
- If *m* = 2, then we require 122,248 subjects (and 122,248 × 2 = 244,496 total measurements),
- If *m* = 10, then we require 80,334 subjects (and 80,334 × 10 = 803,340 total measurements).

**Fig. 2:**
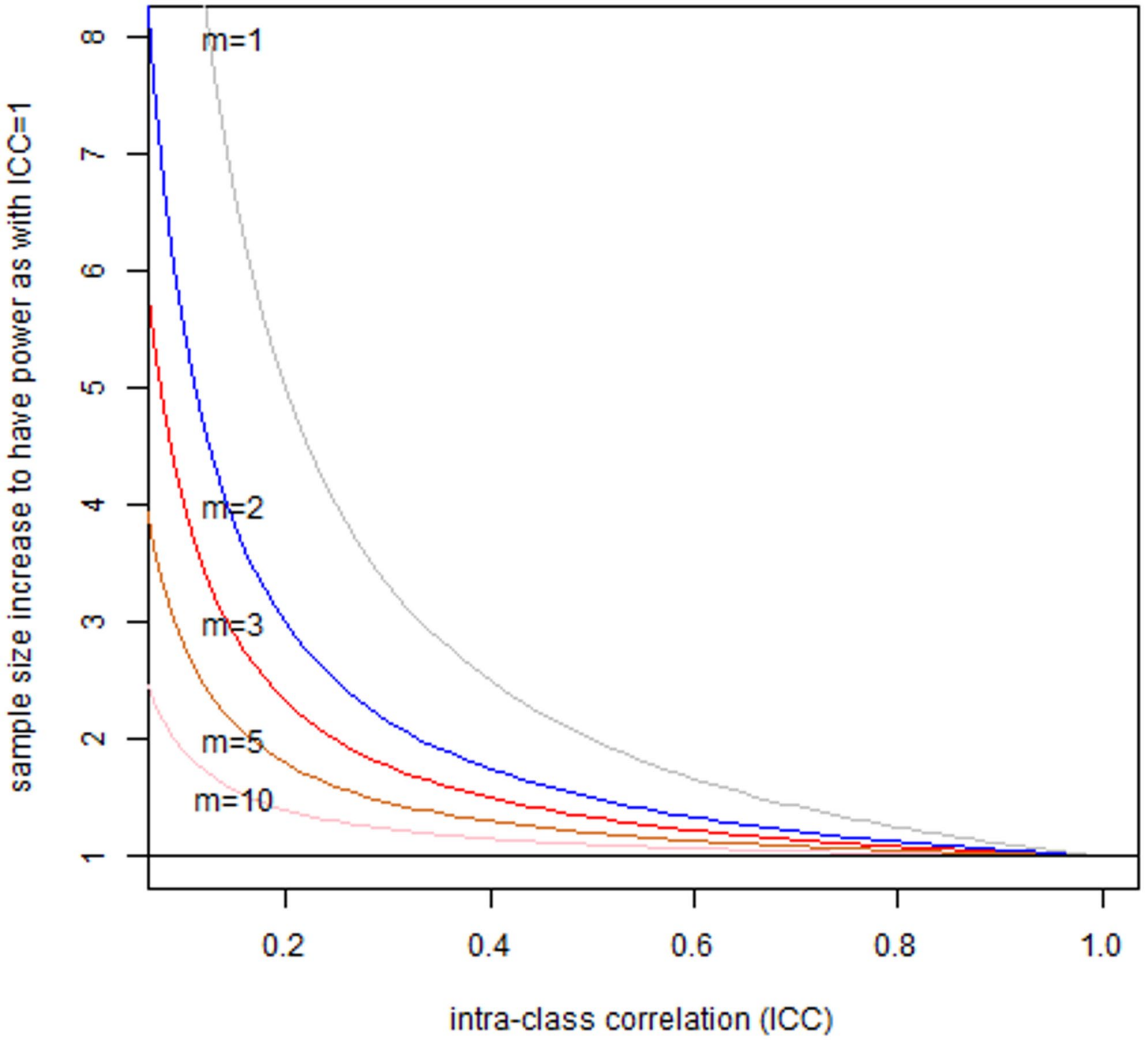
Sample size (number of persons) increased (*n*_*z*_*/n*_*x*_) need to maintain power of the study designed under the assumption of no measurement error or no within person variability (ICC = 1), modulated by the number of repeated measurements of exposure on a person (*m*)

A more general case of how many more people one needs to enroll to have the same power as when there is no within-person variability in relation to ICC (i.e., ICC = 1) and the number of repeated measurements is illustrated in Fig. 2. It appears that for realistic (or typical) numbers of repeated measurements of less than 5, ICC must be more than 0.2 or 0.3 before the number of participants needs to be doubled to maintain desired power.

#### If we can conduct a study that is of a fixed size, what would be the minimum detectable effect?

We can apply *Calculator LIN-M* to understand the relationship between number of measurements and minimum detectable effect size to address this question. We fix variance components as above and wish to have 90% power and cut-off p-value of ≤ 0.01, as recommended by the expert panel for a confirmatory study. With respect to Sol et al. [30] with *n* = 1,379, as we consider 1, 2, and 10 numbers of repeats, the minimum detectable effects per *ln*(exposure) are 0.42, 0.30, and 0.13. To put this into context, the largest effect of bisphenols on features of fetal growth assessed as percentiles of expected values was estimated in [30] to be on the order of 0.1. Thus, the study of Sol et al. [30] was not designed and powered to detect even the large associations that they report. To put this into context, the largest effect of bisphenols on features of fetal growth assessed as percentiles of expected values was estimated in [30] to be on the order of 0.1.

With respect to Guo et al. [31] with *n* = 184, as we consider 1, 2, and 10 numbers of repeats, the minimum detectable effects per *ln*(exposure) are 4.2, 3.0, and 1.3 for the effects due to exposure of mothers, and − 8.7, −6.1, and − 2.7 for the exposure at age 3 in boys. All the reported effects for boys in Guo et al., even considering the upper bound of the 95%CI is less than 1.

Thus, the studies of Sol et al. [30] and Guo at al. [31] were not designed and powered to detect even the large associations that their results are consistent with.

If we consider Sol et al. [30] to be exploratory, with power of 80% and *p* ≤ 0.05, then the three repeats that they planned to collect imply a minimum detectable effect of 0.18, more than twice that reported for PB-S and fetal head circumference (see above). If we consider Guo et al. [31] to be exploratory, with power of 80% and *p* ≤ 0.05, then the one repeats that they planned to collect imply a minimum detectable effect of 3 for exposure in mothers and − 6 for exposure in children, an order of magnitude greater than the reported slopes of 0.25 and − 0.37.

#### We are now considering designing a study of binary outcomes

We turn to binary outcomes in [30] such as the odds of being born small for gestational age (and sex) using *Calculator LOGIT-PB*. This outcome, by definition, occurs with a baseline incidence of 10% (p_0_). We fixed the significance level (Type 1 error) at 5% and variance components as above. We aim for a power of 90% (confirmatory analysis). The OR at the limits of 95% CI that is reported is on the order of 1.4 per inter-quartile range, which translates to about OR = 1.14 per *ln*(exposure) (= exp(log(1.4)/2.5)). We consider a sample size as in [30] (1,379 subjects), as well as twice that. The number of repeats is fixed at *m* = 2, that obtained for BP-S, where the strongest effect was reported [30]. (Note that the calculations for logistic regression reported here were performed using source code for the calculator, not the calculator itself, to ensure higher accuracy.) We estimated:

- with 1,379 subjects, the power is calculated to be 7% and median percent bias in OR of −8% (95% uncertainty interval − 26 to 15%),
- with 2,758 subjects (twice the number as in Sol et al. [30]), the power is calculated to be 9% and median percent bias in OR is −8% (95% uncertainty interval − 21 to 8%).

There is a clear possibility of bias in the OR of more than ± 10% once one considers performance in realistic sample sizes, not just the expected value of bias in OR. The power is far below nominal levels even after increasing the number of subjects ten-fold.

It is instructive to consider the expected power and bias in logistic regression when there is no true association (OR = 1) among 1,379 subjects and *m* = 2, as for BP-S in [30]. Here, using *Calculator* LOGIT-PB, the median percent bias in OR is 0.1% (95% uncertainty interval − 21 to 27%), implying that if there is no true effect, estimates of OR from 0.5 to 1.8 with 95% certainty for BP-S are consistent with [30]. This illustrates that in a study of low power with no true effect, reported estimates can vary widely around the null, suggesting possible harmful or protective effects on average, at random.

## Discussion

The results from our suite of calculators underscore the importance of the impact of measurement error on the design of studies in epidemiology. Combined with consultation with the panel of experts, we believe that best practices in design and conduct of epidemiological studies that use biomarkers should include the following elements:

- A pilot study to estimate variance components of the biomarker of exposure.
- A priori specified desired minimal effect size to detect with a stated probability (power).
- For a given exposure of interest, select biomarkers and compositing procedures with the largest intraclass correlation coefficient; there was some concern about using pooling procedures, even though they have been shown to perform well in controlling bias due to random errors [33–35].
- In estimating the power and bias of epidemiologic studies during the planning stages, one should estimate.
  - The impact of measurement error in exposure.
  - The impact of measurement error in the outcome.
  - The impact of measurement error in covariates (potential confounders).
- Consider study designs that do not rely only on contrasts in exposures among individuals but instead seek groups of individuals with very different exposures.
- Calculate the efficiency, in terms of costs, of investing in more measurements within person versus more persons to minimize bias (as was done by Armstrong [7])
- Articulate how much bias is tolerable before planning to adjust effect estimates for these biases during data analysis and reporting; methods to do so are accessible through collaboration with statisticians.
- Focus on confidence intervals rather than p-values in interpretation of study results.

Our calculators illustrate the complex interplay between power, number and allocation of measurements, and measurement error. For instance, while increasing the number of subjects generally enhances the power to detect true effects, it can also amplify the bias due to measurement error if not properly addressed by repeated measurements per person (or other adjustment techniques). Researchers must carefully consider these tradeoffs when designing studies to allocate measurements and other resources to achieve the desired power without introducing increasing bias due to measurement errors. It is also important to consider how burden on participants may affect participation in the study, creating a risk of selection bias.

While increasing the number of participants generally enhances the power of a study to detect associations, large studies with poorly controlled measurement error introduce their own problems. In such studies, the effect estimate is biased with an ever-increasing precision, such that eventually one has near-certainty (variance approaches zero) about an effect estimate that is far away from its true value [36]. This may be sufficient for obtaining qualitative answers, but not for risk analysis that requires quantification of the exposure-response gradient [37, 38]. In such situations, adjustment for bias in measurement error during data analysis is essential, and the expected degree of bias can guide choice for planning such analyses (e.g. our expert panel recommended a 10% bias or more as a threshold). It is prudent to consider sample size requirements for adjustment of estimates for measurement error, as was previously done for binary exposure misclassification in case-control studies [8].

While the benefits of increased precision and reliability are clear, the financial and logistical burdens of increasing samples per individual required to increase power and reduce bias should be considered. Researchers must weigh these costs against the potential for more accurate and valid results, exploring alternative strategies such as selecting populations with large differences in individual exposures (as is often done in occupational epidemiology [4, 26, 27]), exploring methods for pooling urine samples (e.g., 24-hour composites) to increase the ICC, and statistical adjustments for measurement error through collaboration with statisticians. The work of Armstrong [7] on “asymptotic relative efficiency” can be extended beyond classical measurement error structures and towards more complex models to explicitly calculate cost trade-offs for a wider range of epidemiologic designs. Such research is like calculations used to decide whether to resort to measurements of external exposure or biomarkers, e.g [39].

Our calculations were developed under assumptions that may not hold in all practical situations but are applicable when classical measurement error model in exposure holds and exposure-response can be validly estimated via either linear or logistic regressions. The only practical solution to situations where assumptions of classical measurement error in exposure and exposure-response models that we assume do not hold, is that we can offer is to encourage others to conduct analyses of power and bias via simulations. Our approach to logistic regression via simulations, where there is no closed form solution, may be a particularly useful blueprint for such extensions of our work. Our software does not account for confounding, effect modification, or measurement error in covariates or outcomes, a matter that was clearly identified in our internal review and is a fruitful area of future enhancements. The limitations of our calculators can be overcome by customizing our simulation-based framework (*Calculator* LOGIT-PB) to the specific needs of individual studies. For instance, our work can be expanded to consider multiple covariates, measurement error in the outcomes, a wide range of exposure distributions and shapes of exposure-response associations. However, in seeking such customizations of calculators that forecast power and bias, it becomes challenging to have a user-friendly GUI and short computing times. We plan to address some of these challenges as we have done with our calculator for logistic regression, but software cannot and should not replace collaboration with a statistician from the very genesis of the project.

Using our calculators to test the validity of claims from an existing study showed that some studies published in the peer-reviewed literature are underpowered and cannot support the claims being made. Studies that find a statistically significant relation between exposure and outcome while being under-powered raise concern about unrecognized confounding. For instance, when using the concentration of a chemical in urine as the exposure metric, the known relation between body mass index (BMI, a measure of obesity) and concentration of analyte in urine can be the more likely explanation for the observed relation [40], in particular for an endpoint related to birth outcomes that are known to be related to maternal obesity [41]. A confounder measured with uncertainty, e.g., BMI as a measure of obesity, even if adjusted for in statistical analysis, as was done in Sol et al., [30] can aggravate bias. The impact on the biomarker-outcome association may not merely incompletely control for confounding, but actually increase it, as per textbook of Gustafson (Sect. 2.4, p 18–21) [24]. It is very difficult to predict biases due to incomplete control for confounding, and therefore it is fruitful to develop simulation-based software to estimate the impact of latent confounding on power and bias in epidemiological studies.

## Conclusions

We conclude that it is essential to justify sample size and study design, while explicitly acknowledging that measurement error in exposure can arise from within-person variability in biomarkers of exposure. Making tools that enable relevant calculations should facilitate improvements in the current practices in epidemiology, especially for researchers who lack access to specialist expertise in statistics. If problems arising from large within-person variability are not addressed in design and analysis of epidemiologic data, it will be difficult to distinguish causal from spurious associations.

## Supplementary Information

Additional file 1. Error model and equations that is the basis of this work.

Additional file 2. R code for all four calculators.

Additional file 3. Full report from the SciPinion panel of experts.

Additional file 4. The white paper that served as the review material for the SciPinion panel of experts.

## Data Availability

All statistical calculators developed as part of this manuscript are available at https://scipinion.com/resources/.

## Abbreviations

BMI: Body mass index
BP: Bisphenol(s)
BP-A: Bisphenol-A
BP-S: Bisphenol-S
CI: Confidence interval
DOI: Digital Object Identifiers
GUI: Graphical user interface
ICC: Intraclass correlation coefficient
LIN-M: Calculator for minimum detectable effect for linear regression: selecting the number of samples per individual (m)
LIN-N: Calculator for minimum detectable effect for linear regression: selecting the number of subjects (n)
LOGIT-PB: Calculator for power and bias of logistic regression
M: Number of repeated measurements per subject/individual
MDE: Minimum detectable effect
N: Number of subjects
Nz: Number of subjects needed with measurement error
Nx: Number of subjects in a sufficiently powered study with no measurement error
OR: Odds ratio
p0: Background incidence
PCBs: Polychlorinated biphenyls
V: Calculator for desired validity coefficient
β: True slope (beta)
ρ: Validity coefficient (rho)
σ²B: Between-person variance
σ²W: Within-person variance
