# Supplementary material for: Improving the design of epidemiology studies that use biomonitoring for exposure assessment: a SciPinion panel recommendation
Source: BMC Med Res Methodol. 2026 Jan 12;26:29. doi: 10.1186/s12874-025-02753-5 (PMC12888676; doi:10.1186/s12874-025-02753-5)
Supplement: Supplementary file 1 — Additional file 1. Error model and equations that is the basis of this work. [file 12874_2025_2753_MOESM1_ESM.pdf]

Appendix A

In epidemiological studies that employ biomarkers of exposure, it is typical to collect multiple measurements of the biomarkers and average them, with the average value then being plugged into a statistical model that relates exposure estimates to the outcomes. Such approaches have a long history in epidemiologic research and predate use of biomarkers, when measurements of externally measured exposure, which is often even more variable than biomarkers, were averaged to reduce measurement error and associated biases (Preller, Kromhout et al. 1995, Tielemans, Kupper et al. 1998, Kim, Richardson et al. 2011). More formally, *random effects models* assume that biomarker measurements are generated by the following process:

$$Z_{ij} = \mu + \alpha_j + \varepsilon_{ij},$$

where Z_{ij} is the ith observation on the jth person, μ is an unobserved overall mean, assumed to be stationary over time, α_j is an unobserved random effect shared by all biomarker measurements of person j, and ε_{ij} is an unobserved error term. For the ith measurement on person j, the α_j and ε_{ij} are assumed to be normally distributed, have expected value zero, be independent of each other, have identical distributions, and remain stationary. The variance of α_j is denoted σ^2_B and the variance of ε_{ij} is denoted σ^2_W.

When a single sample is collected from each individual, its observed value is used as an exposure estimate in epidemiology ($m = 1$). If $m > 1$ samples are collected per individual, then their average for an individual is used as that person's exposure estimate, denoted as $\bar{Z}_J$. The following classical additive measurement error model relates $\bar{Z}_J$ to true unobserved exposure X_j:

$$\bar{Z}_J = X_j + \varepsilon_Z,$$

where X_j and ε_Z are assumed to be independent; exposure is assumed to be normally distributed in the population with a fixed, stationary individual mean $\mu + \alpha_j$, and with a constant inter-individual variance V_b, $X_j \sim N(\mu + \alpha_j, \sigma^2_B)$; and the error for individual measurements is assumed to be normally distributed with zero mean and constant

variance, $\varepsilon_Z \sim N(0, \sigma^2_e)$, with $\sigma^2_e = \sigma^2_W/m$. Note that the correlation (validity coefficient of $\bar{Z}_j$) is $\rho_{xz} = (\sigma^2_B /(\sigma^2_B + \sigma^2_W /m))^{0.5}$, and it follows that $\rho^2_{xz} \neq$ ICC, unless $m = 1$; following Fleiss (Fleiss 1986) we call ρ^2_{xz} the "reliability of the mean of *m* independent replicate measurements, or "reliability" for short, and denote it by R_m. In the manuscript, ρ^2_{xz} is abbreviated to ρ.

Fleiss, J. L. (1986). The Design and Analysis of Clinical Experiments. New York, NY, John Wiley & Sons.

Kim, H. M., D. Richardson, D. Loomis, M. vanTongeren and I. Burstyn (2011). "Bias in the estimation of exposure effects with individual- or group-based exposure assessment." J.Expo.Sci.Environ.Epidemiol. **21**(2): 212-221.

Preller, L., H. Kromhout, D. Heederik and M. J. Tielen (1995). "Modeling long-term average exposure in occupational exposure-response analysis." Scand.J.Work Environ.Health **12(6)**: 504-512.

Tielemans, E., L. L. Kupper, H. Kromhout, D. Heederik and R. Houba (1998). "Individual-based and group-based occupational exposure assessment: Some equations to evaluate different strategies." Ann.Occup.Hyg. **42(2)**: 115-119.
