# Supplementary material for: Improving the design of epidemiology studies that use biomonitoring for exposure assessment: a SciPinion panel recommendation
Source: BMC Med Res Methodol. 2026 Jan 12;26:29. doi: 10.1186/s12874-025-02753-5 (PMC12888676; doi:10.1186/s12874-025-02753-5)
Supplement: Supplementary file 2 — Additional file 2. R code for all four calculators. [file 12874_2025_2753_MOESM2_ESM.pdf]

Appendix B: R Code for Calculators

```
# Calculator 1 The Desired Validity Coefficient (calculator V)

# https://scipinion.shinyapps.io/RepeatsForDVC/

# 10.63565/scipinion.resource.dvc

library(shiny)

library(ggplot2)

# Define UI

ui <- fluidPage(

  div(class = "container",

      div(class = "content",

        h1("Desired Validity Coefficient (V)"),

        p(class = "lead", "Calculate the required number of repeats based on ICC and desired validity coefficient"),

        sidebarLayout(

          sidebarPanel(

            sliderInput("icc", "Intraclass Correlation Coefficient (ICC):", min = 0.00, max = 1.0, value = 0.3, step = 0.01,

                        animate = animationOptions(interval = 300, loop = FALSE), width = '100%'),

            sliderInput("rhoXW", "Desired Validity Coefficient (rhoXW):", min = 0.1, max = 0.975, value = 0.85, step = 0.05,

                        animate = animationOptions(interval = 300, loop = FALSE), width = '100%'),

            numericInput("cost_per_measurement", "Cost per Measurement:", value = 10, min = 0, step = 1),
```

```
            numericInput("cost_per_participant", "Cost per Participant:", value = 100, min = 0,
step = 1),
            actionButton("calculate", "Calculate", class = "btn-success"),
            actionButton("refresh", "Refresh", class = "btn-primary")
          ),

          mainPanel(
            h3("Results"),
            div(class = "results-text",
                plotOutput("errorPlot", height = "500px")
            ),
            uiOutput("result")
          )
        ),

        hr(),
      ),

      div(class = "footer-copyright",
        "© 2025 SciPinion"
      )
  )
)

# Define server logic
server <- function(input, output, session) {
```

```
reactiveData <- reactiveValues(data = NULL)

# Calculate the number of repeats needed
calculateRepeats <- function(icc, rhoXW) {
  numerator <- rhoXW^2 * (1 - icc)
  denominator <- icc * (1 - rhoXW^2)
  m <- numerator / denominator
  ceiling_m <- ceiling(m)  # Ceiling to ensure m is an integer
  list(m = m, ceiling_m = ceiling_m)
}

observeEvent(input$calculate, {
  req(input$icc > 0 & input$rhoXW > 0)

  calculation <- calculateRepeats(input$icc, input$rhoXW)
  calculated_m <- calculation$ceiling_m
  output$result <- renderUI({
    HTML(paste(
      "<hr>",
      "<b>Calculated number of repeats needed:</b> ", calculated_m,
      "<br>",
      "<b>Exact value before ceiling:</b> ", round(calculation$m, 2)
    ))
  })
})
```

```
 observeEvent(input$refresh, {
  updateSliderInput(session, "icc", value = 0.3)
  updateSliderInput(session, "rhoXW", value = 0.85)
  output$result <- renderText({ "" })
 })

 output$errorPlot <- renderPlot({
  icc_values <- seq(0.05, 0.9, by = 0.05)
  rhoXW_value <- input$rhoXW
  m_values <- sapply(icc_values, function(icc) {
   calculateRepeats(icc, rhoXW_value)$ceiling_m
  })

  df <- data.frame(ICC = icc_values, m = m_values)

  p <- ggplot(df, aes(x = ICC, y = m)) +
   geom_line(color = "#0082CA") +
   theme_minimal() +
   labs(title = "Number of Repeats Needed for Desired Validity Coefficient",
      x = "Intraclass Correlation Coefficient (ICC)",
      y = "Number of Repeats (m)",
      subtitle = paste("Desired Validity Coefficient (rhoXW) =", rhoXW_value)) +
   geom_vline(xintercept = input$icc, linetype = "dashed", color = "#FF8228") +
   geom_hline(yintercept = calculateRepeats(input$icc, rhoXW_value)$ceiling_m, linetype
= "dashed", color = "#FF8228") +
   annotate("text", x = input$icc, y = calculateRepeats(input$icc, rhoXW_value)$ceiling_m,
```

```
            label = paste("m =", calculateRepeats(input$icc, rhoXW_value)$ceiling_m), color =
"#FF8228", hjust = -0.1, vjust = -1, size = 5) +
      theme(plot.title = element_text(hjust = 0.5, size = 20, color = "#0082CA"),
           axis.title = element_text(size = 16, color = "#515861"),
           axis.text = element_text(size = 16, color = "#515861"),
           axis.line = element_line(size = 1, colour = "#515861"),
           plot.caption = element_text(size = 14, hjust = 0, color = "#515861"),
           plot.subtitle = element_text(size = 16, color = "#515861"),
           legend.text = element_text(size = 14, color = "#515861"),
           legend.title = element_text(size = 14, color = "#515861"))

    p
  })

  div(class = "footer-copyright",
      "© 2025 SciPinion"
  )
}

# Run the application
shinyApp(ui = ui, server = server)
```

```
# Calculator Minimum Detectable Effect (MDE) Calculators for Linear Regression
# optimizing number of subjects (calculator LIN-N)
# https://scipinion.shinyapps.io/LinearRegressionN/
# 10.63565/scipinion.resource.lin-n

library(shiny)
library(shinyjs)
library(ggplot2)
library(pwr)

# Define UI
ui <- fluidPage(
  useShinyjs(),
  div(class = "container",
    div(class = "content",
      h1("Sample Size and MDE Tradeoff Calculation for Linear Regression with Classical Additive Measurement Error (LIN-N)"),
      p(class = "lead", "Calculate sample sizes considering measurement errors in linear regression"),

      sidebarLayout(
        sidebarPanel(
          numericInput("vy", "Variance of Y:", value = 1, min = 0.0001),
          numericInput("vw", "Within-Subject Variance (Vw):", value = 1, min = 0.0001),
          numericInput("m", "Number of measurements per subject (m):", value = 1, min = 1, step = 1),
          radioButtons("mode", "Select Input Mode:",
```

```
                    choices = list("Use ICC Slider" = "icc_slider", "Enter Vb and Vw" =
"var_components"),
                    selected = "icc_slider"),
          uiOutput("varInputUI"),
          sliderInput("alpha", "Significance Level (alpha):", min = 0.01, max = 0.1, value = 0.05),
          sliderInput("power", "Power (1-beta):", min = 0.8, max = 0.99, value = 0.9),
          numericInput("mdeInput", "Enter a Specific MDE Value (absolute):", value = 0.3, min
= 0.01, step = 0.01),
          actionButton("calculate", "Calculate", class = "btn btn-success"),
          checkboxInput("showHeatmap", "Show Heat Map of n_Z vs. MDE and m (around
selected values)", value = FALSE),
          HTML("<h4>Input Notes:</h4>
            <ul>
             <li>ICC (Intraclass Correlation Coefficient): Ratio of between-subject to total
variance.</li>
             <li>MDE: Minimum Detectable Effect, entered as an absolute value.</li>
            </ul>
          "),
        ),
        mainPanel(
          plotOutput("tradeoffPlot", height = "400px"),
          conditionalPanel(
            condition = "input.showHeatmap == true",
            plotOutput("heatmapPlot", height = "400px")
          ),
          textOutput("resultText"),
          verbatimTextOutput("nXOutput"),
```

```
            verbatimTextOutput("nZOutput"),

            verbatimTextOutput("rSquaredOutput"),

            HTML("
              <hr>
              <h4>Output Notes:</h4>
              <ul>
                <li>Heat Maps: Narrow ranges are used for focused exploration of user-selected
values.</li>
                <li>n_X: Sample size without measurement error</li>
                <li>n_Z: Sample size with measurement error</li>
              </ul>
            ")
          )
        ),


        hr(),
      ),


      div(class = "footer-copyright",
        "© 2025 SciPinion"
      )
  ),
)


# Define server logic
server <- function(input, output, session) {
```

```
 #Auto-click the Calculate button on startup
 observe({
  req(input$vy, input$vw, input$m, input$mode, input$alpha, input$power,
input$mdeInput)
  if(input$mode == "icc_slider") {
   req(input$icc)
  } else {
   req(input$vb)
  }
  shinyjs::click("calculate")
  updateCheckboxInput(session, "showHeatmap", value = TRUE)
 })
 # Dynamic UI for variance input mode
 output$varInputUI <- renderUI({
  if (input$mode == "icc_slider") {
   sliderInput("icc", "Intraclass Correlation Coefficient (ICC):", min = 0, max = 1, value = 0.5,
step = 0.01)
  } else {
   numericInput("vb", "Between-Subject Variance (Vb):", value = 1, min = 0.0001)
  }
 })


 # Calculate ICC based on mode
 reactive_icc <- reactive({
  if (input$mode == "icc_slider") {
   input$icc
  } else {
```

```
    input$vb / (input$vb + input$vw)  # Convert Vb to ICC for direct entry mode
  }
})

# Use reactive_icc for calculations
reactive_calculate <- eventReactive(input$calculate, {
  z_alpha_2 <- qnorm(1 - input$alpha / 2)
  z_beta <- qnorm(input$power)
  mde <- input$mdeInput
  Vy <- input$vy
  Vw <- input$vw
  m <- input$m
  Vb <- input$vw * reactive_icc() / (1 - reactive_icc())  # Compute Vb using ICC

  Vres <- Vy - mde^2 * Vb
  Sx <- sqrt(Vb)
  Sres <- sqrt(Vres)

  # Calculate standardized effect size
  d <- mde * Sx / Sres

  # Calculate n_X (sample size without measurement error)
  n_X <- ((z_beta + z_alpha_2) / d)^2

  # Calculate n_Z (sample size with measurement error)
  n_Z <- n_X / (Vb / (Vb + Vw / m))
```

```
  # Calculate r^2
  r_squared <- 1 - (Vres / Vy)

  list(n_X = n_X, n_Z = n_Z, r_squared = r_squared, mde = mde)
 })

 # Output the calculated n_X, n_Z, and r^2
 output$nXOutput <- renderText({
  calc <- reactive_calculate()
  sprintf("Calculated n_X (without measurement error) for MDE = %.2f: %.0f", calc$mde,
calc$n_X)
 })

 output$nZOutput <- renderText({
  calc <- reactive_calculate()
  sprintf("Calculated n_Z (with measurement error) for MDE = %.2f: %.0f", calc$mde,
calc$n_Z)
 })

 output$rSquaredOutput <- renderText({
  calc <- reactive_calculate()
  sprintf("Calculated r^2 for MDE = %.2f: %.4f", calc$mde, calc$r_squared)
 })

 # Render the plot and result text
 observeEvent(input$calculate, {
```

```
  mde_range <- seq(0.1, 1, by = 0.01)
  Vb <- input$vw * reactive_icc() / (1 - reactive_icc())  # Use calculated ICC

  sample_sizes <- sapply(mde_range, function(mde) {
   Vy <- input$vy
   Vw <- input$vw
   m <- input$m
   Vres <- Vy - mde^2 * Vb
   Sx <- sqrt(Vb)
   Sres <- sqrt(Vres)
   d <- mde * Sx / Sres
   n_X <- ((qnorm(1 - input$alpha / 2) + qnorm(input$power)) / d)^2
   n_Z <- n_X / (Vb / (Vb + Vw / m))
   r_squared <- 1 - (Vres / Vy)
   n_Z
  })

  plot_data <- data.frame(MDE = mde_range, SampleSize = sample_sizes)

  output$tradeoffPlot <- renderPlot({
   ggplot(plot_data, aes(x = MDE, y = SampleSize)) +
    geom_line(color = "blue") +
    geom_vline(xintercept = input$mdeInput, linetype = "dashed", color = "red") +
    geom_hline(yintercept = reactive_calculate()$n_Z, linetype = "dashed", color = "red") +
    geom_point(aes(x = input$mdeInput, y = reactive_calculate()$n_Z), color = "red", size =
3) +
```

```
      labs(title = "Sample Size vs. Minimum Detectable Effect Size",
           x = "Minimum Detectable Effect Size (MDE)",
           y = "Required Sample Size (n_Z)") +
      theme_minimal() +
      theme(plot.title = element_text(hjust = 0.5, size = 20),
            axis.title = element_text(size = 18),
            axis.text = element_text(size = 16),
            axis.line = element_line(color = "black"),
            panel.grid.major = element_line(color = "grey90"),
            panel.grid.minor = element_line(color = "grey98"),
            panel.background = element_blank())
  })

  output$resultText <- renderText({
    calc <- reactive_calculate()
    sprintf("With inputs Vy=%.2f, Vw=%.2f, m=%d, alpha=%.3f, and power=%.2f, the calculated sample sizes range from %.0f to %.0f for MDE values from %.2f to %.2f. Calculated r^2 for MDE = %.2f: %.4f",
            input$vy, input$vw, input$m, input$alpha, input$power,
            min(sample_sizes), max(sample_sizes), min(mde_range), max(mde_range), calc$mde, calc$r_squared)
  })
 })

 # Generate heat map for n_Z vs. mde and m if checkbox is selected
 output$heatmapPlot <- renderPlot({
  req(input$showHeatmap)  # Only render if checkbox is selected
```

```
# Define narrower ranges around user-selected values for mde and m, ensuring m >= 1
mde_range <- seq(input$mdeInput - 0.1, input$mdeInput + 0.1, by = 0.01)
m_range <- seq(max(1, input$m - 2), input$m + 2, by = 1)  # Ensures m is at least 1
Vb <- input$vw * reactive_icc() / (1 - reactive_icc())

heatmap_data <- expand.grid(MDE = mde_range, m = m_range)
heatmap_data$n_Z <- mapply(function(mde, m) {
  Vy <- input$vy
  Vw <- input$vw
  Vres <- Vy - mde^2 * Vb
  Sx <- sqrt(Vb)
  Sres <- sqrt(Vres)
  d <- mde * Sx / Sres
  n_X <- ((qnorm(1 - input$alpha / 2) + qnorm(input$power)) / d)^2
  n_Z <- n_X / (Vb / (Vb + Vw / m))
  n_Z
}, heatmap_data$MDE, heatmap_data$m)

# Generate heat map
ggplot(heatmap_data, aes(x = MDE, y = m, fill = n_Z)) +
  geom_tile() +
  scale_fill_viridis_c() +
  labs(title = "Heat Map of n_Z vs. MDE and m (Zoomed In)",
       x = "Minimum Detectable Effect Size (MDE)",
       y = "Number of Measurements per Subject (m)",
```

```
        fill = "Sample Size (n_Z)") +
    theme_minimal() +
    theme(plot.title = element_text(hjust = 0.5, size = 20),
          axis.title = element_text(size = 18),
          axis.text = element_text(size = 16))
  })

}



# Run the application
shinyApp(ui = ui, server = server)
```

```
# Calculator Minimum Detectable Effect (MDE) Calculators for Linear Regression

# optimizing number of samples per individual (calculator LIN-M)

# https://scipinion.shinyapps.io/LinearRegressionM/

# 10.63565/scipinion.resource.lin-m


library(shiny)

library(ggplot2)

library(DT)


# Define UI

ui <- fluidPage(

  div(class = "container",

      div(class = "content",

        h1("Linear regression M (LIN-M)"),

        p(class = "lead", "Calculate the relationship between number of measurements and minimum detectable effect size"),


        sidebarLayout(

          sidebarPanel(

            numericInput("vy", "Variance of Y:", value = 1, min = 0.0001),

            numericInput("vw", "Within-Subject Variance (Vw):", value = 1, min = 0.0001),

            numericInput("nZInput", "Enter a Specific n_Z Value:", value = 100, min = 1, step = 1),

            radioButtons("mode", "Select Input Mode:",

                         choices = list("Use ICC Slider" = "icc_slider", "Enter Vb and Vw" = "var_components"),

                         selected = "icc_slider"),

            uiOutput("varInputUI"),
```

```
      sliderInput("alpha", "Significance Level (alpha):", min = 0.01, max = 0.1, value = 0.05),

      sliderInput("power", "Power (1-beta):", min = 0.8, max = 0.99, value = 0.9),

      numericInput("selectedM", "Select m Value for Cross-Hair:", value = 10, min = 1, max
= 100, step = 1),

      actionButton("calculate", "Calculate", class = "btn-success")

     ),


     mainPanel(

      h3("Results"),

      plotOutput("tradeoffPlot", height = "400px"),

      textOutput("resultText"),

      verbatimTextOutput("mdeOutput")

     )

    ),


    hr(),

   ),


   div(class = "footer-copyright",

    "© 2025 SciPinion"

   )

  ),

)


# Define server logic

server <- function(input, output, session) {
```

```
output$varInputUI <- renderUI({
  if (input$mode == "icc_slider") {
    sliderInput("icc", "Intraclass Correlation Coefficient (ICC):", min = 0, max = 1, value = 0.5,
step = 0.01)
  } else {
    numericInput("vb", "Between-Subject Variance (Vb):", value = 1, min = 0.0001)
  }
})

# Reactive function to compute ICC
reactive_icc <- reactive({
  if (input$mode == "icc_slider") {
    input$icc
  } else {
    input$vb / (input$vb + input$vw)
  }
})

# Calculation logic for MDE and other parameters
reactive_calculate <- eventReactive(input$calculate, {
  z_alpha_2 <- qnorm(1 - input$alpha / 2)
  z_beta <- qnorm(input$power)
  n_Z <- input$nZInput
  Vy <- input$vy
  Vw <- input$vw
  Vb <- input$vw * reactive_icc() / (1 - reactive_icc())
```

```
  m_values <- 1:100

  mde_values <- sapply(m_values, function(m) {
    Vres <- Vy * (1 - Vb / (Vb + Vw / m))
    Sx <- sqrt(Vb)
    Sres <- sqrt(Vres)
    d <- (z_beta + z_alpha_2) / sqrt(n_Z * (Vb / (Vb + Vw / m)))
    mde <- d * Sres / Sx
    round(mde, 2)
  })

  data.frame(m = m_values, MDE = mde_values)
})

# Plot output
output$tradeoffPlot <- renderPlot({
  plot_data <- reactive_calculate()
  selected_m <- input$selectedM
  selected_mde <- plot_data[plot_data$m == selected_m, "MDE"]

  ggplot(plot_data, aes(x = m, y = MDE)) +
    geom_line(color = "#0082CA") +
    geom_vline(xintercept = selected_m, linetype = "dashed", color = "#FF8228") +
    geom_hline(yintercept = selected_mde, linetype = "dashed", color = "#FF8228") +
    geom_point(aes(x = selected_m, y = selected_mde), color = "#FF8228", size = 3) +
```

```
    labs(title = "Number of Measurements vs. Minimum Detectable Effect Size",
         x = "Number of Measurements per Subject (m)",
         y = "Minimum Detectable Effect Size (MDE)") +
    theme_minimal() +
    theme(plot.title = element_text(hjust = 0.5, size = 20, color = "#0082CA"),
          axis.title = element_text(size = 18, color = "#515861"),
          axis.text = element_text(size = 16, color = "#515861"),
          axis.line = element_line(color = "#515861"),
          panel.grid.major = element_line(color = "#bac4cf"),
          panel.grid.minor = element_line(color = "#edf2f9"),
          panel.background = element_blank())
  })

  # Text result output
  output$resultText <- renderText({
    sprintf("With inputs Vy = %.2f, Vw = %.2f, alpha = %.3f, power = %.2f, and n_Z = %d, the calculated MDE values are displayed across all integer m values from 1 to 100. The red cross-hairs show the intersection point for the selected m value.",
          input$vy, input$vw, input$alpha, input$power, input$nZInput)
  })

  # Verbatim MDE output for each m value
  output$mdeOutput <- renderText({
    plot_data <- reactive_calculate()
    mde_summary <- paste(sprintf("m = %d: MDE = %.2f", plot_data$m, plot_data$MDE), collapse = "\n")
    paste("Calculated MDE values across different m values:\n", mde_summary)
```

```
  })

}

# Run the application
shinyApp(ui = ui, server = server)
```

```
# Calculator Power and Bias Calculator for Logistic Regression  (calculator LOGIT-PB)
# https://scipinion.shinyapps.io/SensitivityAnalysisExplorer/
# 10.63565/scipinion.resource.logit-pb
# The simulation data can be downloaded (simdata.csv file), which is required to run the
# application, by visiting the DOI and clicking the "Download Simulation Data (CSV)" button
# at the very bottom of the page.

library(shiny)
library(ggplot2)
library(shinycssloaders)
library(shinyjs)
library(DT)

# Load the simulation results
results_df <- read.csv("simdata.csv")

# Define UI
ui <- fluidPage(
  useShinyjs(),
  div(class = "container",
      div(class = "content",
          h1("Simulation of Measurement Error Effects in Logistic Regression (LOGIT-PB)"),
          p(class = "lead", "Explore power and bias in simulated data"),

          fluidRow(
            column(4,
```

```
div(class = "control-panel",
    numericInput("OR", "True Odds Ratio (OR):", value = 1.1, min = 0.1, step = 0.1),
    numericInput("pr_0", "Background Probability (pr_0):", value = 0.1, min = 0.01,
max = 0.99, step = 0.01),
    numericInput("Vb", "Between-Person Variance (Vb):", value = 6, min = 0.1, step =
0.1),
    numericInput("Vw", "Within-Person Variance (Vw):", value = 3, min = 0.1, step =
0.1),
    numericInput("mux", "Mean of True Exposure (mux):", value = 1.3, step = 0.1),
    numericInput("sample_size", "Population Sample Size (n):", value = 800, min =
10, step = 10),
    numericInput("m", "Number of Measurements per Person (m):", value = 2, min =
1, step = 1),
    numericInput("sim_size", "Number of Simulations:", value = 1000, min = 100,
step = 100),
    actionButton("runSim", "Run Simulation", class = "btn btn-success")
  )
),
column(8,
  conditionalPanel(
    condition = "output.simulationRun",
    withSpinner(
      div(
        textOutput("powerOutput"),
        textOutput("biasOutput"),
        DTOutput("quantilesTable"),
        plotOutput("ORHistPlot"),
        plotOutput("biasDistPlot")
```

```
              )
             )
            )
        )
       ),

       hr(),
     ),

     div(class = "footer-copyright",
       "© 2025 SciPinion"
     )
  )
)

server <- function(input, output, session) {
  # Track if simulation has been run
  simStatus <- reactiveVal(FALSE)

  # Auto-click the Run Simulation button on startup
  observe({
    shinyjs::click("runSim")
  })

  observeEvent(input$runSim, {
    set.seed(58471313)
```

```
sim_betax <- numeric(input$sim_size)
sim_betaxse <- numeric(input$sim_size)
sim_betaxp <- numeric(input$sim_size)

effect <- log(input$OR)
intercept <- log(input$pr_0 / (1 - input$pr_0))
var_ex <- input$Vw / input$m

withProgress(message = 'Running simulations...', value = 0, {
 for (i in 1:input$sim_size) {
  x <- rnorm(input$sample_size, input$mux, sqrt(input$Vb))
  ex <- rnorm(input$sample_size) * sqrt(var_ex)
  xx <- x + ex

  y <- numeric(input$sample_size)
  for (j in 1:input$sample_size) {
   prob <- exp(intercept + effect * x[j]) / (1 + exp(intercept + effect * x[j]))
   y[j] <- rbinom(1, 1, prob)
  }

  reg <- glm(y ~ xx, family = binomial)
  se <- sqrt(diag(vcov(reg)))
  sim_betax[i] <- reg$coefficients[2]
  sim_betaxse[i] <- se[2]
  sim_betaxp[i] <- 2 * (1 - pnorm(abs(sim_betax[i] / sim_betaxse[i])))
```

```
    incProgress(1 / input$sim_size)
  }
})

P_TEST <- 0.05
power <- mean(sim_betaxp < P_TEST) * 100
pct_bias <- 100 * (exp(sim_betax) - exp(effect)) / exp(effect)

OR_quantiles <- quantile(exp(sim_betax), c(0.5, 0.025, 0.975))
bias_quantiles <- quantile(pct_bias, c(0.5, 0.025, 0.975))

# Outputs
output$powerOutput <- renderText({
  sprintf("Power (%% of significant results at p = %.2f): %.2f%%", P_TEST, power)
})

output$biasOutput <- renderText({
  sprintf("Mean Bias (%%): %.2f", mean(pct_bias))
})

output$quantilesTable <- renderDT({
  data.frame(
    Metric = c("Odds Ratio (50%, 2.5%, 97.5%)", "Bias (50%, 2.5%, 97.5%)"),
    "50%" = round(c(OR_quantiles[1], bias_quantiles[1]), 2),
    "2.5%" = round(c(OR_quantiles[2], bias_quantiles[2]), 2),
    "97.5%" = round(c(OR_quantiles[3], bias_quantiles[3]), 2)
```

```
    )
  }, options = list(dom = 't', paging = FALSE))

  output$ORHistPlot <- renderPlot({
    ggplot(data.frame(logOR = exp(sim_betax)), aes(x = logOR)) +
      geom_histogram(aes(y = ..density..), bins = 30, fill = "blue", color = "black", alpha = 0.7)
+
      geom_density(color = "chocolate", lwd = 1.5) +
      labs(title = "Histogram of Simulated Odds Ratios", x = "Odds Ratio", y = "Density") +
      theme_minimal() +
      theme(
        plot.title = element_text(size = 18, face = "bold", hjust = 0.5),
        axis.title = element_text(size = 16),
        axis.text = element_text(size = 14)
      )
  })

  output$biasDistPlot <- renderPlot({
    ggplot(data.frame(pct_bias = pct_bias), aes(x = pct_bias)) +
      geom_histogram(aes(y = ..density..), bins = 30, fill = "red", color = "black", alpha = 0.7) +
      geom_density(color = "chocolate", lwd = 1.5) +
      labs(title = "Bias Distribution", x = "Percentage Bias", y = "Density") +
      theme_minimal() +
      theme(
        plot.title = element_text(size = 18, face = "bold", hjust = 0.5),
        axis.title = element_text(size = 16),
```

```
      axis.text = element_text(size = 14)
    )
  })

  # Mark simulation as run
  simStatus(TRUE)
 })

 # Control visibility of outputs
 output$simulationRun <- reactive({
  simStatus()
 })
 outputOptions(output, "simulationRun", suspendWhenHidden = FALSE)



}

shinyApp(ui = ui, server = server)
```
