# Supplementary material for: Improving the design of epidemiology studies that use biomonitoring for exposure assessment: a SciPinion panel recommendation
Source: BMC Med Res Methodol. 2026 Jan 12;26:29. doi: 10.1186/s12874-025-02753-5 (PMC12888676; doi:10.1186/s12874-025-02753-5)
Supplement: Supplementary file 3 — Additional file 3. Full report from the SciPinion panel of experts. [file 12874_2025_2753_MOESM3_ESM.pdf]

Appendix C: Expert Panel Engagement

SciPinion engaged an independent panel of experts to serve on a science advisory panel (SAP) using methods described in Kirman et al. (2019). The process was designed with the goal of maximizing the pool of ideal panelists, defined as the intersection of four populations, people who have expertise in the subject matter, are objective, are available to participate, and are willing to participate. Seven experts in human health exposure, toxicity, and risk assessment were identified to participate in this panel. The process for recruiting, selecting, and engaging the expert panel is described below.

Panel Recruitment

Potential candidates were identified as having relevant experience in epidemiology, statistics, biomonitoring and power/bias using a variety of sources, including: (1) SciPinion's internal database; (2) searches for authors of recent publications on the topic of interest in online databases (e.g., Pubmed, Google Scholar); (3) searches of profiles on social media databases (e.g., LinkedIn); (4) general internet searches; and (5) referrals. Email addresses were obtained for as many potential candidates as possible. An email invitation was sent to all potential candidates, requesting interested candidates to volunteer on https://app.scipinion.com, upload a copy of their CV, and provide a brief application statement (*i.e.*, *what makes you qualified for this panel?*). SciPinion received CVs from a total of 258 applicants, 16 of which were excluded for failing to upload their CV, leaving 242 candidates to go through the next step of the process.

Panel Selection

A triple blinded process was used: (1) candidates were blinded to the review sponsor; (2) the review sponsor was blinded to the candidates and played no role in selection; and (3) those selected for the panel were blinded to one another. Expertise data provided by the applicants and extracted from their CVs were used to rank the candidates with respect to general expertise metrics (e.g., academic degree, number of years of experience, number of publications) and topic-specific expertise metrics (e.g., CV key word counts).

Nine panel members were selected by SciPinion and an independent auditor (coauthor IB) from the available candidates based upon the expertise metrics described above. Additional candidates were identified as potential alternates, in case a panelist is unable to complete the participation.

Panel Engagement

The 9 panel members were placed under contract. Email addresses corresponding to their SciPinion user accounts were verified as belonging to the experts (i.e., associated with their publication record, with their place of employment, or verified by personal

communication). Charge questions were developed by SciPinion and coauthors IB and TC.

During the application process and throughout the peer review, panel members were blinded to the identities of their fellow panel members (identified online only by their display names of "Expert 1", "Expert 2"...). Individual responses to the charge questions are linked to the experts anonymized display names, and not to their identities, an effort intended to provide psychological safety.

The primary review material consisted of a white paper prepared by SciPinion (Appendix D), access to the calculators via online hosting, and the following select publications from the published literature:

- Armstrong 1996
- Fleiss 1986
- Preau and Calafat 2010

Panel members were also permitted to request additional publications and reports as needed to support their participation. The expert panel engagement was structured to have 3 rounds using a modified Delphi format (start in April of 2024, completion in June of 2024):

- *Round 1* – Panel members worked independently to read the review material (Appendix D; select publications and reports) and answer Round 1 charge questions. All 9 panel members completed their assignment as scheduled.
- *Round 2* – Panel members worked deliberatively to review and comment on each other's responses to Round 1 questions. All participation was conducted online (app.scipinion.com) in an anonymous manner (i.e., experts were randomly assigned display names "Expert 1", "Expert 2"...).
- *Round 3* – Panel members worked independently to review additional charge questions. All panel members completed this round as scheduled.

All charge questions and panel member responses from this engagement are provided below.

SciPinion

SURVEY RESULTS

Improving Epidemiology Study Designs When Using Biomonitoring for Exposure Assessment

SciPinion is seeking a panel of experts that will review statistical calculators for power and bias and develop recommendations to guide epidemiology study designs when biomonitoring is used for exposure assessment

Generated: 2024-10-01 02:34:34 +0000
URL: https://app.scipinion.com/scipis/646/report

BACKGROUND

Result 1.1 *(ID: 6537)*
Question 1.1 *(ID: 5889)*

In your experience, how often do researchers conducting epidemiology studies using biomonitoring data for exposure assessments consider the variability (between- and within-person) in the exposure metric?

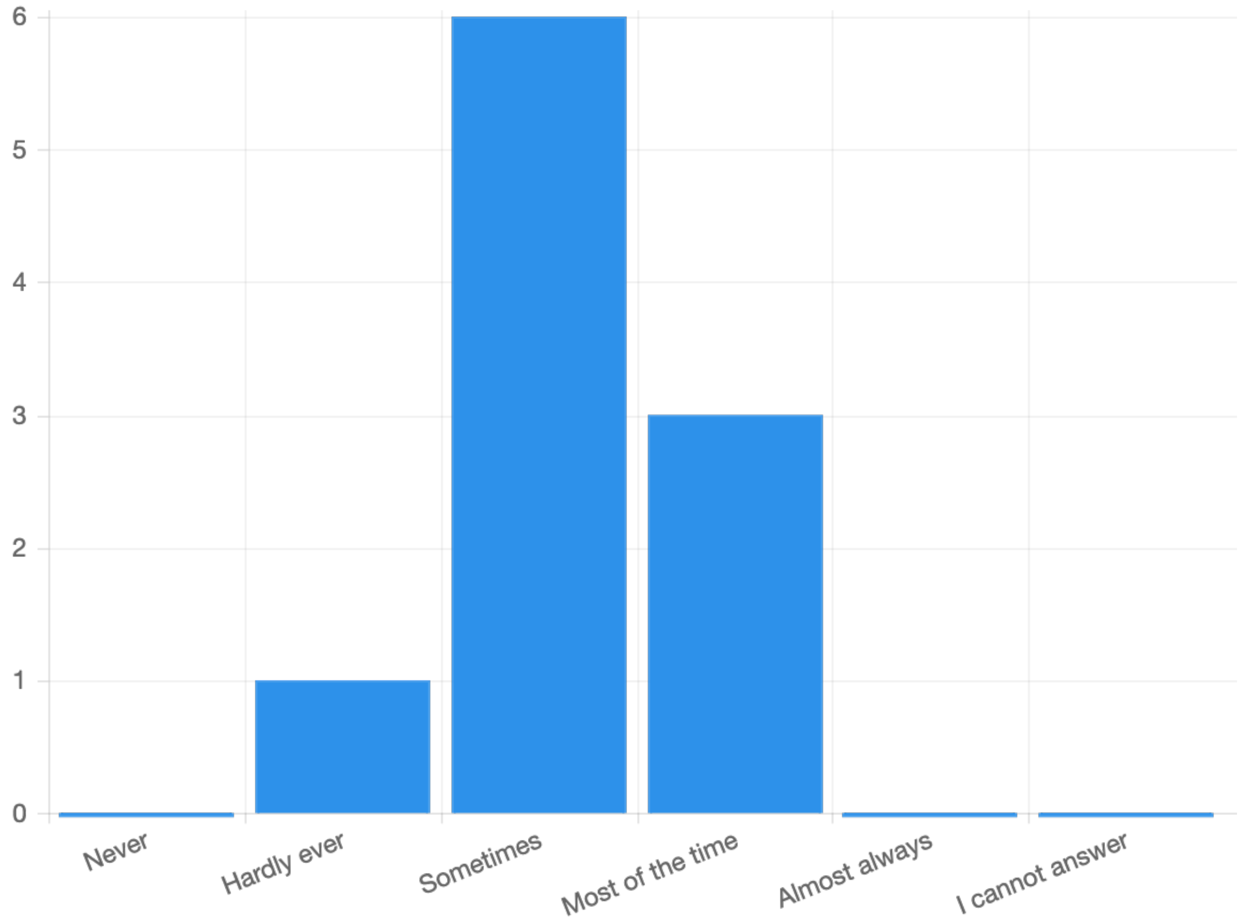

Legend

answers: 9
skips: 0

Answer Explanations

Expert 3 Explanation
Selected Answer(s): Hardly ever Sometimes

In my research projects, my collaborators consistently consider both between- and within-person exposure variability. However, I recognize that this group is somewhat unique in this regard, as they were among the pioneers in developing these concepts (Kromhout, Vermeulen, Heederik, Brunekreef, Burstyn). Outside of my group, I have encountered only one research team from Finland that also accounted for between- and within-person variability. To date, I have personally not come across a U.S.-based health research group led by MDs that

has incorporated this consideration. I have only reviewed one article that addressed between- and within-person variability, although this is emphasized in the leading textbook (Quantitative Exposure Assessment by Lawrence Kupper and Steven Rappaport) on quantitative exposure assessment.

Expert 4 Explanation
Selected Answer(s): Sometimes

Depends on data availability, if multiple measurements are available most researchers will use all of them, and sometimes may even exclude participants with too few measurements.

Expert 2 Explanation
Selected Answer(s): Most of the time

The consideration of variability (both between-person and within-person) in exposure metrics has become increasingly common in epidemiology studies using biomonitoring data. Over the past two decades, the importance of accounting for variability has been widely recognized, driven by the understanding that ignoring it can lead to exposure misclassification and biased results.

Many studies now incorporate designs and statistical methods that address variability, such as repeated measures and mixed-effects models. This shows that researchers are mindful of variability in their assessments. Additionally, the trend towards following guidelines and best practices (such as WHO's Biological Monitoring of Chemical Exposure in the Workplace, EFSA's Human biomonitoring data collection from occupational exposure to pesticides) has led to more consistent reporting on how variability is handled, especially in studies published in reputable journals.

However, there are still instances where variability might not be fully considered, particularly in studies with limited resources or less experienced researchers. In these cases, variability may be acknowledged but not thoroughly addressed, or it might be overlooked due to practical constraints.

The frequency of considering variability also varies depending on the study context, such as the population being studied and the nature of the exposure. Occupational studies, might be more rigorous in this regard compared to some environmental studies where repeated measures are harder to obtain.

In summary, addressing variability is often practiced but not yet universally guaranteed. The field has made significant progress, but there is still room for improvement in ensuring that variability is consistently and thoroughly considered across all contexts.

Expert 6 Explanation
Selected Answer(s): Sometimes

This emphasis make this paper a useful contribution.

Expert 7 Explanation
Selected Answer(s): Sometimes

There is an increasing recognition of the importance of considering both between- and within-person variability in epidemiology studies using biomonitoring data. However, cost considerations often require choosing between enrolling more subjects vs making more measurments. Prior knowledge of between- and within-person variability is needed for best study design but is rarely available.

Expert 1 Explanation

Selected Answer(s): Sometimes

There are obviously numerous factors to consider, these include sample size, timing of biomonitoring measurements (hourly, daily, weekly, etc...), assessment of confounding factors (e.g., diet), purpose of biomonitoring (e.g., athletes for drug assessment), and the type of testing method used including type of sample used (e.g., urine, sweat, tears, etc...). Studies sometimes assess both between and within variability, but this is not always the case.

Expert 9 Explanation

Selected Answer(s): Most of the time

Most researchers I know consider all likely sources of variability (both between- and within-person) in designing studies or critiquing other studies. They might not do so explicitly in sample size calculations, but might consider it implicitly for example, by increasing overall sample size to account (partially) for within-person variation, or by other approaches to decrease within-person variation (such as taking 24-hour voids or first morning void for urine specimens).

Expert 8 Explanation

Selected Answer(s): Sometimes

Often there are some measures of between- and within-person variability described, but not always. I would not say 'most of the time'. Between person is more often reported than within person. Also depends on the study design, e.g. whether longitudinal data are available.

Expert 5 Explanation

Selected Answer(s): Most of the time

Overall, I think that most epidemiologists don't often consider the "within" variation for individuals, but almost always consider the between variation as that measure is necessary to know (or estimate) for basic sample size calculations.

Those epidemiologists who work primarily on studies using biomonitoring are well aware of the within-individual variation and the effects of this and repeated measurements on exposure measurement error, study power, and bias in risk estimates.

Generally, we don't have good estimates (from the field) of either within or between variation to assist with sample size/power estimations.

Comments (7)

SCORE 0 — **Expert 6** — *08/31/2024 11:39*

Why is the number of answers (and number of experts) = 9, while the graph shows 10 responses?

SCORE 1 — **Expert 9** — *08/31/2024 13:25*

In response to Expert 6: It looks like Expert 3 gave two responses.

Regarding Question 1.1: I suspect the variability in our responses might be due at least partly to different interpretations of the broad phrasing of the question. For example, I chose to interpret "consider" generally as "think about" and not rigorously as "include directly when doing sample size calculations". Furthermore and as others have mentioned, I believe most epidemiologists use between-person variability in sample size calculations as it is usually required. Within-person variability seems to be less commonly included in those calculations in my experience. And consideration of both types of variability is limited by factors such as availability of data, and/or not be described in detail in the Methods section of published articles.

SCORE
5
Expert 2
09/03/2024 07:15

Although I recognize the awareness and application of statistical methods to account for between- and within-person variability in epidemiology studies, as Expert 3 points out, the application of such practices is far from universal. Often, these practices are restricted to well-resourced groups or those with specific expertise. This raises concerns about the overall reliability of epidemiological findings, especially those from under-resourced settings or non-expert groups. Moreover, the practical challenges of incorporating such variability—highlighted by the need for extensive measurements and additional resources—suggest that its regular consideration might be more idealistic than realistic for many studies. This underlines a significant divide: while the theoretical importance of considering variability is clear, the actual practice is hindered by logistical and educational barriers. This situation underscores the need for more standardized practices, better resource allocation, and accessible tools in the epidemiological community.

SCORE
3
Expert 3
09/08/2024 14:52

Expert 2 has well summarized the issues: actual practice is hindered by logistical and educational barriers. Expert 5 has pointed out an interesting aspect that I have observed but had not considered before—the "within" variation is not as commonly addressed as between-subject variation. I would add that I have seen this both among epidemiologists and toxicologists. It is more common to have more subjects than to take repeated measurements. I wonder if there is another barrier, in addition to education, logistics, and finances, such as cognitive bias or a preference for simpler study designs.

SCORE
2
Expert 1
09/09/2024 00:47

The process of conducting biomonitoring studies with adequate Power (sample size) and repeated measures is a balancing act. Usually there are trade offs (i.e., larger sample size and less repeated measures or smaller sample size and more repeated measures). In addition, the application area (e.g., Forensic, Clinical, Sports Medicine, Environmental) will also affect these factors and the

metabolite/chemical being measured and the technique plays a role. I agree with Expert 2 that the within variation is not usually addressed but again this depends on the purpose for biomonitoring. If I was measuring a chemical/motabolite in an athlete within variation would be critical over time.

SCORE
1
Expert 5
09/11/2024 11:18

It seems from the various expert comments, that the consideration of within-subject variation is very dependent on the application area (Expert 1), the expertise of the research group (and here I would say that the Europeans are more advanced in their approach), and the background of the Principal Scientists initiating the work. I personally find that individuals trained in exposure assessment/science or industrial hygiene, and those who have real-world field experience collecting exposure (air, dermal etc.) and biological monitoring samples will consider all of the possible sources of variation (including variation in chemical analysis results) that could affect epidemiological study power and risk estimates.

SCORE
1
Expert 8
09/12/2024 13:28

Good discussion, I think many points have been addressed already. Some notes from my side:
-Part of the discrepancy in our original responses indeed seems to stem from a more semantic issue, from different interpretations of 'consider'. Maybe we can clarify this in the next round to improve our discussion.
-Expert 3 noted a difference in approach between European and US-based research groups. It would be interesting to explore this further, e.g.; is there a difference in regional guidelines or established practices? Or is this disparity more field-specific; are European groups more active in specific fields where variability consideration is more common? In general, this suggests a need for more collaboration and harmonization of approaches.
-Expert 1 and others already mentioned the crucial trade-offs between sample size and repeated measures. This balance is a key topic in this discussion, as resources are always constraint.

Result 1.2 *(ID: 6538)*
Question 1.2 *(ID: 5890)*

In your experience, how often do researchers conducting epidemiology studies using biomonitoring data for exposure assessments consider the half-life of the biomarker?

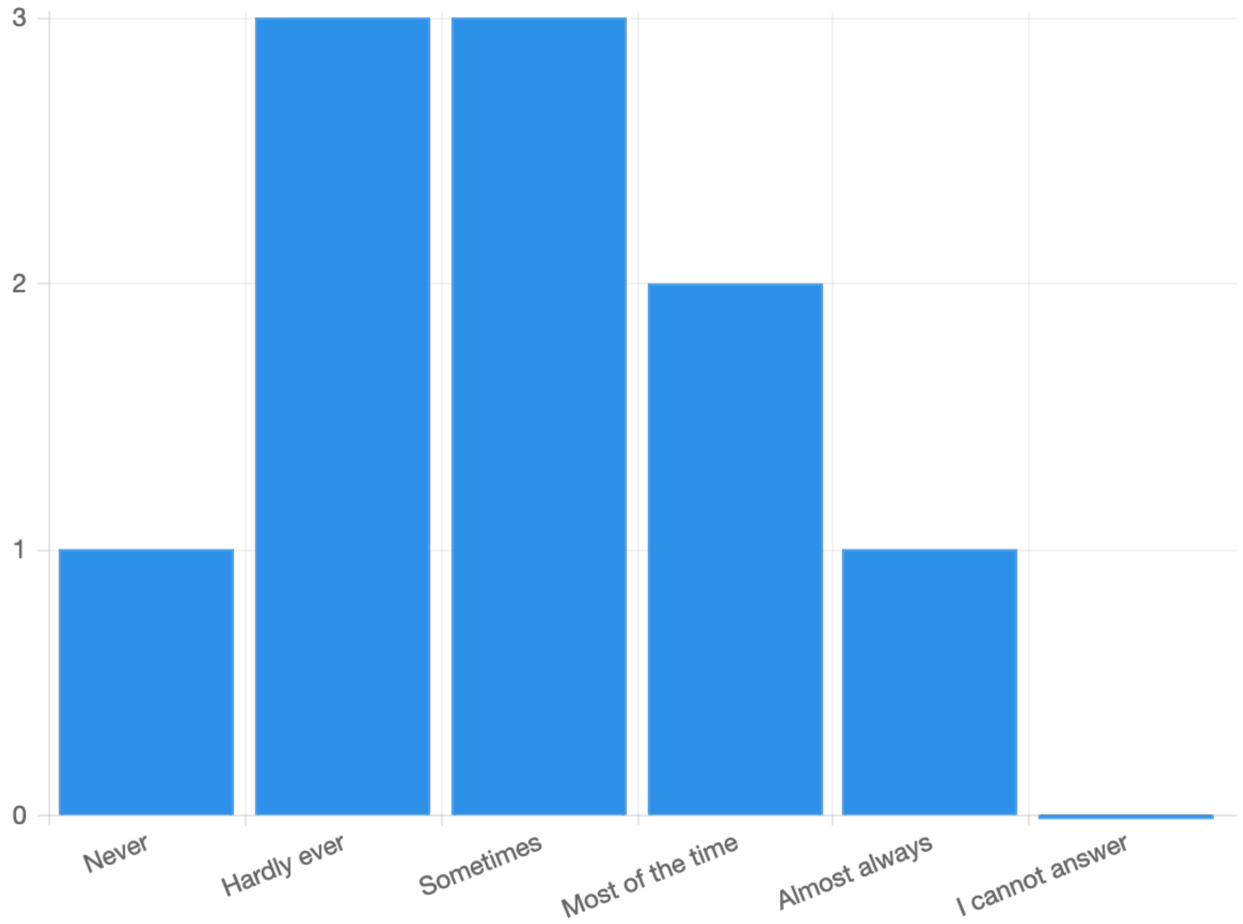

Legend

answers: 9
skips: 0

Answer Explanations

Expert 3 Explanation
Selected Answer(s): Never Hardly ever

I have not encountered an epidemiologist who considers the half-life of the biomarkers they use. However, my exposure assessment collaborators do take half-lives into account in their studies.

Expert 5 Explanation
Selected Answer(s): Most of the time

To be competitive for grant funding etc., a proposal that did not consider the half-life of a chemical in the biological monitoring plan would unlikely be funded. Although the sample collection procedures may not be ideal (i.e. single spot samples for short lived chemicals) for an epidemiologic study, the investigators should none the less be aware

of the limitations of their methods and draw appropriate conclusions.

Expert 4 Explanation
Selected Answer(s): Hardly ever
Very seldom, most researchers simply used what is available in the data and did not consider the half-life.

Expert 2 Explanation
Selected Answer(s): Sometimes
This is because while the half-life of a biomarker is a critical factor in accurately interpreting biomonitoring data, it is not always fully accounted for in epidemiological studies. Some studies, especially those with more experienced researchers or where the biomarker's half-life is well understood, do consider it. However, in many cases, this factor might be overlooked or not adequately addressed, particularly in studies with limited resources or a focus on short-term exposure metrics.

Expert 6 Explanation
Selected Answer(s): Hardly ever
Metabolism and excretion of the biomarker contribute to within-person variability but are not directly addressed in the paper.

Expert 7 Explanation
Selected Answer(s): Most of the time
Biomarkers with longer half-lives (e.g., hair, toenails) reduce the impact of short-term fluctuations and better estimate chronic exposure.
For exposures with known short half-lives, within-person variability is more often a critical factor.

Expert 1 Explanation
Selected Answer(s): Sometimes
The half-life of the biomarker is a critical component to undertaking exposure assessments in environmental epidemiology. However, not all studies use or determine the half-life when conducting biomonitoring studies. In addition, the analytical methods may differ between studies which makes comparisons of studies difficult. Not assessing the half-life would be a poor quality indicator.

Expert 9 Explanation
Selected Answer(s): Almost always
This is a fundamental characteristic of the biomarker; I have trouble imagining any researcher ignoring it. True, there might be little information in the literature about the half-life, and then the researcher might need to consider other options.

Expert 8 Explanation
Selected Answer(s): Sometimes
Similarly, I would say sometimes, not most of the time. Also strongly depends on the field and type of biomarker. I think in e.g. toxicology, pharmacology, it's more common, but there are various fields using biomarkers and it's not common to report/well known that it is important to all researchers. Sometimes factors like storage time, storage environment, or time between collection and analysis are taken into account in models.

Comments (6)

SCORE **Expert 6** 09/02/2024 09:34

1 I strongly agree with Expert 7's comment about biomarkers of longer-term exposure.

SCORE **Expert 2** 09/03/2024 07:25

2 The experts' opinions clearly diverge, highlighting variations in disciplinary focus and the specific requirements of different subfields. For example, as Expert 8 points out, those in toxicology and pharmacology are more likely to consider the half-life of biomarkers due to the direct relevance of pharmacokinetics in their work. In contrast, researchers in broader fields such as environmental epidemiology may not prioritize or possess the necessary expertise to incorporate these specific pharmacokinetic considerations into their studies.

SCORE **Expert 9** 09/03/2024 15:55

0 In consideration of the thoughtful comments by my colleagues here, I believe I interpreted the word "consider" too broadly, and will revise my response downward in Round 3.

SCORE **Expert 3** 09/08/2024 15:06

3 Expert 8 has very thoughtfully identified the issue. It seems to me that the expert opinions on this panel may be categorized into two groups: Group A, consisting of toxicologists and pharmacologists who actively work with biomarkers and consider half-lives essential in their research, and Group B, comprising epidemiologists who may not work with half-lives as regularly. Maybe Group B may be more accustomed to working with biomarkers that have longer half-lives, which could simplify the statistical analysis in epidemiological studies, even if the concept of half-life is not explicitly addressed.

SCORE **Expert 5** 09/11/2024 11:27

1 I look at this issue from both sides of the fence - I'm an epidemiologist who also was trained/conducted research in exposure assessment, biological monitoring and toxicology. Early in my career I realized that most PhD level epidemiologists had little understanding/training in exposure assessment/biological monitoring. The only discipline at that time that took these issues into consideration (and more specifically biological half-lives) was in nutritional epidemiology.

Now, we include lectures on these issues (toxicokinetics/toxicodynamics etc.) in graduate level courses generally in occupational/environmental/dietary epidemiology or exposure measurement, but I believe that the majority of epidemiologists "out there" have minimal understanding.

SCORE
1

Expert 8

09/12/2024 14:36

- There indeed seems to be a significant divide in how half-life is considered. I like expert 3's observation about categorizing responses into toxicology/pharmacology versus epidemiology. However, given that many epidemiologists have backgrounds in other health-related fields (which could include toxicology), and e.g. toxicologists apply epidemiological methods, hopefully the divide is not as stark as it might appear.
- As with our previous question, it would be useful to specify/clarify what we mean by 'consider' for our further discussion.
-Expert 7 provides an interesting comment how the half-life (e.g. shorter versus longer) is important in determining how important within-person variability is. It would be good to take this into account in our discussions.
- While direct comparisons are unlikely, I'm curious if anyone can share experiences where consideration of half-life significantly influenced study design, results or conclusions vs studies that did not? E.g. re-analyses of existing data or datasets that were enriched with additional measurements?

Result 1.3 *(ID: 6539)*
Question 1.3 *(ID: 5891)*

How would you characterize your familiarity with the magnitudes of between- and within-person variability in exposure biomarkers?

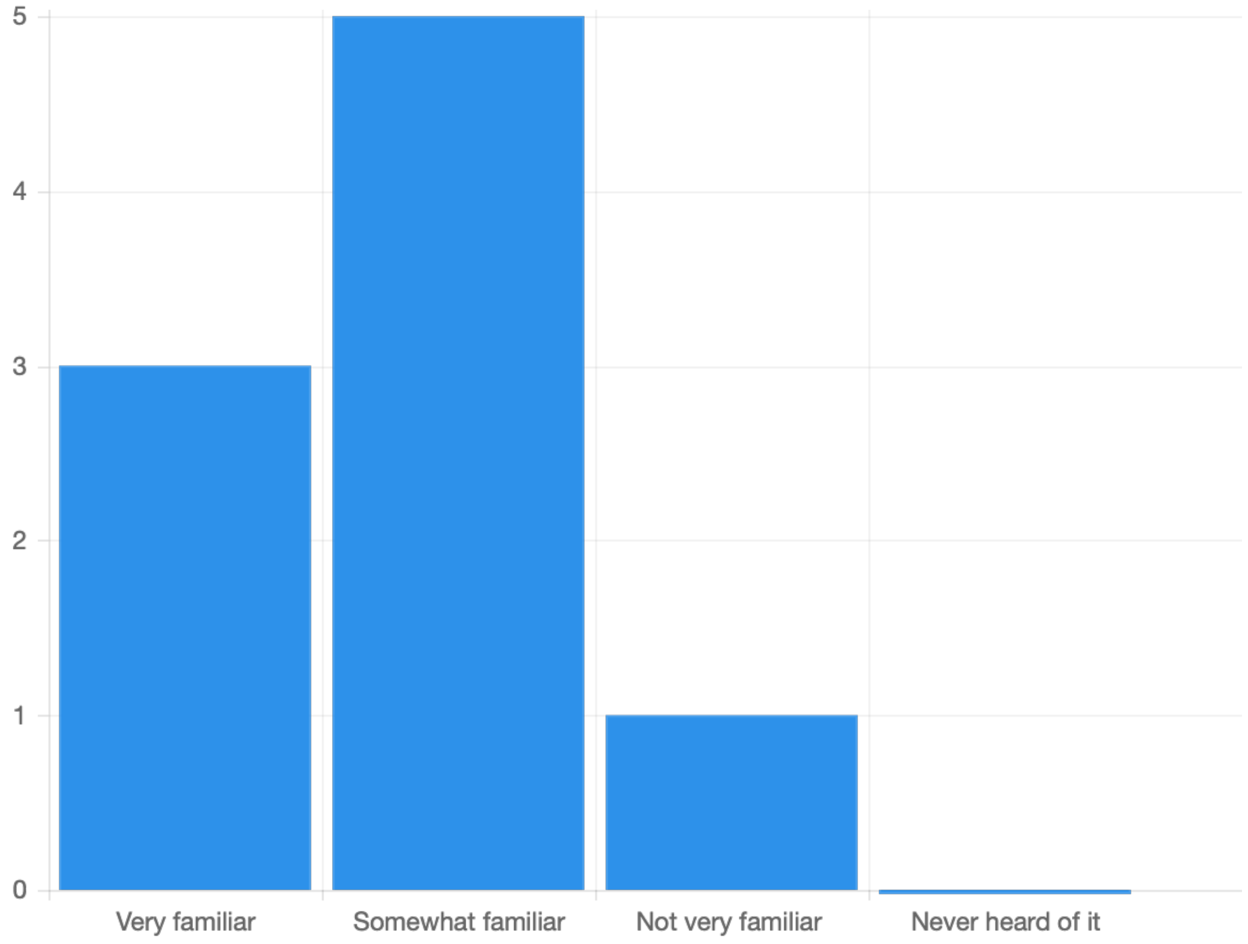

Legend

answers: 9
skips: 0

Answer Explanations

Expert 8 Explanation
Selected Answer(s): Somewhat familiar

I've worked with various biomarkers, but mostly limited to nutritional exposure assessment

Expert 3 Explanation
Selected Answer(s): Somewhat familiar

I am familiar with the magnitudes of between- and within-person variability for a few selected exposures, such as manganese, TCE, HCHO, and PAHs.

Expert 5 Explanation
Selected Answer(s): Very familiar

I've taught courses in exposure measurement error and effects on power and bias etc. in PhD level and other groups. I've studied this in field settings (pesticide exposure) using urine samples (spot samples, 24 hour etc.), and also studied the effects of urine dilution correction (SG and creatinine).

Expert 4 Explanation
Selected Answer(s): Very familiar

Have been working in a very similar topic for several years, which is about measurements of habitual physical activity level and sleep.

Expert 2 Explanation
Selected Answer(s): Somewhat familiar

I have been responsible for or involved in numerous environmental epidemiology studies focused on environmental risk factors, including heavy metals, pesticides, and air pollutants. Some of these studies utilized longitudinal designs with repeated measurements of exposures. In these studies, we employed advanced statistical techniques, such as mixed-effects models and Generalized Estimating Equations (GEE), to account for both between- and within-person variability as well as clustering effects in exposure data.

Expert 6 Explanation
Selected Answer(s): Very familiar

I am more familiar with clustering (within-person correlation) of outcomes in cluster-randomized trials and with clustered measurements due to small numbers of observers who measure the outcomes, but the statistical principles are the same.

Expert 7 Explanation
Selected Answer(s): Somewhat familiar

Non-persistent chemicals generally exhibit higher within-person variability, while persistent chemicals tend to show greater between-person variability.

Expert 1 Explanation
Selected Answer(s): Somewhat familiar

Variability of between person and within person is commonly measured in many types of epidemiological studies, including meta-analysis which compares the between study and within study variability. The magnitude of the variability is important in determining the the stability of the biomarker being measured over time.

Expert 9 Explanation
Selected Answer(s): Not very familiar

I'm familiar with the concept, but not the actual values of between- and within-person variability in exposure biomarkers. Plus of course, the actual values would vary between different exposure compounds, their half-lives and metabolic products, and sampling media (e.g. blood, urine, saliva).

Comments (3)

SCORE 3 **Expert 3** *09/08/2024 15:27*

The experts' answers on this panel demonstrate a strong understanding of between- and within-person variability, as well as the advanced statistical analyses used to assess these

variabilities. Based on the comments, it seems that some experts may not focus as much on the exact numerical values (e.g., Expert 9). Perhaps the question was somewhat ambiguous, and experts may not have explicitly addressed it. In my work, there also seems to be a greater emphasis on the ratio of between- to within-person variability rather than the absolute values. The ratio is often more important than the absolute values—e.g., when within-person variability exceeds between-person variability, it suggests the need for more repeats instead of more subjects. This could be similar to recognizing the significance of a study without necessarily recalling the exact p-value.

SCORE
0
Expert 5
09/11/2024 11:30
Expert 3 makes a good point about the ratio of between/within variation (generally the ICC-intraclass correlation).

SCORE
1
Expert 8
09/12/2024 14:42
It seems the question was interpreted somewhat differently by the different experts. Rereading it, I would also interpret it as referring to numerical magnitudes of variability. But expert 3 also makes an interesting point that ratios may be more important than absolute values.

Result 1.4 *(ID: 6540)*
Question 1.4 *(ID: 5892)*

How would you characterize your familiarity with the inter-class correlation coefficient (ICC) within the context of exposure biomarkers?

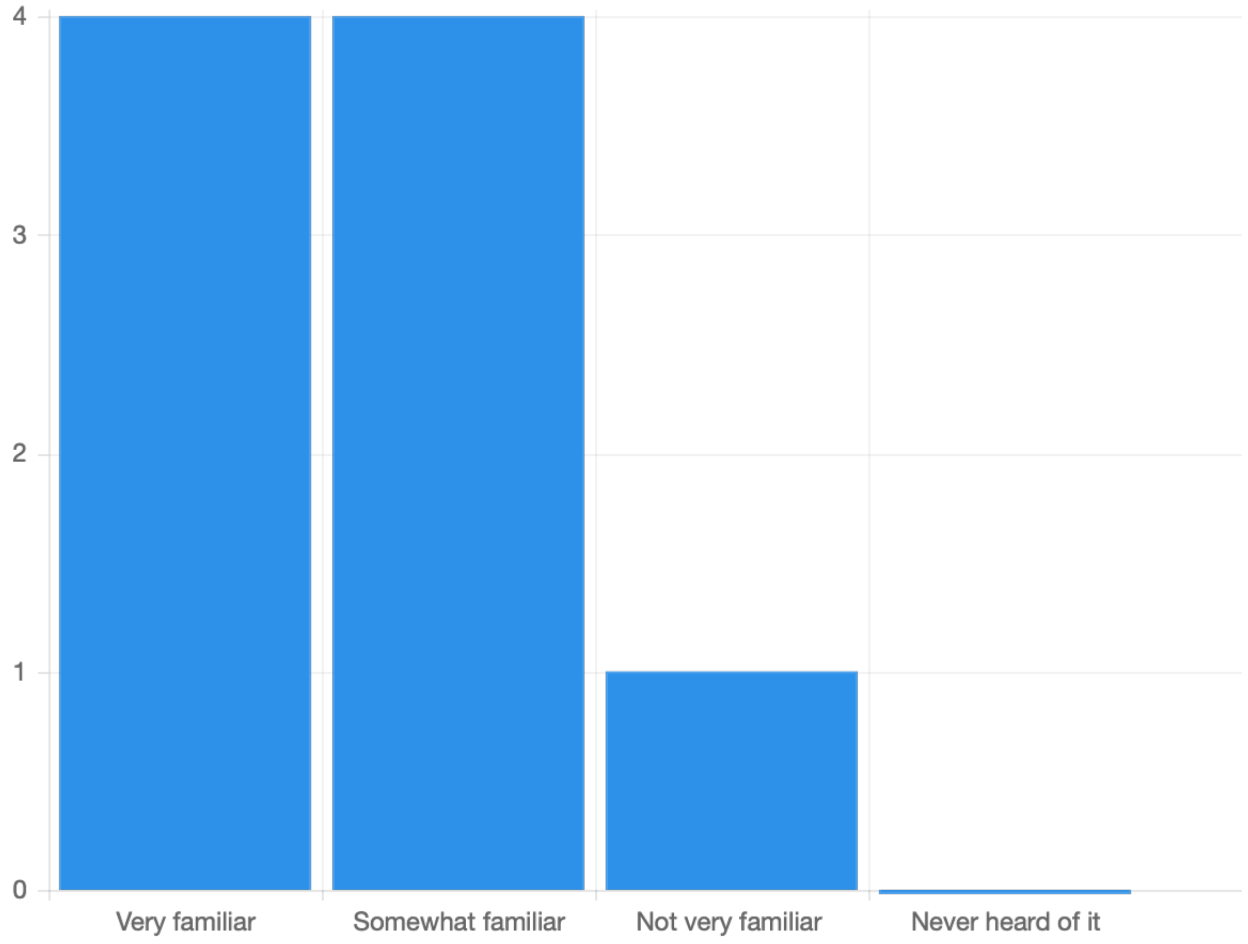

Legend

answers: 9
skips: 0

Answer Explanations

Expert 9 Explanation
Selected Answer(s): Not very familiar

Although it sounds like a very useful concept. I am more familiar with the broader concept of between- and within-person variance.

Expert 3 Explanation
Selected Answer(s): Somewhat familiar

I regularly use the ICC in my exposure assessments, but I am often constrained by the limited amount of data I receive from colleagues, as they often do not repeat measurements on the same person.

Expert 4 Explanation
Selected Answer(s): Very familiar

Similar to the response above.

Expert 2 Explanation
Selected Answer(s): Very familiar

As an experienced biostatistician, I am very familiar with the intraclass correlation coefficient (ICC) within the context of exposure biomarkers due to its crucial role in quantifying the proportion of total variability in biomarker levels that can be attributed to between-person differences, as opposed to within-person variability. The ICC is a key metric in exposure assessment studies, particularly those involving repeated measures or clustered data, as it helps in understanding the reliability of biomarkers over time and the consistency of exposure levels within individuals.

In my work, I have frequently utilized the ICC to evaluate the degree of variability and to guide the design and analysis of studies, ensuring that both within- and between-person variability are appropriately accounted for in statistical models. For example, in longitudinal studies where repeated measurements are taken from the same individuals, and in studies aimed at distinguishing between persistent and transient exposure levels, I have applied and interpreted the ICC to accurately assess exposure, reduce measurement error, and improve the precision of epidemiological findings.

Expert 6 Explanation
Selected Answer(s): Very familiar

See my response to 1.3 above. I think this paper would benefit from a discussion (even if brief) of the need for repeat measurements of study outcome, especially for those health outcomes known to vary within individuals, such a blood pressure, pulse, and hormone levels.

Expert 7 Explanation
Selected Answer(s): Somewhat familiar

A higher ICC indicates that a larger proportion of the total variability is due to between-person differences, meaning the biomarker measurements are more consistent within the same individual across different time points. a desirable biomarker will have high ICC>

Expert 1 Explanation
Selected Answer(s): Somewhat familiar

The use of the ICC to assess the variability is an important tool in biomonitoring. However, new methods are being developed to assess longitudinal studies with repeated measurements. Obviously, the inclusion of other individual factors such as age, gender, ethnicity, body mass index and any pathologies that may affect the biomarker of interest are important.

Expert 8 Explanation
Selected Answer(s): Somewhat familiar

Used this in my studies + in my courses on exposure assessment

---

Expert 5 Explanation
Selected Answer(s): Very familiar

Very familiar - I've conducted field research, taught and published in this area.

Comments (1)

SCORE **3**

Expert 3 *09/08/2024 15:37*

Expert 6 raises a very good point about the importance of discussing the need for repeat measurements. I believe it would be beneficial to expand the discussion further and incorporate the aspects mentioned by Expert 2. Additionally, I wonder if including a visual could be particularly useful to show various ICCs.

Result 2.1 *(ID: 6541)*
Question 2.1 *(ID: 5893)*

Are the assumptions and statistical models detailed in the white paper appropriate for the intended purpose of informing the challenges of exposure biomonitoring variability in epidemiological studies?

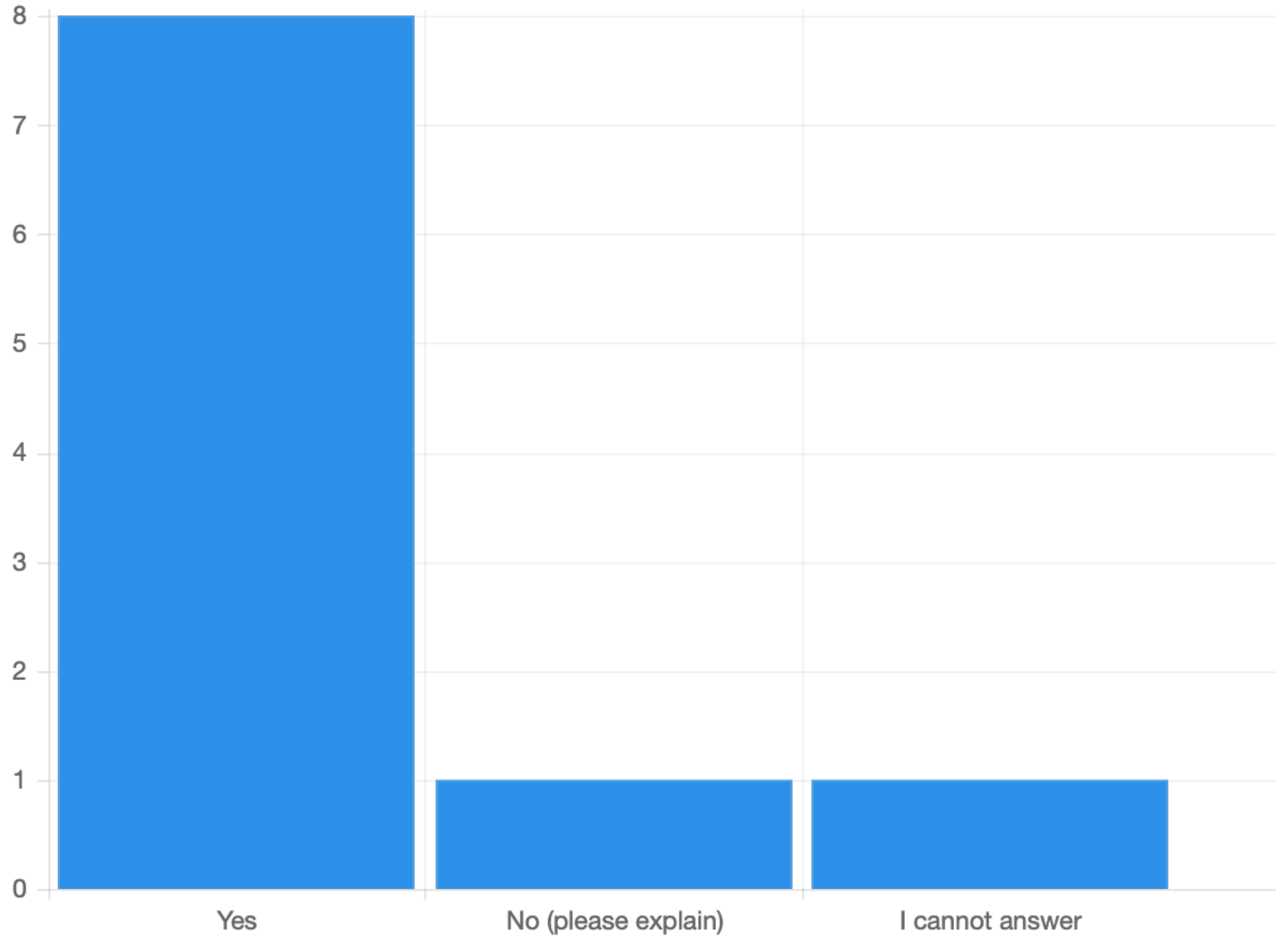

Legend

answers: 9
skips: 0

Answer Explanations

Expert 9 Explanation
Selected Answer(s): Yes

The references and source papers help in this regard - thanks.

Expert 3 Explanation
Selected Answer(s): No (please explain) I cannot answer

The presented calculators are good extensions of other power calculators, but they do not go far enough. This is a question best addressed by an occupational biostatistician. I am responding as an advanced user of statistical

tools, but I cannot address all the statistical details comprehensively. In a recent research project, I attempted to estimate the same quantities as those solved by the presented calculators, and we needed to use simulations. I have several concerns with the proposed methodologies. The points below may address issues that are only tangentially related to the assumptions.

1. **Distribution Assumption**: Exposures are typically log-normally distributed, but the white paper assumes a normal distribution.

2. **Variability Consideration**: Both *exposures* AND *biomarkers* vary between and within subjects. The calculators need to address both types of variability.

3. **Methodological Concerns**: The proposed calculators present closed-form solutions. However, I understand that simulations are often necessary to estimate sample size accurately, especially since random mixed-effects models best describe exposures. The presented linear and logistic regressions will likely overestimate sample sizes and the number of repeats needed. The key issues include whether variances are homogeneous and simple, whether the design is balanced, the consideration of only random intercepts, the hypothesis test, and the within-subject correlation structure. [Confirmation with an occupational biostatistician required.]

4. **Missing Calculations**: The calculations for contrast, attenuation, and bias are missing. These parameters are required in exposure assessment publications and can be estimated from linear mixed-effects models, which are used to model exposures.

5. **Literature and Justification**: The justification for the calculators relies on traditional approaches. However, between- and within-subject variance issues were addressed in exposure assessment as early as 1993 by Kromhout et al. in "A comprehensive evaluation of within- and between-worker components of occupational exposure to chemical agents." A rich body of literature, including several textbooks on exposure assessment, addresses these issues. For example, Rappaport and Kupper's textbook "Quantitative Exposure Assessment" discusses sample size calculations, incorporating observed variance data from studies and sampling strategies.

6. **Variance Range**: The Lin et al. (2005) paper on observed exposure variance suggests different ranges for variances and ICC than those accounted for by the calculators.

7. **Sampling Strategies**: Group strategies can optimize exposure sampling, but the calculators do not address these issues.

8. **Default Values**: The default values do not cover all necessary scenarios. For example, power values of 0.7 or even 0.6 may need to be included in the calculators. ICCs are often much smaller.

9. **Biomarker vs. Exposure Samples**: Whether biomarker samples or exposure samples (e.g., air samples) are better suited for an epidemiological study has not been addressed. Again, see Lin et al.'s paper.

10. **Focus on Significance**: The emphasis on significance is concerning because significance is a function of sample size. The focus should be on effect size instead.

11. **Time Assumptions**: The half-life of biomarkers is not included in the calculators. For example, Preau et al. (2010) in "Variability over 1 Week in the Urinary Concentrations of Metabolites of Diethyl Phthalate and Di(2-Ethylhexyl) Phthalate among Eight Adults: An Observational Study" shows that half-life is crucial for calculating the number of repeats and samples needed as well as when to take the samples.
12. **Need for Pilot Studies**: Brunekreef et al. (1987) suggested that variance components should first be estimated in a pilot study. Epidemiological studies cannot simply start without these estimates. The calculators require point estimates, such as significance and power, among others. It would be better to emphasize that these values need to be observed in a pilot study or obtained through simulations, such as Monte Carlo methods, to estimate an optimal sample size and the number of repeats needed.

Expert 4 Explanation

Selected Answer(s): Yes

Classical measurement error model is assumed and this should be appropriate.

Expert 2 Explanation

Selected Answer(s): Yes

The assumptions and statistical models detailed in the white paper are generally well-suited for addressing challenges related to exposure biomonitoring variability in epidemiological studies. The emphasis on classical measurement error and the use of mixed-effects models aligns well with many common scenarios in biomonitoring research. However, these assumptions may not fully account for all potential complexities, such as non-classical errors or non-normally distributed data. Therefore, caution should be exercised when applying these models and calculators to ensure they are appropriate for the specific study context.

Expert 7 Explanation

Selected Answer(s): Yes

However, Berkson error is not included. It can be relevant in biomarker studies, especially when biomarkers are used to assign exposure levels or when group-level or averaged data are used.

Expert 1 Explanation

Selected Answer(s): Yes

I think the statistical assumptions on which the online calculators are based are sound and come from well cited publications and literature.

Expert 8 Explanation

Selected Answer(s): Yes

I have some general feedback for the white paper, not sure if this is the right place to report them, but will add some here in the comments + will keep more extensive notes for the subsequent discussions and meetings.

General, related to the questions below: not sure if I would make such a clear distinction in logistic versus linear regression models. There are many more models, many assuming linearity, so just using 'linear' may be confusing. Maybe clarify the distinction as 'dichotomous' or categorical outcomes versus continuous outcomes, as measurement error in the exposure may indeed impact estimated associations these types of outcomes differently. For random error in exposure assessment, associations with continuous outcomes are generally

attenuated (biased towards null), while for associations with dichotomous outcomes this may go either way. Sorry for the lengthy comment, but to summarize, there are indeed crucial differences, just not sure if I would refer to them as 'linear' versus 'logistic'.

Also: would not use 'bias analysis' as this seems too simplistic. There are many types of biases in epi study designs and bias in exposure assessment and subsequent consequence for estimating associations with outcomes is just one of them.

Expert 5 Explanation

Selected Answer(s): Yes

I have some edits/suggestions for the manuscript that I can share at some point, but I did not find any issues with the assumptions and statistical models.

Comments (2)

SCORE 1 **Expert 4** *09/01/2024 02:31*

Agree to Expert 3's comments about the issues of the calculator. The authors could consider adopting these assumptions / parameter settings to their calculator.

SCORE 4 **Expert 2** *09/03/2024 07:38*

I agree that Expert 3 provides valuable insights on improving these statistical tools. However, we must also consider the balance between realism and idealism. Highly complicated tools are not easily adopted by a broader audience, especially those with limited statistical and epidemiological knowledge. In practical situations, for example in sample size or power calculations, we do not expect to give an exact value. Instead, providing an approximate range that informs users of potential uncertainties in their estimations is more helpful. As an experienced statistician, I believe that a simplified, suboptimal, but more user-friendly solution would be more popular, without significantly compromising scientific integrity.

Result 2.2 *(ID: 6542)*
Question 2.2 *(ID: 5894)*

How useful are the calculators for estimating the power of epidemiological studies under varying levels of measurement error and sample sizes in the presence of classical measurement error in exposure?

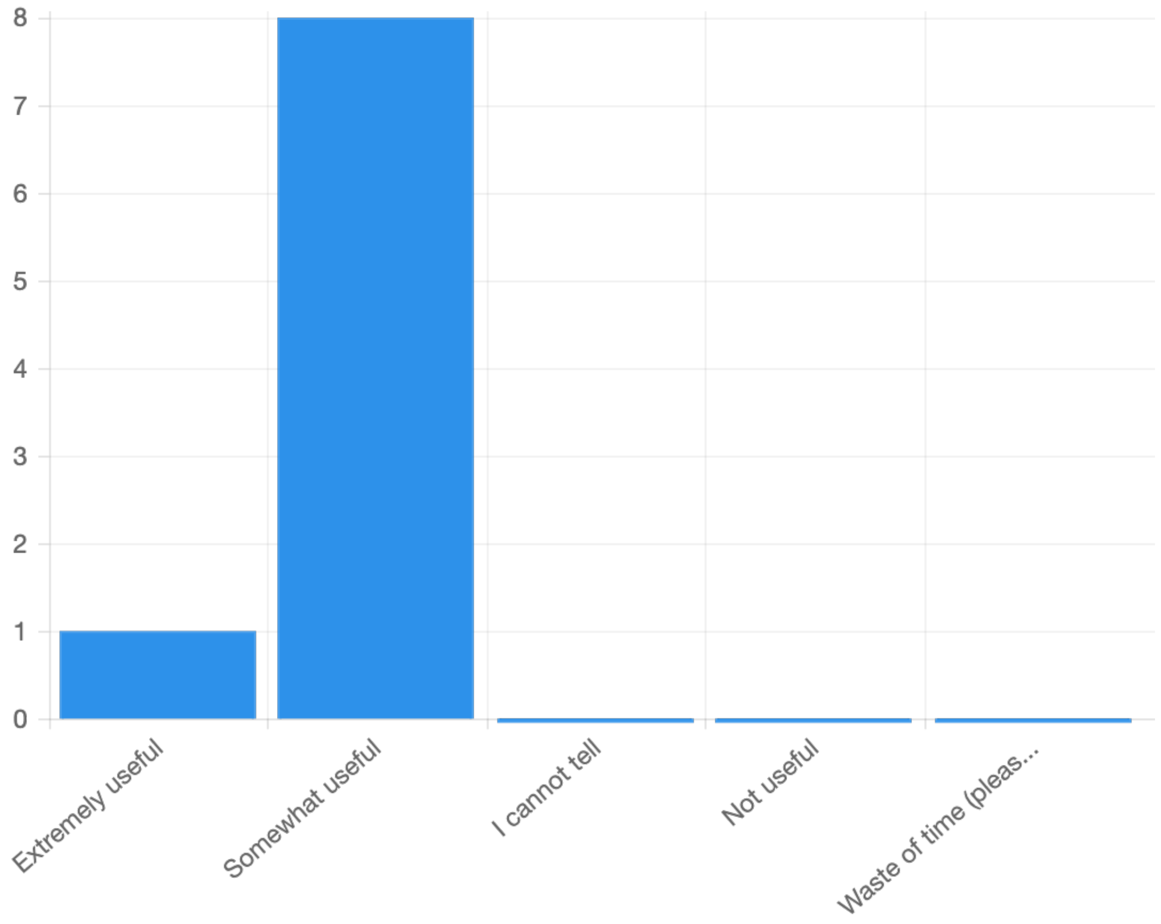

Legend

Extremely useful: 1
Somewhat useful: 8
I cannot tell: 0
Not useful: 0
Waste of time (please explain): 0

answers: 9
skips: 0

Answer Explanations

Expert 9 Explanation
Selected Answer(s): Somewhat useful

Usefulness likely varies with the exposure(s) and the available data on variability associated with them.

Expert 3 Explanation
Selected Answer(s): Somewhat useful

The calculators seem more like a textbook, where individual aspects are presented separately for understanding. The properties of the calculators appear to be interrelated, and I would expect a single calculator that combines all these properties for a more comprehensive analysis.

Expert 4 Explanation
Selected Answer(s): Somewhat useful

It is useful at study design stage to maximise the statistical power by different combinations of repeated measurements and sample size.

Expert 2 Explanation
Selected Answer(s): Somewhat useful

While the calculators presented in the white paper offer valuable insights and tools for optimizing study design in the presence of classical measurement error, I would characterize them as "somewhat useful." This is because the calculators focus on basic and classic scenarios, such as additive, normally distributed measurement errors, which may not fully capture the complexities of many real-world epidemiological studies. For instance, they do not account for the presence of confounders, which are critical in most epidemiological analyses. Moreover, the calculators do not consider different study designs, such as cohort studies, case-control studies, or time-to-event analyses, which have unique statistical requirements and challenges. Without these considerations, the applicability of the calculators is limited to a narrower set of circumstances, making them somewhat useful but not universally applicable for all epidemiological research contexts.
Nevertheless, the calculators offer a convenient and free alternative to more expensive commercial software for sample size and power determination, such as PASS, and they help avoid the need for advanced programming in statistical software like SAS or Stata.

Expert 6 Explanation
Selected Answer(s): Somewhat useful

They would be more useful if they did a better job of explaining the units of the component parameters, such as the mean, standard deviation, variance, desired margin of error, and minimal detectable effect. Most of these parameters are expressed as standardized (SD) units, which may not be easily interpretable by readers.

Expert 7 Explanation
Selected Answer(s): Somewhat useful

Calculators are useful, however their use
requires lots of info not usually available.
What if ICC is unknown?

Expert 1 Explanation
Selected Answer(s): Extremely useful

Being able to see the differences in Power, Sample Size and Measurement error allow the scientist great flexibility to see given the limitations of funding, sample size, the exact values for Power that can be reached.

Expert 8 Explanation
Selected Answer(s): Somewhat useful

Definitely useful, as always, simple easy to use calculators come with a trade-off of flexibility in making changes by the researcher. But those that have ample experience will likely do them themselves or know how to handle their specific data and assumptions. For those that are less familiar and would otherwise maybe not calculate power taking into account measurement errors this will be extremely helpful.

Expert 5 Explanation
Selected Answer(s): Somewhat useful

I think these are most useful for individuals who typically don't do sample size or power calculations incorporating measures of within-individual variation or exposure measurement error.
In my area of research (occupational/environmental epi), Epidemiologists/Statisticians would typically write their own programs (in SAS or R) to estimate sample sizes and tailor them to their specific study.

Comments (5)

SCORE 3 **Expert 4** 09/01/2024 12:33

Expert 5's comment about writing own programs for sample size estimation is a reality.
If the authors' targets are epidemiologists / statisticians, they need to improve the flexibility of the calculator.

SCORE 4 **Expert 2** 09/03/2024 07:47

I believe the target audience for these tools is not statisticians or epidemiologists, as there are already many complex and specialized packages available to address these issues. Instead, these tools are designed for those with less expertise in power or sample size calculation, but who are still aware of the variability within and between individuals. As I previously mentioned, simplified and user-friendly tools are needed to reach a broader audience.

SCORE 2 **Expert 5** 09/11/2024 11:39

These responses indicate to me that the calculators may be most useful for researchers not well trained in these concepts, as expert 2 says - to reach a broader audience.

I would add that we always encourage our students/colleagues to consult with a statistician/epidemiologist (especially if they are clinical/MDs) prior to submitting any grant application/initiating research/and ongoing. Calculators may be handy for sample size and power calculations, but more advanced expertise is usually needed.

SCORE 1 **Expert 8** 09/12/2024 14:47

There seems to be a consensus that these calculators are mostly useful for researchers less experienced in power calculations and that those with more expertise will likely require/prefer more flexibility for more complex data.

SCORE
0

Expert 7

09/13/2024 01:36

As we judge that these calculators and sample size calculations in general mostly useful, it might be helpful to discuss limitations of such calculations. Is anyone aware of a comparison between sample size calculations and the resulting sample size of studies (due to missing data, resource limitations etc.)

Result 2.3 *(ID: 6543)*
Question 2.3 *(ID: 5895)*

How useful are the calculators for estimating the bias in odds ratio of epidemiological studies under varying levels of measurement error and sample sizes in the presence of classical measurement error in exposure?

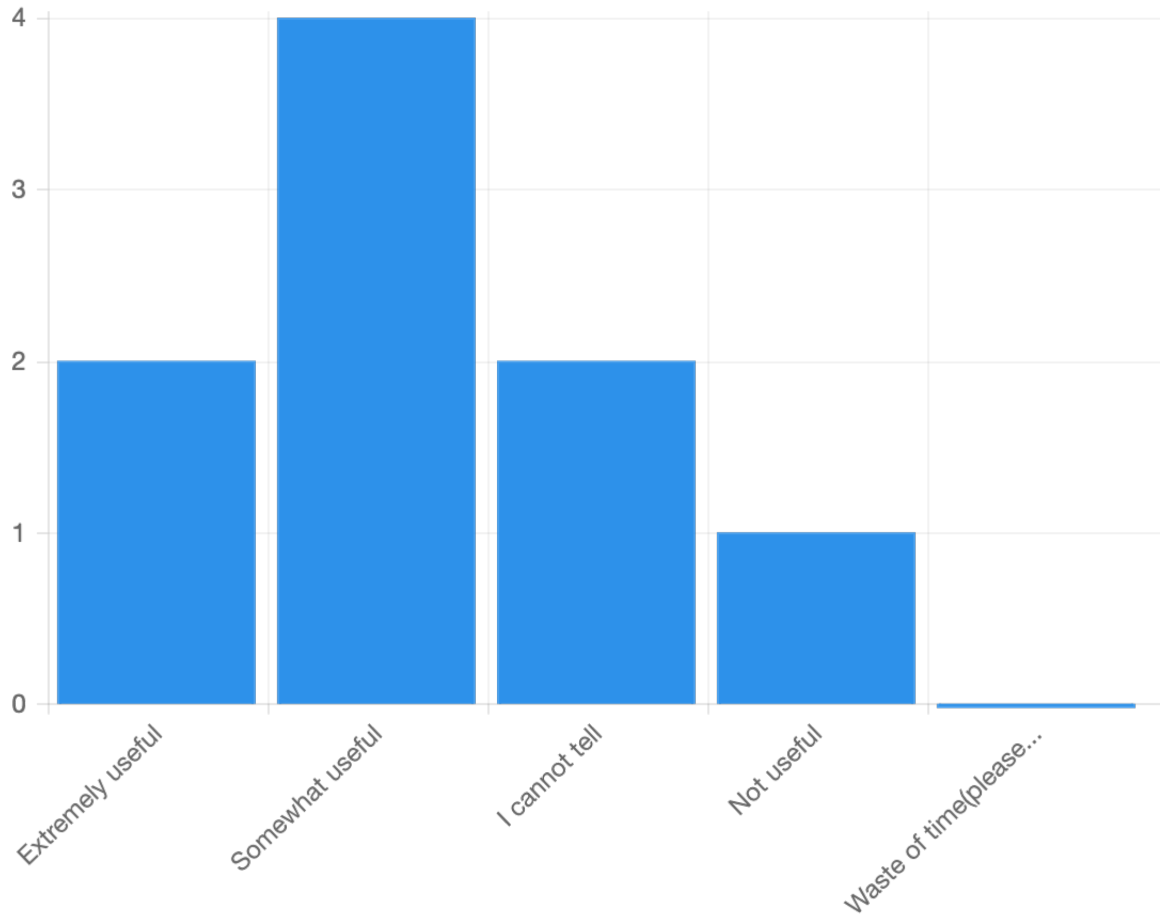

Legend

Extremely useful: 2
Somewhat useful: 4
I cannot tell: 2
Not useful: 1
Waste of time(please explain): 0

answers: 9
skips: 0

Answer Explanations

Expert 3 Explanation
Selected Answer(s): Not useful

In my field, odds ratios are not recommended because they are non-directional and tend to inflate the effect. The relative risk would be a more appropriate and reliable measure.

Expert 4 Explanation
Selected Answer(s): Somewhat useful

Somewhat useful because epidemiological studies usually have broad research questions and we would not estimate the sample size base on one single outcome. However it will still be useful to specify the minimal detectable effect under the planned sample size.

Expert 2 Explanation
Selected Answer(s): Somewhat useful

The calculators specifically address the challenge of measurement error, which is known to bias estimates like odds ratios in epidemiological studies. By simulating the effects of varying levels of measurement error, the tools help researchers understand how such errors might distort their results. While the calculators are useful, as I mentioned above, they do have limitations, such as their focus on classical measurement error models and the exclusion of confounders or complex study designs. Despite these limitations, the calculators offer valuable insights into how measurement error can impact odds ratios, helping researchers design studies that mitigate bias.

Expert 6 Explanation
Selected Answer(s): Somewhat useful

They would be far more useful if the OR varied more widely than 1.1 or 1.2. Observational studies, no matter how well designed, cannot reliably identify causal effects of environmental exposures with such small increases in risk (odds). Such studies usually aim to detect larger increases in risk, with OR in the 1.5-2.0 range.

Expert 7 Explanation
Selected Answer(s): Extremely useful

Bias is often discussed but not estimated. Ability to postulate its potential magnitude is very useful.

Expert 8 Explanation
Selected Answer(s): Somewhat useful

More tricky, as associations with dichotomous outcomes can be either over- or underestimated with random error in exposure assessment, but definitely helpful!

Note here: odds ratios from logistic regression models are often analyzed and presented, but please consider other types of common outcomes and models, especially models taking into account repeated/longitudinal data or other aspects of time. E.g. survival models (cox models); for which effects of measurement errors in the exposure are somewhat similar to logistic models but a bit more complex with a time component.

Expert 5 Explanation
Selected Answer(s): I cannot tell

I'm not sure how useful the program is - I note that the highest OR in the calculator is 1.2. This is a very low OR that one would typically see in an environmental epidemiology or nutrition study. It would be helpful if the calculator allowed for a higher OR to be set.

Also, it would be a good idea to have someone run additional simulations for the logistic regression analysis to confirm the unexpected finding of increased bias. Perhaps expand the discussion in this section with some referenced papers so we can more effectively evaluate this unexpected result.

Comments (2)

SCORE **5** **Expert 4** *09/02/2024 05:32*

Some experts raised the importance of expanding the possible range of OR beyond 1.2 which make sense to me. I am also looking forward to extending the calculator for other effect size measures, e.g., relative risk (expert 3) and hazard ratio (expert 8)

SCORE **5** **Expert 2** *09/05/2024 06:10*

I agree. The settings for the parameters in the tools could be more flexible. For example, allowing adjustable effect sizes and offering optional relative risk measures such as hazard ratios, risk ratios, or incidence rate ratios would enhance their utility.

Result 2.4 *(ID: 6544)*
Question 2.4 *(ID: 5896)*

How likely are the calculators to improve the design of epidemiological studies involving biomonitoring of exposure?

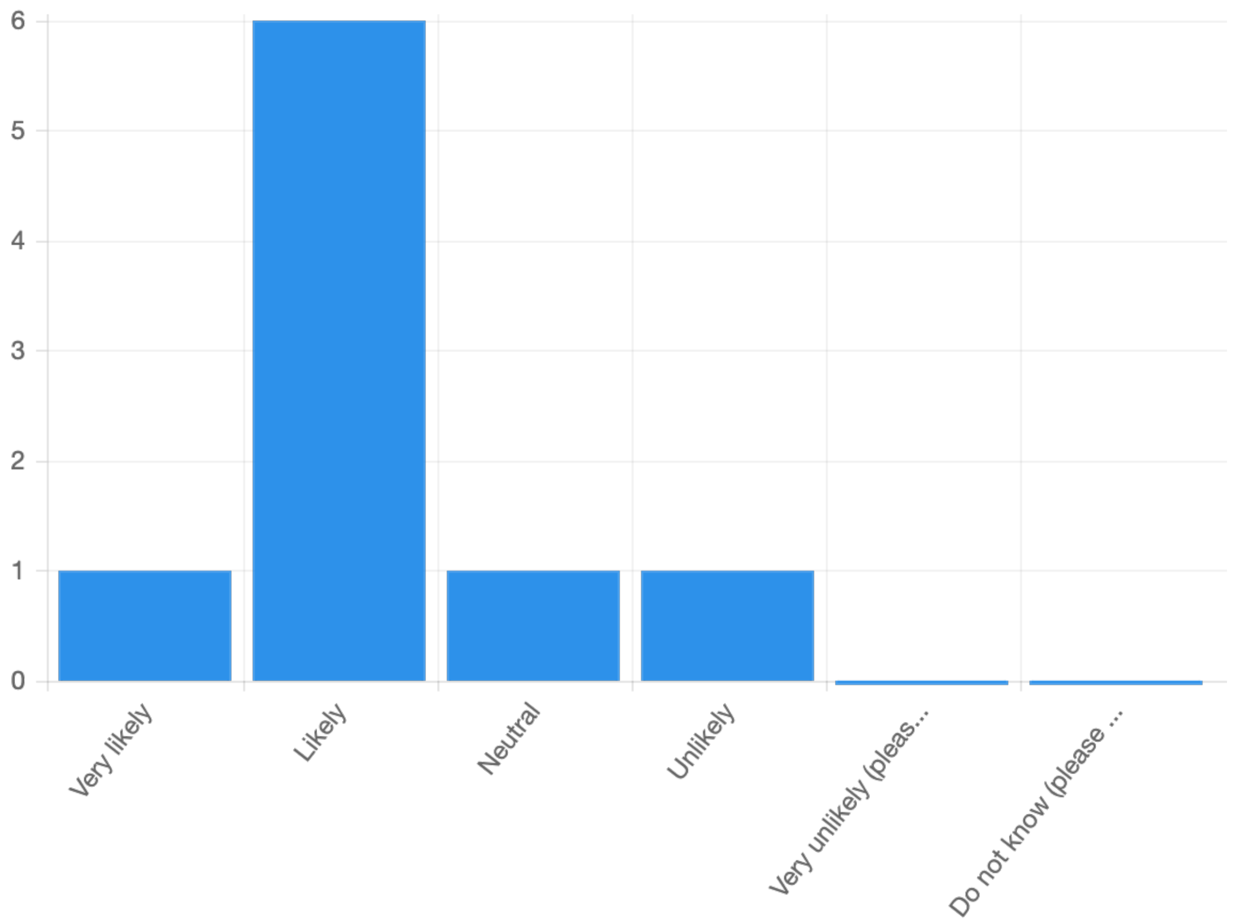

Legend

Very likely: 1
Likely: 6
Neutral: 1
Unlikely: 1
Very unlikely (please explain): 0
Do not know (please explain): 0

answers: 9
skips: 0

Answer Explanations

Expert 9 Explanation
Selected Answer(s): Likely

As presented, these calculators seem likely to be used and if that happens, could improve the design of these studies. However, to answer this question adequately requires a comparison to other calculators and resources already available to researchers, which I recommend in my other comments.

Expert 3 Explanation
Selected Answer(s): Unlikely

I do not see a substantial improvement over existing calculators. The presented calculators do not seem to address current needs effectively. Moreover, I do not expect most epidemiologists to use them, as the information on exposure sample size calculations is several decades old. The issue seems more psychological than technical.

Expert 4 Explanation
Selected Answer(s): Likely

The classical measurement error framework should be used at design stage, but unfortunately it was seldom used in the literature. Hopefully the current calculator can promote this framework to non-statisticians.

Expert 2 Explanation
Selected Answer(s): Likely

I recognize that the calculators provide a handy toolkit for improving epidemiology study designs by addressing key aspects that are often overlooked, such as the need for repeated measurements and the trade-offs between sample size and measurement error. However, these calculators are focused on classical measurement error and do not account for all the complexities typically encountered in real-world studies.
Overall, while these calculators are likely to improve the design of epidemiological studies by helping researchers better account for measurement error in biomonitoring data, leading to more robust and valid findings, they are limited in scope. In practice, the design of epidemiological studies involving biomonitoring for exposure assessment is far more complex than the scenarios considered by the calculators. Factors such as the number of covariates/confounders, skewed error distributions, uncertainty in the relationships between covariates (covariance issues), and non-linear exposure-response relationships (which have become more evident in recent years) are critical elements that these tools do not fully address. Therefore, while useful, the calculators can only partly assist in considering the full complexity of epidemiology study designs.

.

Expert 6 Explanation
Selected Answer(s): Likely

They would be even more useful with the changes I recommended above. Inclusion of within-subject variability in outcome would also be helpful.

Expert 7 Explanation
Selected Answer(s): Likely

I think sample size calculations are rarely useful, because sample sizes are often driven by the available data or resources. I think these calculators are more useful to understand the trade-offs between sample sizes (number of individuals sampled, n), number of repeats (samples per individual, m). As well as evaluations of biases in estimated regression coefficients.

Expert 1 Explanation
Selected Answer(s): Very likely

I think researchers, clinicians, and students would be very likely to use these calculators. Not only do they provide the individual with the calculation process and formulas, but they importantly provide graphical representation of the calculation itself. This will be very useful when trying to work out a sample size, Power, Bias, etc... for a given budget and what the optimal data output will be for a given exposure plan. Based on my own experience with online epidemiological calculators, when I find one that provides all the information I am looking for, is easy to use, and is updated regularly with references and bug fixers I use them all the time. However, it can be very frustrating when these calculators for no reason disappear only to leave me with no other good option! I think an online site with these calculators available for biomonitoring studies would be an excellent resource that many would use daily!

Expert 8 Explanation

Selected Answer(s): Likely

In line with my previous answer: particularly useful for those who would otherwise not incorporate it. But I would also use them, or share them with my students and postdocs.

Important in study proposals; in making sure that the measurement methods, frequency of measurements, and sample sizes are adequate to answer the research question. Very important in avoiding more studies with a conclusion 'we did not find any associations, but that may be because of measurement error / being underpowered'. The latter is unfortunately very common and a waste of research time/energy/materials, it's crucial to think of this before starting any studies and improving methods or sample size to make sure methods and sample sizes are adequate.

Expert 5 Explanation

Selected Answer(s): Neutral

They could possibly as they are very user friendly and accessible. However, there are a number of other calculators and programs published in the literature, various websites, SAS or R programming guides, that may do the same type of power and sample size calculations.

Comments (2)

SCORE 3 **Expert 2** 09/05/2024 06:15

I'm pleased to observe that the experts' opinions are largely consistent.

SCORE 0 **Expert 9** 09/12/2024 15:30

While I think these calculators are likely to improve the design of studies, they may also be useful in other areas, such as teaching students about different sources of variability in a study and their impact on study design.

Result 3.1 *(ID: 6545)*
Question 3.1 *(ID: 5897)*

How user-friendly are the calculators for researchers designing studies?

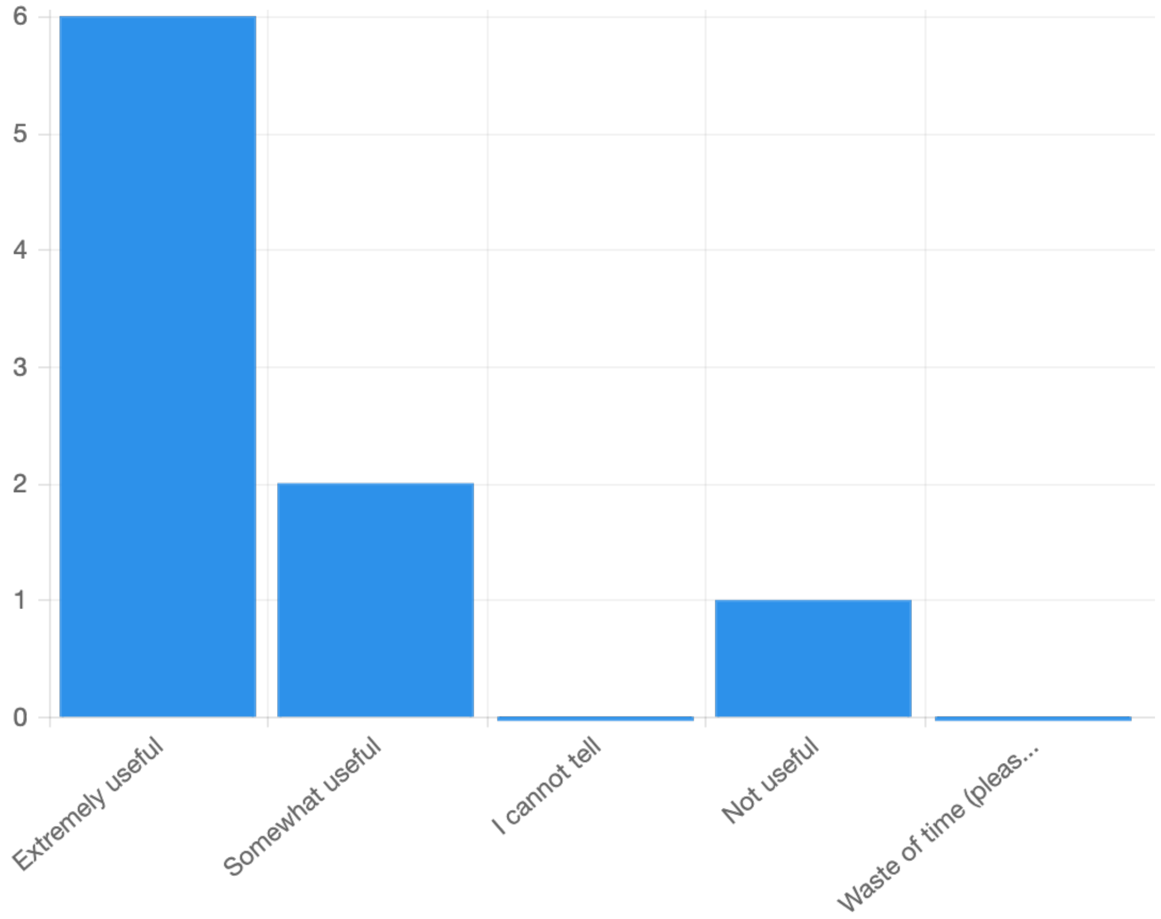

Legend

Extremely useful: 6
Somewhat useful: 2
I cannot tell: 0
Not useful: 1
Waste of time (please explain): 0

answers: 9
skips: 0

Answer Explanations

Expert 3 Explanation
Selected Answer(s): Not useful

The range of parameters is too limited e.g. power, ICC. The calculators only work with a single value at a time. It would be much more user-friendly if they allowed for a range of inputs and provided a range of acceptable outputs. I also find the lack of options to select different outputs, such as Relative Risk versus Odds Ratio, to be a

significant limitation.

Expert 4 Explanation
Selected Answer(s): Extremely useful

Very user friendly, the output is clear.

Expert 2 Explanation
Selected Answer(s): Extremely useful

The calculators are designed with a user-friendly interface, making them accessible even to those without advanced statistical training. The use of sliders, input fields, both tabulated and visualized outputs, along with clear instructions and examples, simplifies the process of inputting data and interpreting results. This makes them a valuable resource for researchers who need to design epidemiological studies but may not have extensive statistical expertise. Their ease of use and accessibility make them a practical tool for improving study design.

Expert 6 Explanation
Selected Answer(s): Somewhat useful

They would be more user-friendly with the changes I recommended above, especially for non-statisticians. They would also benefit from inclusion (or at least discussion) of within-person variability in outcome measurement.

Expert 7 Explanation
Selected Answer(s): Extremely useful

I am answering a question about how how user-friendly are the calculators. For how useful they are see answer to 2.4

Expert 1 Explanation
Selected Answer(s): Extremely useful

I like these calculators, they were fun and easy to use. I really like that they provide graphs showing the comparisons, which also provides the user with ideas about how output will change if a single variable is either increased or decreased.

Expert 9 Explanation
Selected Answer(s): Extremely useful

The instructions are clear and relatively easy to understand. The major impediment is that researchers may not have values of all the input parameters available to them. I have some suggestions for that in my general comments below.

Expert 8 Explanation
Selected Answer(s): Somewhat useful

Seem to be userfriendly to me. I would have to test them for various datasets/studies to check if anything is missing/confusing, but based on my review so far they seem easy to use.

Expert 5 Explanation
Selected Answer(s): Extremely useful

I think these calculators are very user friendly

Comments (2)

SCORE
1

Expert 3

09/08/2024 15:49

I agree with Expert 6.

SCORE
1

Expert 5

09/11/2024 11:45

Please note that I answered the question whether the calculators were "user-friendly" not if they were extremely useful. I think there was an error in the response categories.

Result 3.2 *(ID: 6546)*
Question 3.2 *(ID: 5898)*

Do the calculators effectively balance complexity with usability?

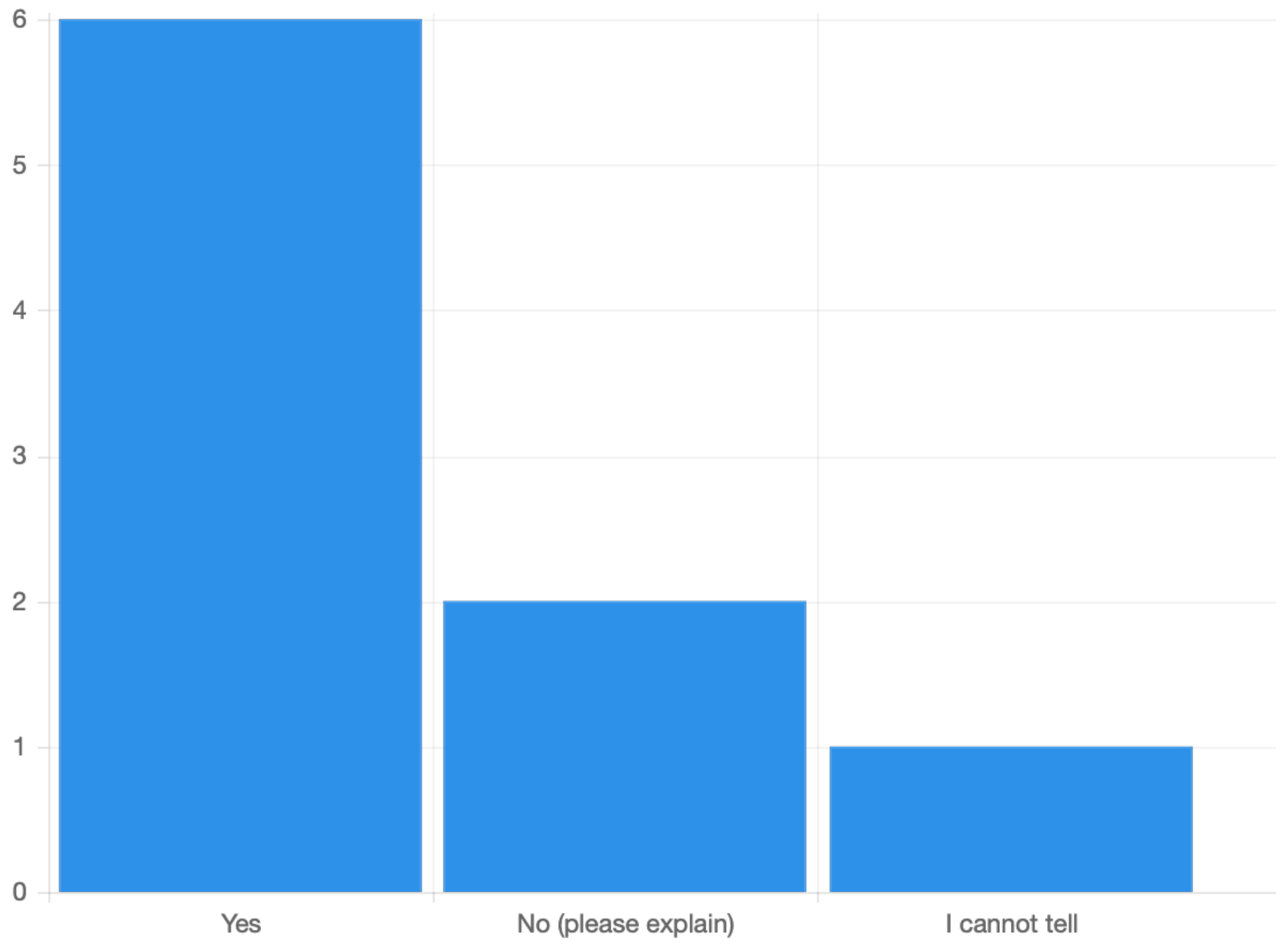

Legend

answers: 9
skips: 0

Answer Explanations

Expert 3 Explanation
Selected Answer(s): No (please explain)

The calculators are indeed user-friendly, particularly since they are made in Shiny, but they lack the necessary complexity. Important options are missing, which limits their usefulness. It would be much more user-friendly if they allowed for a range of inputs and provided a range of acceptable outputs. Additionally, the absence of options to select different outputs, such as Relative Risk versus Odds Ratio, is a significant limitation.

Expert 4 Explanation
Selected Answer(s): No (please explain)

Manual input in parameters should be allowed, for example calculator 4.

Expert 2 Explanation
Selected Answer(s): Yes

The calculators effectively balance complexity with usability by providing a simplified and intuitive interface that allows users to engage with advanced statistical concepts without being overwhelmed. By focusing on key metrics such as the intraclass correlation coefficient (ICC), sample size, and measurement error, the calculators deliver valuable insights without requiring users to manage overly complex parameters. The inclusion of clear instructions and examples enhances usability, guiding users through the process and ensuring they understand the results' implications.

Expert 1 Explanation
Selected Answer(s): Yes

Yes, I believe so. There is a lot of complexity in these calculations, but the layout and structure of the calculators makes them easy to use and understand.

Expert 8 Explanation
Selected Answer(s): I cannot tell

This is always a tricky trade-off for premade calculators/models. It should be simple enough to be used, especially for those without very extensive expertise, but they should be complex/flexible enough to accommodate various study types and datasets.

For this aspect, I think it's crucial to make the code open source, e.g. on github, so that those who need more flexibility/complexity can still use it, modify it for their needs. In my team we previously did that for e.g. spline models to test nonlinearity. One can use a 'basic' easy to use version on a website, but in case of exceptions and complexities in the data, one can access the original R and Python code and modify it for their needs.

Expert 5 Explanation
Selected Answer(s): Yes

I guess so - they are user friendly but limited to the most simple scenario of classical measurement error and only one independent variable. Most often we are dealing with many independent variables, with their own measurement error and correlations with the primary exposure variables of interest. This complicates these type of scenarios, but reflects the real world.

Ideally, these calculators could be expanded to reflect more complicated scenarios!

Comments (2)

SCORE 1 **Expert 3** 09/08/2024 15:54

I agree with Expert 8's suggestion to put the calculators on GitHub. The calculators could be presented at various levels of complexity, with options gradually expanded.

SCORE 1 **Expert 5** 09/11/2024 11:46

Good comments, I have nothing else to add here.

Result 3.3 *(ID: 6547)*
Question 3.3 *(ID: 5899)*

Are the instructions clear and easy to follow?

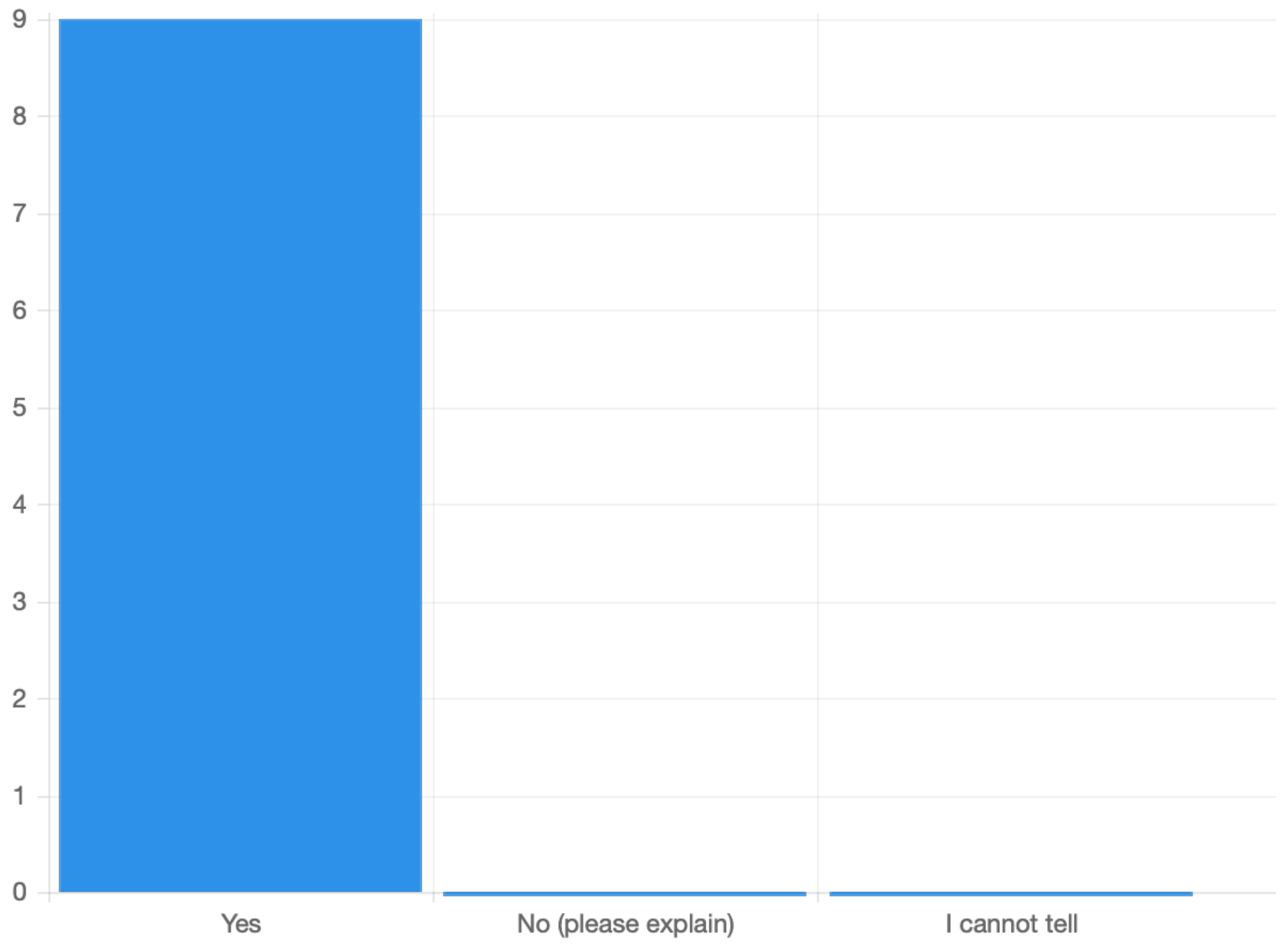

Legend

answers: 9
skips: 0

Answer Explanations

Expert 3 Explanation
Selected Answer(s): Yes

The instructions are clear, but a few edits could improve them further.

Expert 4 Explanation
Selected Answer(s): Yes

Yes after reading the white paper.

Expert 2 Explanation
Selected Answer(s): Yes

The instructions are clear and easy to follow because they provide step-by-step guidance, use simple language, include illustrative examples, and offer visual aids to help users understand and apply the calculators effectively.

However, the instructions could be improved by including relevant formulas and references, as is common in many software packages.

Expert 6 Explanation

Selected Answer(s): Yes

Yes, but clarification of the units (standardization) of the inputs would be helpful.

Expert 1 Explanation

Selected Answer(s): Yes

Yes, I really like the examples provided for each calculator. Perhaps, there could also be a study example referenced for each calculation so the person doing the calculation can see a real world example.

Expert 8 Explanation

Selected Answer(s): Yes

I think the user guide under each of the calculators is clear, while not being too lengthy.

Expert 5 Explanation

Selected Answer(s): Yes

Very clear.

Comments (1)

SCORE 3 | **Expert 5** | *09/11/2024 11:47*

The between variation is zero here. ;-)

Result 3.4 *(ID: 6548)*
Question 3.4 *(ID: 5900)*

Are the assumptions made by the calculators clearly defined in the documentation?

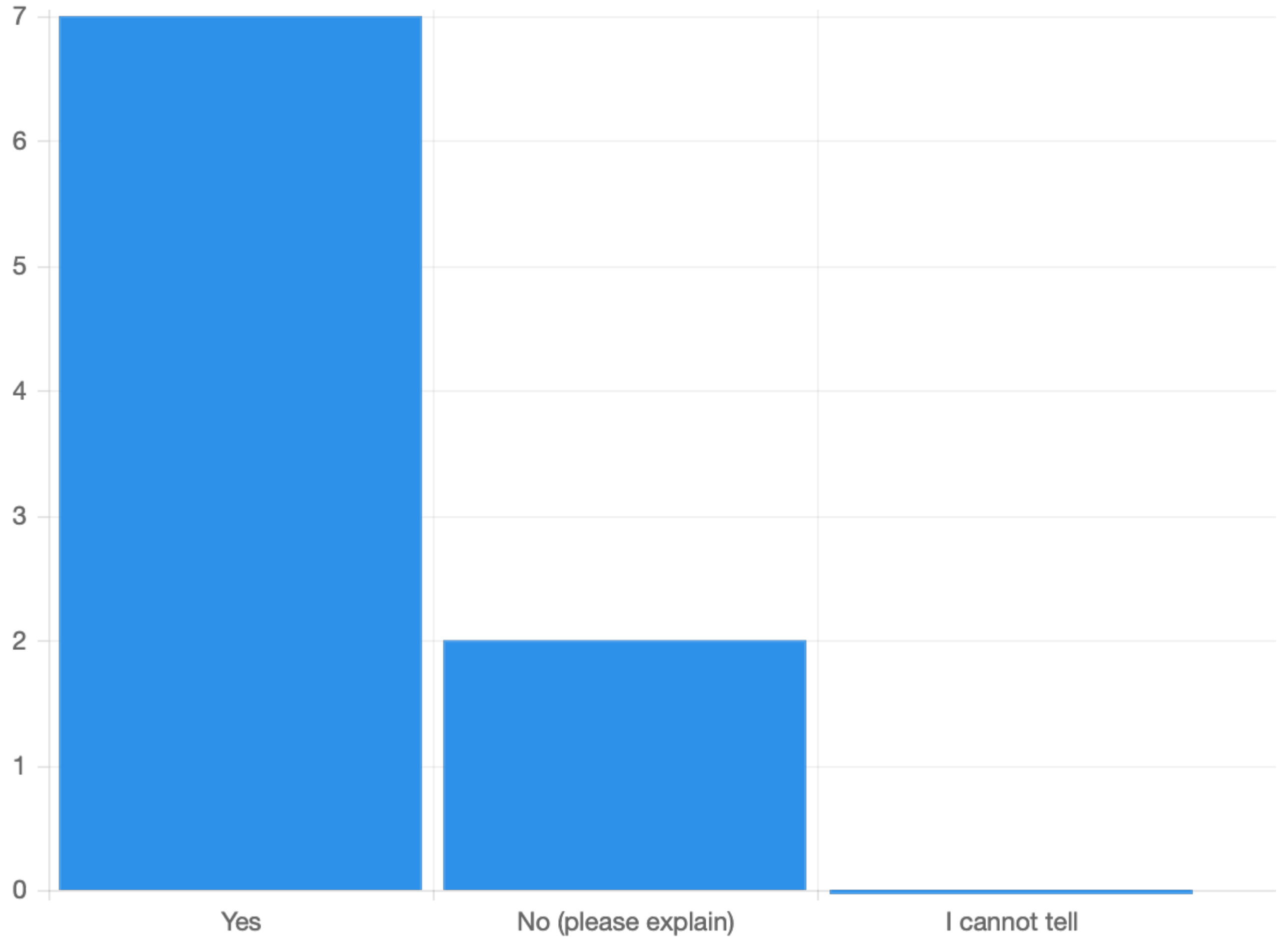

Legend

answers: 9
skips: 0

Answer Explanations

Expert 4 Explanation
Selected Answer(s): Yes

Yes they are all specified.

Expert 2 Explanation
Selected Answer(s): No (please explain)

The documentation clearly states that the calculators assume classical (additive, normally distributed) measurement error. This is a critical assumption that underpins the functionality of the calculators, and it is explicitly mentioned to ensure users understand the basis of the calculations.
However, the documentation did not outline that the calculators are designed for specific, simplified scenarios,

such as focusing on single-exposure variables without considering confounders or complex study designs.

Expert 6 Explanation

Selected Answer(s): No (please explain)

Units of the input parameters need to be clarified. Also, the absence of assumptions about non-variability in outcome measurement should at least be mentioned.

Expert 7 Explanation

Selected Answer(s): Yes

An assumption that sampling and analytical techniques have negligible errors, orders of magnitude smaller than those related to between- and within-person variability in exposure and exposure biomarkers. seems reasonable

Expert 1 Explanation

Selected Answer(s): Yes

Yes, they are clearly laid out in a very readable format with formulas and dot points explaining what each variable represents.

Comments (1)

SCORE 0

Expert 3 09/08/2024 15:59

It seems that the assumptions need further explanation and should be more clearly stated.

Result 3.5 *(ID: 6549)*
Question 3.5 *(ID: 5901)*

Are the input parameters required by the calculators clearly defined in the documentation?

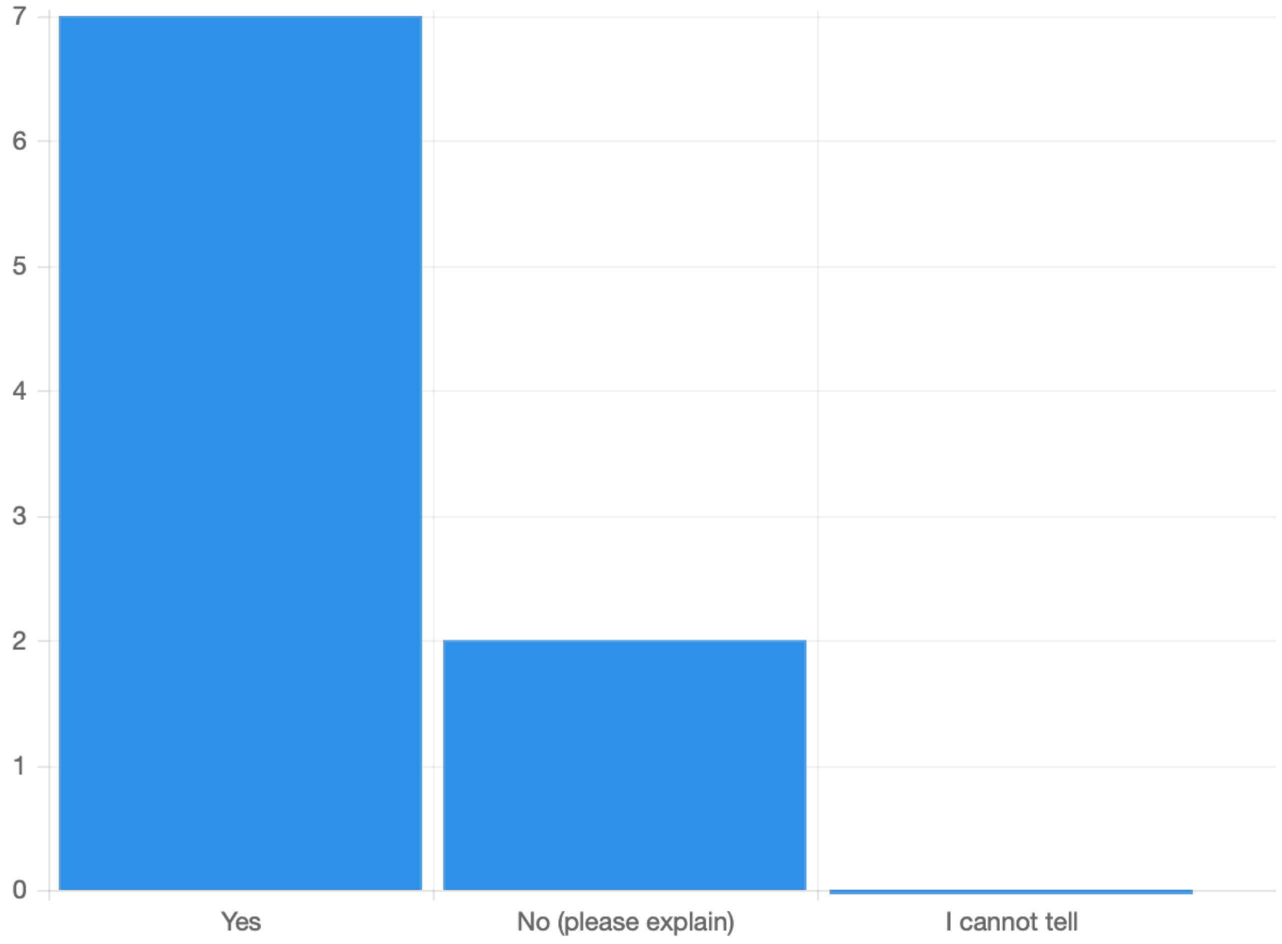

Legend

answers: 9
skips: 0

Answer Explanations

Expert 4 Explanation
Selected Answer(s): Yes

Yes their definitions and the model assumption are defined.

Expert 2 Explanation
Selected Answer(s): No (please explain)

1. I believe that not all users may be familiar with the statistical terms used in the calculators. Therefore, it would be helpful to provide explanations of key concepts like ICC and the validity coefficient within the calculators or user guides. Alternatively, citing relevant references would also be beneficial.
2. For Calculator 2, it was unclear whether the Margin of Error (MOE) is defined as an absolute difference between means or as a percentage. Additionally, in the slider bar for MOE, it is not clear if values like 0.01,

0.21 refer to 0.01%, 0.21%, or 1%, 21%.

3. Similarly, in Calculators 3a and 3b, it should be clarified whether the Minimum Detectable Effect (MDE) is defined as an absolute value or a percentage.
4. In Calculator 4, the term "baseline probability" should be referred to as "background incidence" for greater clarity.

Expert 6 Explanation

Selected Answer(s): No (please explain)

Yes, but clarification of the units (standardization) of the inputs would be helpful.

Expert 1 Explanation

Selected Answer(s): Yes

Yes, I like the simple approach used with the formula and variable explanation for each calculation. This is followed by an example which makes it even easier to understand.

Expert 9 Explanation

Selected Answer(s): Yes

The required input parameters are clearly defined. However, they may not be readily available for every exposure that a researcher is interested in; I have some suggestions regarding that in my general comments below.

Expert 8 Explanation

Selected Answer(s): Yes

Yes. It's great that it's flexible, that one can 'play' with parameters that are not yet known (e.g. in planning a new study) and immediately see the effects on the power and bias analysis. It may be helpful to add examples here, not sure though.

Comments (1)

SCORE 3 **Expert 4** *09/03/2024 11:59*

Agree with Expert 2's comment on the use of statistical terms, especially different fields have their own terms. Suggest to provide some background information about the calculator.

Result 3.6 *(ID: 6550)*
Question 3.6 *(ID: 5902)*

What specific recommendations do you have for improving the calculators?

Expert 3

Consider Distribution Assumptions:

- Adjust the calculators to account for the log-normal distribution of exposures, as the current assumption of a normal distribution may not accurately reflect real-world data.

Address Variability:

- Ensure that the calculators account for variability both between and within subjects, for both exposures and biomarkers.

Incorporate Simulations:

- Include simulations, especially since closed-form solutions may not accurately estimate sample size. Simulations, such as those using random mixed-effects models, are often necessary for precision epidemiological studies with challenging exposure assessments.

Add Calculations for Contrast, Attenuation, and Bias:

- Incorporate the ability to calculate contrast, attenuation, and bias, as these are critical parameters in exposure assessment and can typically be estimated using linear mixed-effects models.

Update Justifications Based on Literature:

- Revise the calculators to incorporate findings from established literature, such as the work by Kromhout et al. (1993) and Rappaport and Kupper's textbook on quantitative exposure assessment. This will ensure the calculators reflect current best practices in the field.

Expand Variance Range Considerations:

- Adjust the calculators to handle the wider range of variances and ICCs observed in studies, as suggested by Lin et al. (2005).

Include Group Sampling Strategies:

- Introduce group sampling strategies into the calculators, as these can optimize exposure sampling and improve study design.

Broaden Default Values:

- Expand the default values to include a broader range of scenarios, such as power values of 0.7 or even 0.6, and consider smaller ICCs.

Compare Biomarker vs. Exposure Samples:

- Provide options to evaluate whether biomarker samples or exposure samples (e.g., air samples)

are more suitable for a given epidemiological study Lin et al. (2005) .

Shift Focus from Significance to Effect Size:

- Emphasize the importance of effect size rather than significance alone, as significance is often a function of sample size and may not fully reflect the practical implications. e.g. having a significance range would be useful.

Consider Time and Half-Life in Calculations:

- Integrate considerations for the half-life of biomarkers into the calculators. This is crucial for determining the number of repeats and appropriate timing for sample collection, as highlighted by Preau et al. (2010).

Emphasize the Importance of Pilot Studies:

- Recommend conducting pilot studies to estimate variance components before starting full epidemiological studies. Pilot studies or simulations, such as Monte Carlo methods, can provide more accurate point estimates for parameters like significance, power, sample size, and the number of repeats needed.

Expert 7

Using multiple measurements of exposure (instead of biomarkers) or at least a combination of exposure measurement when biomarker is not available

Expert 4

Calculator 1:

Sometimes the number of repeats (y-axis) has tick levels at non-integer value (e.g., at intervals of 0.5), need to fix to integer-only.

Consider including the formula and reference in the text.

Calculator 2:

Default value of SD should be 1.

The calculator is for estimating a mean, would you create another calculator for proportion estimation?

Calculator 3a:

I think this calculator is for the user to balance between the sample size (n) and number of measurements per subject (m), so the graph should present them. For the current setting, users need to try different values of m, obtain their corresponding n, and decide which combination to be used. The difference between n_x and n_z is not explained in the white paper.

Calculator 3b:

This calculator is not explained in the white paper. Can the user specify the MDE?

Calculator 4:

There are too few choices to set the parameters. Would be great if the users can enter the values themselves. Would you allow the users to change the number of simulations? It appears that the current simulations are too few that the bias might increase with sample size.

Expert 2

1. Improve the clarity of the terms used in the calculators, particularly those mentioned above.
2. Include brief explanations or tooltips for key statistical terms in the user guides, such as ICC and validity coefficient. Additionally, provide references to relevant literature or resources for users who wish to explore these statistical concepts further. This could be done through a "Learn More" link or a short list of references in the user guide.
3. Enhance the accuracy of labeling in the calculators to clearly indicate the units being used (e.g., “0.01 (1%)” for percentages) to help prevent any misinterpretation of input values.

Expert 6

1. Clarify the units of the inputs.
2. Include more realistic (higher) OR inputs.
3. Incorporate within-person variability in outcome measurements.
4. The x-axis of Figure 2 should be labeled as "sample size," not "number of samples."
5. There seem to be two Figure 4's, without clear reason.
6. Figure 5 should make it clear that it pertains to logistic regression.

Expert 1

For calculator #4 the sensitivity analysis explorer, perhaps the odds ratio could be wider along with the sample size <100 and >800. Some people may have a limited budget which may limit the sample size and others might want to see a reasonable effect size (or: 2.0).

Expert 9

As much as is practical, arrange the information and output similarly across all the calculators.

Calculator #2: Sample Size Calculator for Mean with Desired Margin of Error

1. How does this relate to situations with low ICC? Does this assume that within-individual variance is 0 or negligible?
2. Allow standard deviation to go much higher, for example 20 or 50. For example, the standard deviation for weight among American males is 29 pounds, and for cholesterol is 14.
3. Similarly, allow the desired margin of error to go much higher as well.
4. You might consider offering a desired margin of error to be expressed as a percentage of the mean, rather than as absolute units.

Calculator #3a and b: Sample Size and Minimum Detectable Effect (MDE) Tradeoff Calculator for Linear Regression

1. It seems that sometimes you use the term "within-subject variance" and other times the term "measurement error" to mean the same thing. I suggest either clarifying the relationship between them or using the same term.
2. It could be helpful to readers to explain how to use calculators 3a and 3b together, if that is likely to occur. Could they be combined into a single calculator with the option to set m=1 if within-person variability is not going to be dealt with?

Calculator #4: Sensitivity Analysis Explorer for Logistic Regression

1. It would be helpful to extend the Odds Ratio higher, say to 2.0 or even 3.0 (and lower, to accommodate their inverses, 0.5 and roughly 0.3). Sample sizes needed to identify odds ratios of 1.2 as statistically significant may be so large as to be unrealistic for researchers with limited funding or dealing with exposure measurements with high variability.
2. When I tried using the Shiny App, neither the charts nor the table changed when I changed the Sample Size using the slider.
3. Would it be possible to change this calculator to show the necessary sample size (n X m) or n, m separately? I think that might be more helpful to researchers designing studies.

Expert 8

See previous answers.

Expert 5

Perhaps include options for including additional confounding variables (i.e. X2, X3) in the calculators.

Comments (1)

SCORE 0 **Expert 7** 09/13/2024 00:40

I found suggestions for improvements of Expert 3 particularly important, including

1. adding log-normal distribution of exposures

2. Calculators should account for variability both between and within subjects, for both exposures and biomarkers

3. Ability to evaluate bias

4. Include wider range of variances and ICCs, and power values

5. Ability to evaluate whether biomarker samples or exposure samples (e.g., air samples) or a combination of both are more suitable or available.

6. Integrate considerations for the half-life of biomarkers into the calculators to evaluate the number of repeats and appropriate timing for sample collection.

7. Allow for innovative designs, eg group sampling or replacement of missing biomarkers with exposure

estimates

8. Move from so much focus on statistical significance

9. Importance of pilot studies to estimate parameters needed for the best design of the main study

Result 3.7 *(ID: 6551)*
Question 3.7 *(ID: 5903)*

What are the additional factors or variables, if any, that should be considered to enhance the robustness and generalizability of the calculators?

Expert 3

> From the specific recommendations:
> **Distribution Assumptions**:
>
> - Incorporate options to account for different distribution types, such as log-normal distributions, rather than assuming a normal distribution for all exposures.
>
> **Variability Consideration**:
>
> - Include mechanisms to address both between-subject and within-subject variability for exposures and biomarkers.
>
> **Incorporation of Simulations**:
>
> - Enable the use of simulations, such as random mixed-effects models, to provide more accurate estimates of sample size and account for complex exposure patterns.
>
> **Calculation of Additional Parameters**:
>
> - Add calculations for contrast, attenuation, and bias, which are essential in exposure assessments and can improve the accuracy and relevance of the results.
>
> **Expanded Range of Default Values**:
>
> - Broaden the default values to cover a wider range of scenarios, such as lower power values (e.g., 0.7 or 0.6) and smaller ICCs.
>
> **Flexibility in Output Selection**:
>
> - Provide options to choose between different statistical measures, such as Relative Risk versus Odds Ratio, to better suit the needs of specific epidemiological studies.
>
> **Consideration of Time-Related Factors**:
>
> - Factor in the half-life of biomarkers to determine appropriate sample timing and the number of repeats required for accurate exposure assessment.
>
> **Inclusion of Pilot Studies and Simulations**:
>
> - Encourage the use of pilot studies and simulations (e.g., Monte Carlo methods) to estimate variance components and refine study designs before full-scale implementation.
>
> **Sampling Strategy Optimization**:
>
> - Introduce group sampling strategies that can optimize exposure sampling and enhance the efficiency and accuracy of the study design.

Focus on Effect Size Over Significance:

- Shift the focus from statistical significance, which is dependent on sample size, to effect size, which provides more meaningful insights into a study's findings.

Expert 7

An ability to use a different sample (from a pilot or another study) to estimate exposure or biomarker. Addition of a parameter that indicates how far back the exposure of interest is (number of year) as compared to when biomarker was obtained.

Expert 4

Please see the above response.

Expert 2

1. Consider adding options that allow users to include confounders or covariates. While this may introduce additional complexity, it would enhance the calculators' versatility and applicability to a broader range of study designs.
2. Additionally, explore the possibility of incorporating options for non-linear exposure-response relationships, which are increasingly relevant in epidemiological research.

Expert 6

A brief explanation of how to convert conventional input variables to standardized units.
I don't see the utility of Section 2. The "motivating examples" are separated from the corresponding "data," and no data are shown.

Expert 1

I can't think of any other factors or variables to include.

Expert 9

The comments below actually pertain more to the presentation of this white paper than specific factors or variables.

GENERAL COMMENTS

How will this white paper be released? As a publication in a scientific journal? As a stand-alone tool on the web, together with links to the calculators? This question is relevant because I suspect that the typical structure for scientific papers (Introduction, Methods, Results, Discussion) may not work as well for the purpose of this paper. If the authors have freedom to arrange the paper as they like, I suggest that they consider something more like the following:

- Introduction (background / statement of problem of variability and determining sample size, tools currently existing to address the problem, purpose of this paper).

- The tools and examples of using them and how they perform, results of using them.
- General discussion of how they add to the current literature / comparison to tools that are currently available.

Expanding on the last point, how do these calculators advance the field? Have the authors done their homework to see what is currently available? If there is nothing available that addresses these study design questions, then state it. If there is something available, it would help to compare the calculators presented in this paper to what currently exists, and argue why the presented calculators are better. For example, a VERY cursory online survey of currently available sample size calculators resulted in the following (many of which ignore within-person variability):

- EpiInfo – StatCalc from the CDC. https://cdc.gov/epiinfo/user-guide/statcalc/statcalcintro.html.
- Open Epi. https://openepi.com/SampleSize/SSCC.htm.
- Epi Tools. https://epitools.ausvet.com.au/samplesize.
- WinPEPI (PEPI for Windows). http://brixtonhealth.com/pepi4windows.html.
- Sample Size Calculator from CliniCalc. https://clinicalc.com/stats/samplesize.
- Sample Size Calculator from sample-size.net. https://sample-size.net.
- Sample Size for Repeated Measures ANOVA. https://scalestatistics.com/sample-size-for-repeated-measures-anova.html.
- Sample size packages or procedures in the major statistical packages available, such as R or SAS.

SPECIFIC COMMENTS

1. INTRODUCTION

Sections 1.2, 1.3, and 1.4 all seem to say similar things; this seems redundant and could be made more concise. As a specific example, all background and description of ICC could be combined and presented in one place near the beginning.

Section 2.3. Example 3: Longitudinal Studies on Chronic Disease Progression. “The ICC becomes particularly relevant in these studies, as it reflects the consistency of biomarker measurements within individuals across multiple time points, if we assume that there is no time-trend in the studied exposure.” As written, this seems odd – it is precisely the within-individual variation and the time trend that is of interest to study in chronic disease progression, so the between-individual variation and hence the ICC is of lower concern.

SECTION 2. MOTIVATING EXAMPLES AND DATA

What is the benefit of presenting Section 2 – Motivating Examples and Data? It doesn’t seem to relate to the rest of the paper. If the authors used those data to illustrate the calculators they present, that should be explicitly stated. Otherwise, there seems to be little reason to include this section at all; I suggest deleting it.

If the authors feel strongly about including Section 2, perhaps compress it to a short paragraph placed in the Introduction. Alternatively, if the authors truly want to keep it in the paper at this level of detail, this section would be simpler to follow if it was rearranged, such that each background example (e.g., section 2.1, Example 1, Assessing the Impact of Air Pollution on Respiratory Health) was immediately followed by the data example (e.g. section 2.5, Data Example 1: EPA Air Quality Monitoring Data).

SECTION 3. METHODS

Are the parameters for the examples in this section drawn from actual data? (E.g. EPA data on air pollution)? If so, it would be helpful to state that clearly for each example. Otherwise, readers could assume data are all hypothetical and thus perhaps of less relevance.

Sections 3 and 4. It might help to add a paragraph about how the calculators presented in this paper relate to each other, or a suggested order for use by a researcher designing a study from scratch.

SECTION 4. RESULTS

If the authors are able to arrange the paper as they like, I recommend rearranging the Methods and Results sections to put the Results immediately after the corresponding Methods. Example: Start with 3.1 – Calculator #1: The Number of Repeats Calculator, followed immediately by what is currently in 4.1: Number of Repeats Needed for Desired Validity Coefficient. I would move Figure 2 immediately after 4.1, so the reader doesn't need to flip back and forth between the Figure and the text that describes it.

Many if not most of the Results are intuitively obvious and do not add to the literature; some readers may wonder why their time is being wasted. Example in 4.1: "The number of repeats needed decrease as the ICC increases" or in 4.2: "When we need more precise estimates and exposure is more variable one can anticipate more resource-intensive research, far more so than if there was not intrinsic variability over time within a person." However, this can be acknowledged and used to bolster the validity and hence usefulness of the calculators, by adding something like the following sentence when the first results are presented: "The calculators yielded results that confirm what we would expect, indicating validity and utility in the field. Several specific results are listed below…"

SECTION 5. DISCUSSION

Neither section 5.1 or 5.2 seem to add anything really new to the literature. I suggest combining and compressing them.

What would really help is a discussion of how these calculators compare with already existing calculators, and thereby add to what is already known or available.

In the Discussion, it could be helpful to expand on practical aspects of using these tools, considering additional approaches in study design. For example:

- Recommendations to lower intra-individual variability, such as to use cumulative 24-hour voids in studies using urine samples, taking spot samples at the same time each day for compounds that show marked circadian patterns, or taking spot samples after fasting overnight.
- Recommendations to increase between-individual variability, such as selecting subjects with a large contrast in individual exposures (mentioned in Section 6.4).
- If an exposure of interest results in several metabolic products, choose the one with higher ICC (as long as it is a valid measure of exposure). Or if an exposure of interest includes several different compounds, focus on those with longer half-lives or other desirable biological characteristics (such as excreted in saliva or urine instead of requiring repeated blood draws).

Some of these tools require inputting parameter values that researchers might not be sure of for the

> compound(s) of interest, or that might not be readily available. Some potential solutions you might suggest in this white paper:
>
> - Plan for an initial small pilot study such as the one by Preau and Calafat, to generate all necessary parameters for your exposure(s) of interest. This has the added advantage of using your target study population under your laboratory procedures, as well as further specifically training your lab and clinical staff.
> - Estimate what likely maximum and minimum values might be for a given parameter, and plug those into the tools. The resulting answers should provide a range of maximum and minimum sample sizes needed to obtain desired results.
> - Encourage researchers to publish results of their biomonitoring studies that include the relevant parameters (between- and within-subject variability, etc). If not directly relevant to their study question, the values of these parameters could be in appendices. Or perhaps a database containing parameter values could be constructed and made publicly available, to which researchers could contribute as studies accumulate. If such a database already exists in some form, direct readers to it.

Expert 8

> Any models with time (longitudinal data, time to event,).
> e.g. cox models are very common in my field.

Expert 5

> The ability to include additional independent variables (that are correlated with the exposure variable of interest) in the calculators would reflect real word exposure scenarios more appropriately.

Comments (3)

SCORE **2** **Expert 3** *09/08/2024 16:10*

I really appreciate Expert 9's suggestions and would like to expand on the ideas provided in Expert 9's discussion comment. Could the calculators offer direct, actionable recommendations or additional outputs beyond just sample size? For example, could they suggest increasing between-individual variability as a recommendation?

SCORE **3** **Expert 6** *09/10/2024 13:57*

I suggest greater discussion of options beyond increase in number of exposure measurements per individual or increase in number of individuals when intra-individual exposure variability is high. Standardizing timing of samples during the day or by season is mentioned, but a simpler approach is to compare outcomes in groups of individuals known to have very high vs very low exposures by virtue of their occupations, residence, etc.

SCORE **0** **Expert 7** *09/13/2024 01:39*

Explicitly addressing likely missing data

Result 3.8 *(ID: 6552)*
Question 3.8 *(ID: 5904)*

Are there any other epidemiology study types or scenarios that you think that similar calculators would be useful for?

Expert 3

> Any study design except randomized trials would benefit from these calculators. Randomized trials break the link between exposure and outcome.

Expert 7

> Similar calculators would be useful for innovative designs – eg oversampling on high environmental exposure, and then increasing number of bio measurements for highly exposed?

Expert 4

> For Calculator 1, would it be able for the users to determine the optimal combination of m and n by inputting the cost for each measurement and cost for each participant recruited?

Expert 2

> 1. Although the calculator doesn't specify a particular type of epidemiological study design, I believe it can be broadly applied to population-based observational study designs, including cohort studies and retrospective cohort designs. Cross-sectional study designs may also benefit from the use of the calculators introduced in the white paper.
> 2. For case-control study designs, where biomonitoring for exposure assessment is based on historical records, a similar type of calculator could be highly useful.
> 3. Additionally, for more advanced study designs, such as those involving stratified or clustered randomization, multilevel data, or Mendelian randomization, similar calculators could also be beneficial.

Expert 6

> 1. Cluster-randomized trials
> 2. Within-person variability in outcome measurements
> 3. Meta-analysis across multiple studies

Expert 1

> Yes, I think more general sample size calculators for RCT, Case-control studies, Cohort studies etc... I realize that there are a lot already out there, but if I had a single website I could go to and do multiple calculations on a single site, it would make life easier but importantly, if the site was professionally developed, I would trust the output I obtained from such an online resource.

Expert 9

> Calculators like this might be useful for any epidemiology studies that collect multiple measures on individuals. Some potential examples follow:
> - Behavioral epidemiology (epi) studies or educational studies where several measures are taken

> on each individual, such as each subject responding to the same question over time, or each subject being rated by different evaluators;
> - Clinical epi studies with multiple evaluations of the same person. Lab studies are included as biomarkers. But other studies may have the same individual evaluated by different clinicians regarding diagnosis, severity of illness, etc.
> - Non-biomarker occupational or environmental epi studies where individuals self-report exposures at different times.

Expert 8

> I would say these are not only specific for 'biomonitoring' - maybe change the terminology

Expert 5

> Your group could likely create a calculator for categorical data - i.e. using kappa vs intraclass correlation.
>
> Also, often in environmental epidemiology studies, including biomonitoring studies, continuous exposures are categorized into ordinal categories for data analysis, and depending on the shape of the exposure distribution (i.e. skewed), this can introduce additional error that can result in unpredictable bias in different directions. It would be interesting to see the effect of categorization on the bias estimates in the presence of exposure measurement error.

Comments (2)

SCORE 0 **Expert 4** *09/03/2024 12:03*

About Expert 1's comments on sample size calculator for RCTs, I am not aware of any researchers that incorporate measurement error when estimating the sample size for RCT, so more work has to be done among trial statisticians before this could happen.

SCORE 0 **Expert 3** *09/08/2024 16:13*

Expert 4 has an excellent idea: include costs.

Result 4.1 *(ID: 6584)*
Question 4.1 *(ID: 5969)*

How important is it that researchers account for variability between- and within-persons in exposure to help guide design of epidemiology studies (beyond just "thinking about it")? (please explain).

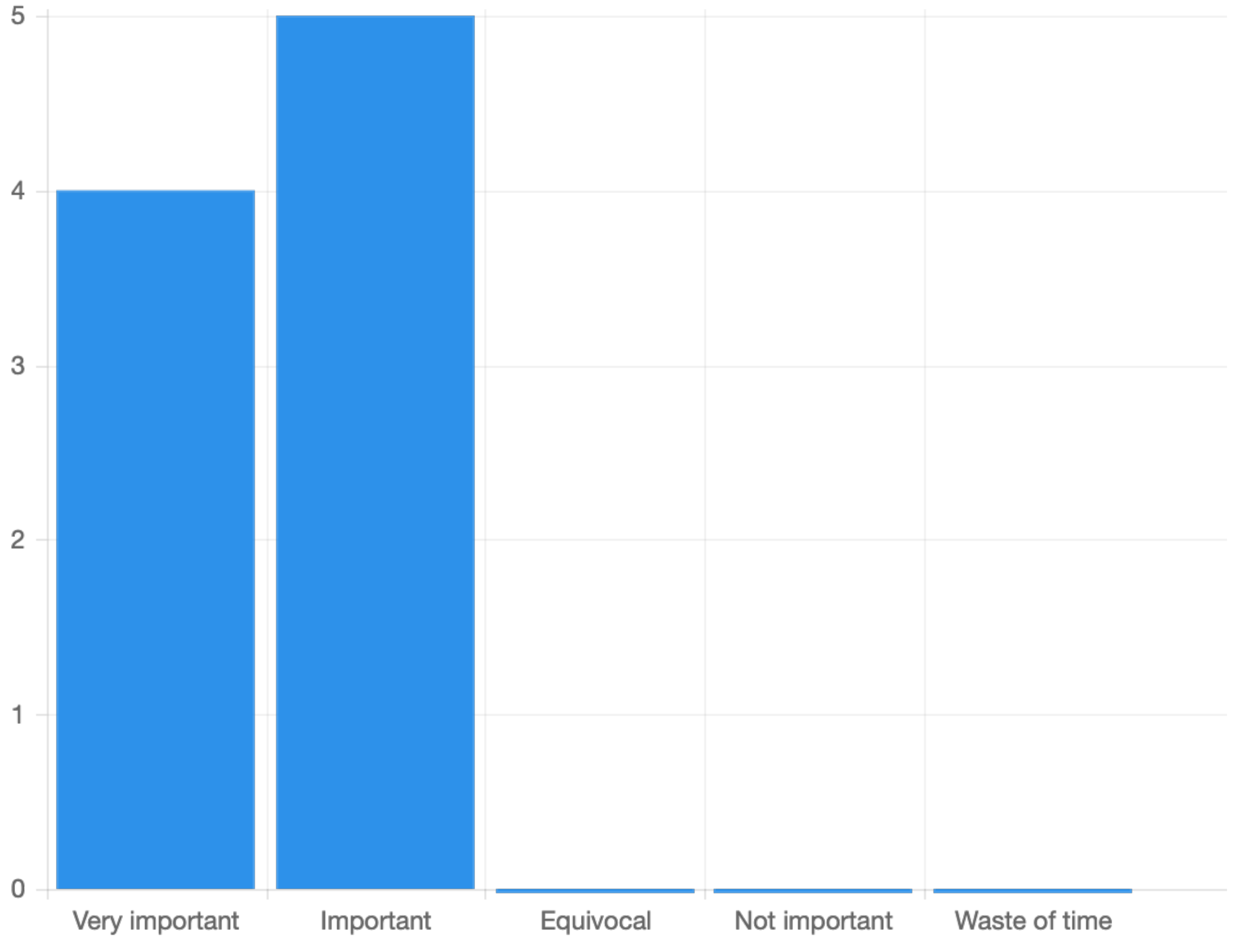

Legend

answers: 9
skips: 0

Answer Explanations

Expert 3 Explanation
Selected Answer(s): Very important

Between- and within-person exposure variability can result in exposure misclassification, which in turn attenuates the association between exposure and outcome. If not accounted for, this variability introduces noise into the data, weakening the study's conclusions.

Expert 2 Explanation
Selected Answer(s): Important

It is important for researchers to account for both between- and within-person variability in exposure when designing epidemiology studies, as it significantly impacts the accuracy and reliability of the study outcomes. Ignoring this variability can lead to exposure misclassification, resulting in biased estimates and misleading conclusions about exposure-response relationships. Properly addressing variability enhances statistical power by ensuring the study is neither under- nor overpowered, optimizing sample size and the number of measurements needed. Adjusting for within-person fluctuations helps capture true long-term associations, improving the precision of effect estimates and reducing bias. This approach is vital for deriving accurate, reliable conclusions that can inform public health interventions and policy decisions. By actively modeling variability rather than simply acknowledging it, researchers can better understand the true effects of exposure and design more valid, actionable studies.

However, the importance of accounting for between- and within-person variability depends on the type of epidemiological study design. Epidemiology studies are diverse, and in some cases, it may be difficult or impractical to account for this variability. For example, cross-sectional or retrospective designs often rely on single-point or historical measurements, limiting the ability to capture temporal changes. Case-control studies based on historical data may also lack the flexibility to track exposure variability over time. While addressing variability is critical in many longitudinal or cohort studies, its consideration must be balanced against the practical constraints of the chosen study design.

Expert 7 Explanation

Selected Answer(s): Important

It is important to think about both within and between variabilities in the design of the sutdies.
More over, sample size calculations are often required to obtain funding. How ever, such calculations rarely are true drivers of study design. Usually, the sample siaze is driven by the availability of data and financial constrains.

Expert 1 Explanation

Selected Answer(s): Very important

Obviously, this is dependent on the study design and aim in terms of biomonitoring of subjects. For many studies the most important exposure may be the comparison between subjects or within subjects. Therefore, the ability to accurately assess the variation either between or within subjects is critical to the success of the study.

Expert 9 Explanation

Selected Answer(s): Important

It is important to consider between- and within-person variability in exposure in order to increase the precision of results, and minimize exposure misclassification and thus biased estimates. However, this importance can vary with study design and practical concerns. Also, one of the benefits of using biomarkers of exposure is that many tend to integrate exposures over a period of time (which can vary with type of exposure, physiology, and sample medium); the resulting within-person variability may be lower than it would be by measuring exposure without using biomarkers.

Expert 8 Explanation

Selected Answer(s): Important

Important, to evaluate if performing a study is worth it in the first place; and to determine sample size, choose appropriate measurement methods, whether repeated measurements or continuous monitoring are needed, etc. to be able to detect a hypothesized effect; and choose appropriate statistical methods to account for e.g. fixed and

random effects, time-varying variables, etc.; and subsequently interpretation of results.

Expert 5 Explanation

Selected Answer(s): Very important

This must be considered in epidemiologic study design. Our students/researchers need more training and a better understanding of exposure measurement error to design and conduct studies that are sufficiently powered to detect risks that may not otherwise be found using traditional exposure measurements and epidemiologic approaches (i.e. yes/no, low/medium/high).

Comments (1)

SCORE 0

Expert 4 09/30/2024 15:57

Within-person variability is almost accounted for in studies with more than one observations from a person (e.g., repeated measures studies), but between-person variability is often ignored, partly due to the lack of historical data in ICC of the study population, and the other part due to lack of statistical tools for study design.

Result 4.2 *(ID: 6585)*
Question 4.2 *(ID: 5970)*

How important is it that researchers account for variability between- and within-persons in biomonitoring levels to help guide design of epidemiology studies (beyond just “thinking about it”)? (please explain).

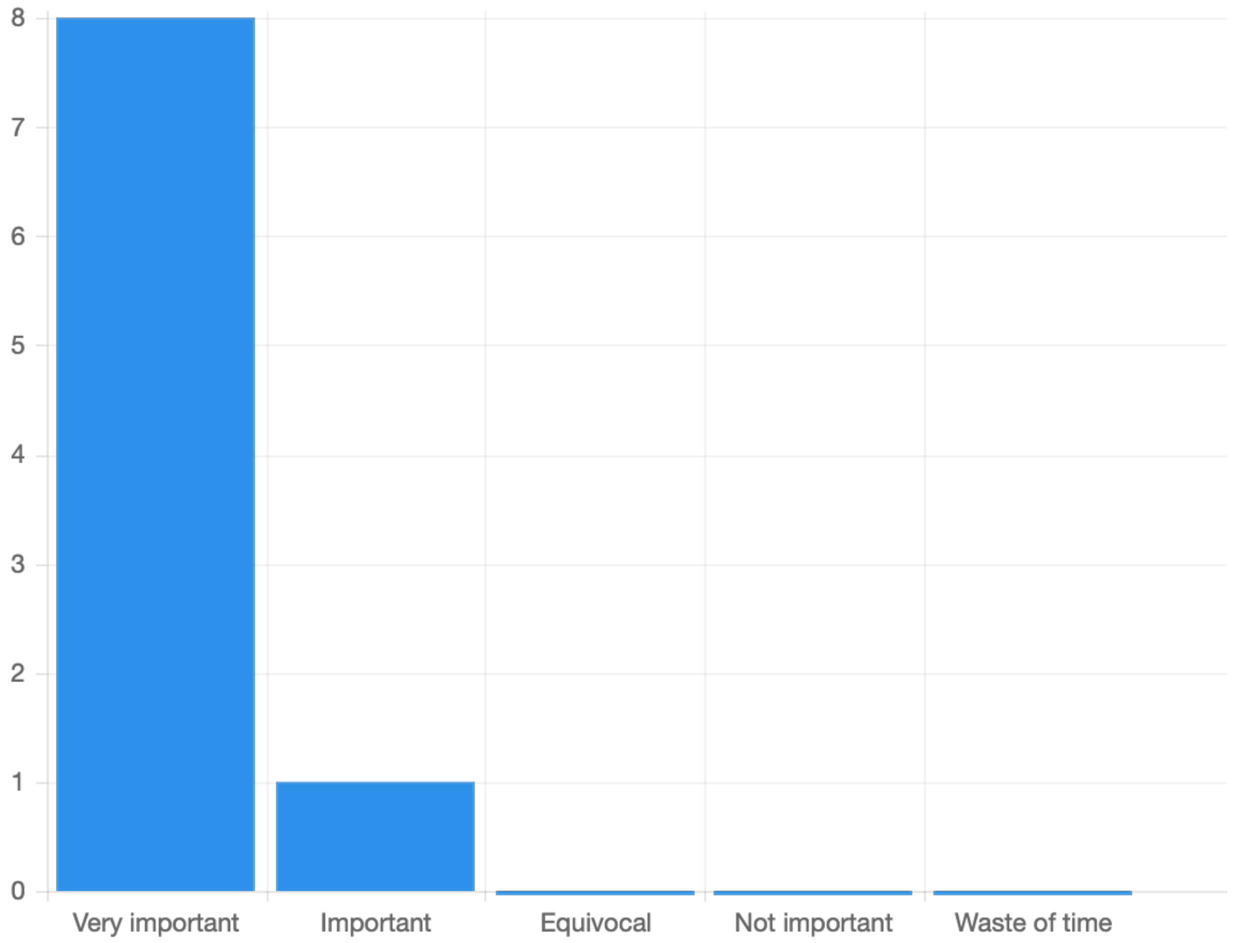

Legend

answers: 9
skips: 0

Answer Explanations

Expert 3 Explanation
Selected Answer(s): Very important

Between- and within-person biomarker variability can result in exposure misclassification, which in turn attenuates the association between exposure and outcome. If not accounted for, this variability introduces noise into the data, weakening the study's conclusions.

Expert 2 Explanation
Selected Answer(s): Very important

It is crucial for researchers to account for both between- and within-person variability in biomonitoring levels when designing epidemiology studies, as biomonitoring measures the actual internal dose of chemicals or

pollutants in the body, offering a more accurate reflection of real exposure than external exposure measurements alone. Properly addressing this variability is essential for optimizing sample size and the number of repeated measurements needed to detect true associations both efficiently and economically. Additionally, accounting for between-person variability improves the precision of effect estimates, leading to more reliable and valid study outcomes.

By actively modeling both types of variability, researchers can more accurately measure internal exposure and design studies that yield more robust and actionable results. This is especially critical in biomonitoring, where accurate internal dose measurements are key to understanding the health impacts of environmental exposures.

Expert 7 Explanation
Selected Answer(s): Very important

Answer to 4.2 applies here as well. The main difference is that with biomonitoring studies one can compare designs that include more subjects vs more repeated measurements,

---

Expert 1 Explanation
Selected Answer(s): Very important

Every study has a rate limiting step that either limits the number of subjects in a study or the number of assessments to be conducted on subjects as part of a biomonitoring study. The ability to be able to determine the "optimal" study given these logistical issues is very important in order to get more bang for your buck!

Expert 9 Explanation
Selected Answer(s): Very important

Designing a study to account for both between- and within-person variability in biomonitoring levels (if estimates are available or can be generated in a pilot study), will help minimize misclassification and bias in estimates of association. It will also help in getting the best balance between sample size and number of repeated measures for the available budget (and may help in securing funding to carry out the project). It is so important in fact, that authors should be encouraged to consistently report values of between- and within-person variability in results sections or appendices of manuscripts, to help in design and evaluation of future studies.

Expert 8 Explanation
Selected Answer(s): Very important

Same as above, but maybe even more important here because of the dynamic nature of many biomarkers (of course highly biomarker dependent!).

Expert 5 Explanation
Selected Answer(s): Very important

Same answer as above, expect we can hope to achieve more accurate measures of exposure using biomonitoring if the biosampling is correctly designed, and the toxicokinetics/dynamics are known.

Comments (1)

SCORE 0 **Expert 4** *09/30/2024 15:59*

Within-person variability is usually accounted for and has a larger magnitude than between-person variability, but still between-person variability should be accounted for, as it can cause

an underestimation of the sample size required.

Result 4.3 *(ID: 6586)*
Question 4.3 *(ID: 5971)*

In studies that measure or assess exposure for everyone, between- and within-persons variability in either exposure or biomarker of exposure play an important role in influencing power and bias. However, there is a competing approach (group-based exposure assessment, semi-ecological design) in which persons are grouped based on similar exposures and exposure typical of the group is assigned to all members of that group (e.g. based on residency, occupation). In this approach, between-group variability is involved in affecting power and bias. How important is it that researchers assess the power and bias of this group-based approach to help guide design of epidemiology studies that rely on biomarkers? (please explain)

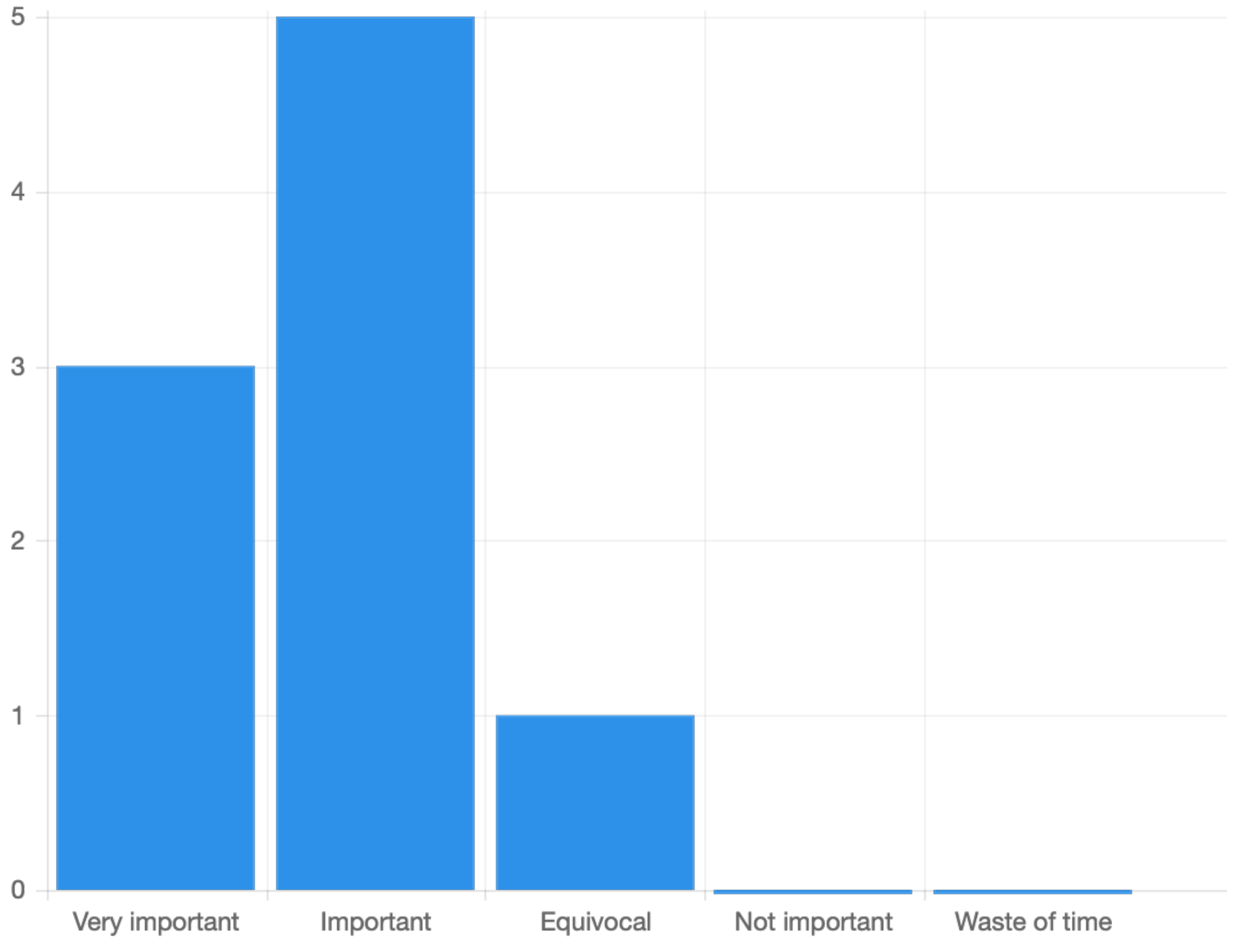

Legend

answers: 9
skips: 0

Answer Explanations

Expert 3 Explanation
Selected Answer(s): Very important

The statement is somewhat misleading. Similar exposure groups need to be validated using statistical tests, as

grouping categories such as occupation alone are not always reliable predictors. The statement also underestimates the purpose of group-based exposure assessment, which is not a competing approach but rather an extension of exposure assessment strategies. The key idea behind grouping is that more samples/measurements can be combined (and assigned to a subject), increasing the sample size (N) and thereby reducing noise while increasing the signal-to-noise ratio (SNR), as described by the formula SNR=mean/(SD/sqrt(N)) , where N is the sample size and SD is the standard deviation of noise.

Grouping strategies are effective when within-group variability is small and between-group variability is large (which creates more contrast). For example, a geometric standard deviation (GSD) of 2 (though some research suggests GSDs of up to 4 are acceptable) is typically considered low enough variance to justify grouping subjects. If the GSD is small, a grouping strategy can help reduce attenuation in the association between exposure and outcome. In all strategies, the attenuation of the association needs to be calculated to compare strategies.
lambda = vb/(vb+vw/n)
where lambda = attenuation, vb = **between-subject** OR **between-group** vw = **within-subject** OR **within-group AND n = number of repeated measurements** per subject or **subjects per group.**

Therefore, both between- and within-group variance must be assessed for the exposure, the biomarker, and the exposure assessment strategies.

Once this assessment is complete, the best exposure strategy can be used to calculate the study's power and bias, and to determine the appropriate sample size.

Expert 2 Explanation

Selected Answer(s): Very important

It is very important for researchers to assess the power and bias of the group-based exposure approach in epidemiology studies, especially when individual-level variability is unavailable. In this approach, individuals are assigned a common exposure level, which simplifies assessment but presents significant challenges by overlooking individual variability. This can lead to exposure misclassification, resulting in biased or attenuated effect estimates. The success of the group-based approach relies on sufficient between-group variability; without well-differentiated groups, statistical power is reduced, making it harder to detect true associations. Additionally, ecological bias may arise, where group-level exposure does not accurately reflect individual outcomes. When using individual-level biomarker data, further complications can occur, as biomarkers reflect internal doses that may vary within groups and be missed by group-based assessments. Therefore, evaluating power and bias is critical, particularly in the absence of individual variability, to ensure the study design is robust and produces reliable, valid results. This assessment ensures researchers sufficiently account for variability in exposure, optimizing the ability to detect meaningful associations while minimizing bias.

Expert 6 Explanation

Selected Answer(s): Important

This approach can be very useful if repeated exposure measurements in individual participants is infeasible and within-person variability in such measurements is known to be high. Comparing outcomes in groups known to be at high environmental exposure (e.g., by virtue of occupation, residence, or accidental contamination) vs no or

very low exposure can provide an efficient and unbiased design. Dose-response relationships will not be possible with such a design, however, unless one or more intermediate exposure groups are also compared.

Expert 7 Explanation

Selected Answer(s): Very important

It is important to evaluate potential bias in all studies (not just one that include biomarkers.
I do not agree with a premise that exposure based on residency or occupation are nesseserily of semi-ecological design and/or that such studies are more prone to bias than biomonitoring studies.
Biomonitoring studies rely on a different set of assumptions and can be biased as well. For example, missing data can be selective and lead to bias, or timing of monitoring can lead to bias, or be not representative of exposures of interest for a variety of reasons.

Expert 1 Explanation

Selected Answer(s): Important

There is likely to be confounding factors between groups and these should be determined in as much detail as possible so that any potential bias can be accounted for in each group. When all things have been considered and variation exists, it can be seen to be a true variation between groups. The assessment of potential confounding factors is complex and difficult and also limited by other factors such as sample size when conducting analysis. In reality it is not always possible to account for all bias factors in a study, but the study that attempts to do this will have the better study and outcomes.

Expert 9 Explanation

Selected Answer(s): Important

This is important if the group-based approach is both relevant to the study question and valid, and if the groups can be reliably distinguished. Also, sometimes it might be the most practical solution if individual-level data are not available or impractical to obtain. However, the lack of data on individuals may lead to misclassification and bias if there is significant variability within the groups, so it would be helpful to assess the power and bias of this approach. In searching for groups with sufficiently contrasting exposures, it could also be helpful to consider selection bias and representativeness of the resulting study population.

Expert 8 Explanation

Selected Answer(s): Important

It's important to take into account as this may indeed affect power and thereby decisions on sample size. Group-level exposure = lower variability: this may either increase power by reducing noise, but also decrease it by reducing overall contrasts.
In terms of bias, there is a risk of ecological fallacy and of course exposure misclassification. Confounding factors may also be different, they won't all be similar at the same grouping level, it may be good to also measure and analyze these at an individual level.

Expert 5 Explanation

Selected Answer(s): Equivocal

I wouldn't necessarily call this a semi-ecological design. In occupational studies, workers may be classified by occupation, industry, job title, tasks performed etc. but we recognize that their exposures within the group will be different. We assume that the exposures between the groups will show more variability. This is often not the case (and why we don't often find significant results), but I'm not aware of many studies that can quantify these

relationships (variations within groups/between groups) expect for some early work done by Kromhout et al.

For some basic info see: Mannetje A', Kromhout H. The use of occupation and industry classifications in general population studies. Int J Epidemiol. 2003 Jun;32(3):419-28. doi: 10.1093/ije/dyg080. PMID: 12777430.

Expert 4 Explanation

Selected Answer(s): Important

If group-based approach is used, the between-individual variability would be ignored.

Result 4.4 *(ID: 6587)*
Question 4.4 *(ID: 5972)*

How important would you consider the following practices for the design of epidemiology studies that rely on biomonitoring for exposure assessments? (rate from not at all important (1) to very important (5) on visual analogue scale).

	1	2	3	4	5	Total
Conduct a pilot study to estimate variance components of the biomarker of exposure (when not already known)	**0.00%** 0	**11.11%** 1	**0.00%** 0	**22.22%** 2	**66.67%** 6	9
Identify a hypothesized minimal effect size (in the outcomes measure) that the researcher intends to be able to identify	**0.00%** 0	**22.22%** 2	**0.00%** 0	**0.00%** 0	**77.78%** 7	9
Estimate the impact on bias and power of measurement error in outcome, i.e. within-person variability in outcome	**0.00%** 0	**11.11%** 1	**0.00%** 0	**55.56%** 5	**33.33%** 3	9
For a given exposure of interest, select biomarkers with the largest intraclass correlation coefficient (ICC) (e.g., select biomarkers with the longest half-life)	**0.00%** 0	**0.00%** 0	**22.22%** 2	**66.67%** 6	**11.11%** 1	9
Consider study designs that do not rely only on contrasts in exposures among individuals but instead seek groups of individuals with very different exposures	**0.00%** 0	**0.00%** 0	**22.22%** 2	**55.56%** 5	**22.22%** 2	9
Articulate the hypothesized causal diagram (DAG) that includes variability in biomarkers of exposure between- and within-persons	**0.00%** 0	**11.11%** 1	**44.44%** 4	**22.22%** 2	**22.22%** 2	9
Calculate the efficiency, in terms of costs, of investing into more measurements within person versus more persons to minimize bias (as was done by Armstrong (1996))	**0.00%** 0	**11.11%** 1	**22.22%** 2	**44.44%** 4	**22.22%** 2	9
Utilize pools of biofluids from each individual to help minimize within-person variability and increase ICC	**11.11%** 1	**22.22%** 2	**44.44%** 4	**22.22%** 2	**0.00%** 0	9
Other ___________(please specify)	**0.00%** 0	**0.00%** 0	**0.00%** 0	**0.00%** 0	**100.00%** 3	3

Answer Explanations

Expert 3 Explanation
Selected Answer(s):

	1	2	3	4	5
Conduct a pilot study to estimate variance components of the biomarker of exposure (when not already known)	0	0	0	0	1
Identify a hypothesized minimal effect size (in the outcomes measure) that the researcher intends to be able to identify	0	0	0	0	1
Estimate the impact on bias and power of measurement error in outcome, i.e. within-person variability in outcome	0	0	0	0	1
For a given exposure of interest, select biomarkers with the largest intraclass correlation coefficient (ICC) (e.g., select biomarkers with the longest half-life)	0	0	0	0	1

	1	2	3	4	5
Consider study designs that do not rely only on contrasts in exposures among individuals but instead seek groups of individuals with very different exposures	0	0	0	0	1
Articulate the hypothesized causal diagram (DAG) that includes variability in biomarkers of exposure between- and within-persons	0	0	0	0	1
Calculate the efficiency, in terms of costs, of investing into more measurements within person versus more persons to minimize bias (as was done by Armstrong (1996))	0	1	0	0	0
Utilize pools of biofluids from each individual to help minimize within-person variability and increase ICC	1	0	0	0	0
Other ___________(please specify)	0	0	0	0	1

Calculate the attenuation for different exposure strategies to identify and select the most effective exposure strategy.

Utilizing pooled biofluids from each individual to minimize within-person variability and increase the ICC sounds like a poor strategy, as important signals could be lost. Instead, the variability should be calculated and used in the assessment. An epidemiological study should be optimized for the strength of the association between the predictor and the outcome. Focusing on financial efficiency too early could lead to poor decisions. Whether more samples per person or more participants are needed should be based solely on variability. Once the optimal strategy is selected, cost efficiency can be calculated, but not beforehand.

Expert 2 Explanation
Selected Answer(s):

	1	2	3	4	5
Conduct a pilot study to estimate variance components of the biomarker of exposure (when not already known)	0	0	0	0	1
Identify a hypothesized minimal effect size (in the outcomes measure) that the researcher intends to be able to identify	0	0	0	0	1
Estimate the impact on bias and power of measurement error in outcome, i.e. within-person variability in outcome	0	0	0	0	1
For a given exposure of interest, select biomarkers with the largest intraclass correlation coefficient (ICC) (e.g., select biomarkers with the longest half-life)	0	0	0	1	0
Consider study designs that do not rely only on contrasts in exposures among individuals but instead seek groups of individuals with very different exposures	0	0	0	1	0
Articulate the hypothesized causal diagram (DAG) that includes variability in biomarkers of exposure between- and within-persons	0	0	1	0	0
Calculate the efficiency, in terms of costs, of investing into more measurements within person versus more persons to minimize bias (as was done by Armstrong (1996))	0	0	1	0	0
Utilize pools of biofluids from each individual to help minimize within-person variability and increase ICC	0	1	0	0	0
Other ___________(please specify)					

The most important practices in designing epidemiology studies that rely on biomonitoring for exposure assessments include conducting a pilot study to estimate variance components of the biomarker, identifying a hypothesized minimal effect size, and estimating the impact of measurement error, as these directly influence the study's reliability, power, and ability to detect meaningful associations. Selecting biomarkers with high intraclass correlation coefficients (ICC) and considering study designs that focus on groups with highly contrasting exposures are also important for reducing variability and increasing the robustness of findings. Articulating a hypothesized causal diagram (DAG) is useful for clarifying assumptions and identifying confounders. Calculating the efficiency of additional within-person versus more-person measurements is important for cost-effective study design, while utilizing pooled biofluids, though beneficial for reducing variability, is less universally applicable but still useful in certain contexts.

Expert 6 Explanation
Selected Answer(s):

	1	2	3	4	5
Conduct a pilot study to estimate variance components of the biomarker of exposure (when not already known)	0	0	0	0	1
Identify a hypothesized minimal effect size (in the outcomes measure) that the researcher intends to be able to identify	0	0	0	0	1
Estimate the impact on bias and power of measurement error in outcome, i.e. within-person variability in outcome	0	0	0	1	0
For a given exposure of interest, select biomarkers with the largest intraclass correlation coefficient (ICC) (e.g., select biomarkers with the longest half-life)	0	0	0	1	0
Consider study designs that do not rely only on contrasts in exposures among individuals but instead seek groups of individuals with very different exposures	0	0	0	1	0
Articulate the hypothesized causal diagram (DAG) that includes variability in biomarkers of exposure between- and within-persons	0	1	0	0	0
Calculate the efficiency, in terms of costs, of investing into more measurements within person versus more persons to minimize bias (as was done by Armstrong (1996))	0	0	0	1	0
Utilize pools of biofluids from each individual to help minimize within-person variability and increase ICC	0	0	0	1	0
Other ___________(please specify)	0	0	0	0	1

Using biosamples that reflect long-term exposure, such as urine, hair, or nails can provide time-averaged exposures that can either replace or supplement single-sample blood or saliva samples. Another strategy is to include standardized timing of sampling, if single.

Expert 9 Explanation
Selected Answer(s):

	1	2	3	4	5
Conduct a pilot study to estimate variance components of the biomarker of exposure (when not already known)	0	0	0	1	0
Identify a hypothesized minimal effect size (in the outcomes measure) that the researcher intends to be able to identify	0	0	0	0	1
Estimate the impact on bias and power of measurement error in outcome, i.e. within-person variability in outcome	0	0	0	1	0
For a given exposure of interest, select biomarkers with the largest intraclass correlation coefficient (ICC) (e.g., select biomarkers with the longest half-life)	0	0	0	1	0
Consider study designs that do not rely only on contrasts in exposures among individuals but instead seek groups of individuals with very different exposures	0	0	0	1	0
Articulate the hypothesized causal diagram (DAG) that includes variability in biomarkers of exposure between- and within-persons	0	0	1	0	0
Calculate the efficiency, in terms of costs, of investing into more measurements within person versus more persons to minimize bias (as was done by Armstrong (1996))	0	0	0	1	0
Utilize pools of biofluids from each individual to help minimize within-person variability and increase ICC	0	0	1	0	0
Other ___________(please specify)	0	0	0	0	1

A pilot study is recommended if it is feasible to conduct. Not only will it give an indication of between- and within-person variability, but will also help train staff in sample collection and analysis (particularly if they have little experience with the samples involved).
It's helpful to select biomarkers with the largest ICC, but balanced with considerations if those biomarkers are relevant to the study question and if they are practical to collect. For example, blood samples might have the largest ICC, but it might be more practical to collect saliva or urine samples.
Groups of individuals with very different exposures can be helpful when individual measurements are less feasible. However, (a) the researcher must be aware that there may be variability between individuals within the

group that will be hidden, and (b) selection bias and representativeness of groups for the study question should be considered.
DAGs can be a useful thought experiment. However, sometimes I wonder if they might give us a false sense of security; do we truly know that level of detail regarding how all the variables relate to and interact with each other?
As well as the statistical considerations focused on in this project, the biomonitored samples must be valid measures of the exposure of interest; they must actually measure what the researcher wants them to. This includes considering biological aspects such as physiology and toxicology, and practical aspects like timing of collection, ease of collection, and likelihood of contamination.

Expert 8 Explanation
Selected Answer(s):

	1	2	3	4	5
Conduct a pilot study to estimate variance components of the biomarker of exposure (when not already known)	0	0	0	0	1
Identify a hypothesized minimal effect size (in the outcomes measure) that the researcher intends to be able to identify	0	0	0	0	1
Estimate the impact on bias and power of measurement error in outcome, i.e. within-person variability in outcome	0	0	0	1	0
For a given exposure of interest, select biomarkers with the largest intraclass correlation coefficient (ICC) (e.g., select biomarkers with the longest half-life)	0	0	0	1	0
Consider study designs that do not rely only on contrasts in exposures among individuals but instead seek groups of individuals with very different exposures	0	0	0	1	0
Articulate the hypothesized causal diagram (DAG) that includes variability in biomarkers of exposure between- and within-persons	0	0	0	0	1
Calculate the efficiency, in terms of costs, of investing into more measurements within person versus more persons to minimize bias (as was done by Armstrong (1996))	0	0	0	1	0
Utilize pools of biofluids from each individual to help minimize within-person variability and increase ICC	0	0	1	0	0
Other ___________(please specify)					

I think it's crucial to know the variability in biomarkers, so when not known from the literature, pilot studies are very important. Similarly, determining the minimal effect size is also very important. Both are essential for power calculations and determining appropriate sample sizes and sampling strategies - to avoid underpowered studies and/or a waste of resources of participant burden.

Bias in outcome measurement is of course also important, for choosing better methods where possible, and/or to take into account in adjusting analyses and interpreting results.

I'm a big fan of DAGs for understanding and communicating complex relationships between many variables (exposures, outcomes, covariates) and help guide analyses and interpretation.

Regarding pools of biofluids, I'm not sure. This may reduce withinperson variability but could also obscure e.g. differences in time or other changes?

Expert 5 Explanation
Selected Answer(s):

	1	2	3	4	5
Conduct a pilot study to estimate variance components of the biomarker of exposure (when not already known)	0	0	0	0	1
Identify a hypothesized minimal effect size (in the outcomes measure) that the researcher intends to be able to identify	0	0	0	0	1
Estimate the impact on bias and power of measurement error in outcome, i.e. within-person variability in outcome	0	0	0	0	1
For a given exposure of interest, select biomarkers with the largest intraclass correlation coefficient (ICC) (e.g., select biomarkers with the longest half-life)	0	0	1	0	0
Consider study designs that do not rely only on contrasts in exposures among individuals but instead seek groups of individuals with very different exposures	0	0	1	0	0
Articulate the hypothesized causal diagram (DAG) that includes variability in biomarkers of exposure between- and within-persons	0	0	1	0	0
Calculate the efficiency, in terms of costs, of investing into more measurements within person versus more persons to minimize bias (as was done by Armstrong (1996))	0	0	0	0	1
Utilize pools of biofluids from each individual to help minimize within-person variability and increase ICC	0	1	0	0	0
Other ___________(please specify)					

I would only recommend using pools of biofluids when it is necessary to combine samples because of laboratory analytical capabilities (i.e. they need a larger volume for appropriate limits of detection), or if multiple samples are collected from an individual and the resources ($) are not available to analyze the individual samples. It's better to understand both the intra and inter-individual variability when the samples are available.

Please also note that biomarkers with the longest half life may not be those with the largest ICC. They just reflect a subject's longer term exposure which is particularly useful for case-control studies. However, if all subjects are similarity exposed to a chemical with a long half-life, their between-subject variation may be minimal as well as the within.

Expert 4 Explanation

Selected Answer(s):

	1	2	3	4	5
Conduct a pilot study to estimate variance components of the biomarker of exposure (when not already known)	0	0	0	1	0
Identify a hypothesized minimal effect size (in the outcomes measure) that the researcher intends to be able to identify	0	0	0	0	1
Estimate the impact on bias and power of measurement error in outcome, i.e. within-person variability in outcome	0	0	0	1	0
For a given exposure of interest, select biomarkers with the largest intraclass correlation coefficient (ICC) (e.g., select biomarkers with the longest half-life)	0	0	0	1	0
Consider study designs that do not rely only on contrasts in exposures among individuals but instead seek groups of individuals with very different exposures	0	0	0	1	0
Articulate the hypothesized causal diagram (DAG) that includes variability in biomarkers of exposure between- and within-persons	0	0	0	1	0
Calculate the efficiency, in terms of costs, of investing into more measurements within person versus more persons to minimize bias (as was done by Armstrong (1996))	0	0	0	0	1
Utilize pools of biofluids from each individual to help minimize within-person variability and increase ICC	0	0	0	1	0
Other ___________(please specify)					

The idea of conducting a pilot study to estimate the variance components is interesting, but often involve a large cost as the variance components have a higher standard error than a mean / proportion.

Result 4.5 *(ID: 6588)*
Question 4.5 *(ID: 5973)*

How important would you consider the following practices for the analysis of epidemiology studies that rely on biomonitoring for exposure assessments? (rate from not at all important (1) to very important (5) on visual analogue scale)

	1	2	3	4	5	Total
Adjust for bias due to measurement error in exposure	**11.11%** 1	**0.00%** 0	**0.00%** 0	**22.22%** 2	**66.67%** 6	9
Adjust for bias due to measurement error in covariates	**0.00%** 0	**11.11%** 1	**22.22%** 2	**44.44%** 4	**22.22%** 2	9
Adjust for bias due to measurement error in outcome	**0.00%** 0	**11.11%** 1	**11.11%** 1	**11.11%** 1	**66.67%** 6	9
Adjust for multiple comparisons	**11.11%** 1	**11.11%** 1	**33.33%** 3	**22.22%** 2	**22.22%** 2	9
Quantitatively account for unmeasured confounding	**11.11%** 1	**0.00%** 0	**44.44%** 4	**33.33%** 3	**11.11%** 1	9
Employ Bayesian methods that quantitatively integrate prior knowledge on the topic	**0.00%** 0	**0.00%** 0	**44.44%** 4	**44.44%** 4	**11.11%** 1	9
Other ___________(please specify)	**0.00%** 0	**0.00%** 0	**33.33%** 1	**66.67%** 2	**0.00%** 0	3

Answer Explanations

Expert 3 Explanation
Selected Answer(s):

	1	2	3	4	5
Adjust for bias due to measurement error in exposure	0	0	0	0	1
Adjust for bias due to measurement error in covariates	0	0	0	0	1
Adjust for bias due to measurement error in outcome	0	0	0	0	1
Adjust for multiple comparisons	0	0	0	0	1
Quantitatively account for unmeasured confounding	0	0	0	0	1
Employ Bayesian methods that quantitatively integrate prior knowledge on the topic	0	0	0	0	1
Other ___________(please specify)					

All of these practices are very important and should be standard in epidemiological studies. Measurement bias, covariate bias, and measurement error bias can significantly alter (e.g., attenuate or distort) the associations between exposure and outcome. Unmeasured confounding can bias results, and Bayesian methods can enhance the robustness of the assessment by integrating prior knowledge, thereby strengthening the analysis of the associations between outcome and predictor.

Expert 2 Explanation
Selected Answer(s):

	1	2	3	4	5
Adjust for bias due to measurement error in exposure	0	0	0	0	1
Adjust for bias due to measurement error in covariates	0	0	0	1	0
Adjust for bias due to measurement error in outcome	0	0	0	0	1
Adjust for multiple comparisons	0	0	1	0	0
Quantitatively account for unmeasured confounding	0	0	0	1	0
Employ Bayesian methods that quantitatively integrate prior knowledge on the topic	0	0	0	1	0
Other ___________(please specify)	0	0	1	0	0

In the analysis of epidemiology studies using biomonitoring for exposure assessments, adjusting for bias due to measurement error in both exposure and outcome is critically important, as misclassification in these areas can significantly distort study results. Measurement error in covariates is also important to address, though its impact is generally less severe. Adjusting for multiple comparisons is necessary to prevent false positives, especially in studies with numerous tests. Quantitatively accounting for unmeasured confounding is crucial for producing valid causal inferences. While Bayesian methods that integrate prior knowledge can be beneficial, their importance varies depending on the study context and availability of reliable prior data.
It's also important to address several additional issues below:

- Handling missing data properly through methods like multiple imputation helps prevent bias.
- Selection bias must be considered to ensure the study population is representative.
- Non-linear relationships and interaction effects between exposures and covariates should be explored for a more accurate understanding of the data.
- Accounting for multiple sources of exposure and performing sensitivity analyses to test the robustness of assumptions are also essential for producing reliable and comprehensive results.

Expert 6 Explanation
Selected Answer(s):

	1	2	3	4	5
Adjust for bias due to measurement error in exposure	0	0	0	1	0
Adjust for bias due to measurement error in covariates	0	0	0	1	0
Adjust for bias due to measurement error in outcome	0	0	0	1	0
Adjust for multiple comparisons	0	0	0	1	0
Quantitatively account for unmeasured confounding	0	0	1	0	0
Employ Bayesian methods that quantitatively integrate prior knowledge on the topic	0	0	1	0	0
Other ___________(please specify)	0	0	0	1	0

Sensitivity analyses can be helpful to assess effects of metabolism, timing of sample, and other sources of within-person variability.

Expert 9 Explanation
Selected Answer(s):

	1	2	3	4	5
Adjust for bias due to measurement error in exposure	0	0	0	0	1

Adjust for bias due to measurement error in covariates	0	0	0	1	0
Adjust for bias due to measurement error in outcome	0	0	0	0	1
Adjust for multiple comparisons	0	0	1	0	0
Quantitatively account for unmeasured confounding	0	0	1	0	0
Employ Bayesian methods that quantitatively integrate prior knowledge on the topic	0	0	0	1	0
Other ___________(please specify)	0	0	0	1	0

Adjusting for bias due to measurement error in exposure and outcome seems to more directly address the primary study question(s), and probably more critical than measurement error in covariates.
Most of the time it is helpful to adjust for multiple comparisons. But researchers may find it helpful to consider other aspects in evaluating measures of association besides just statistical significance. For example, are there exciting new leads that just had too small a number of people exposed to be highly significant? Are there consistent findings between related biomarkers that might indicate something important is going on, or that might support interpretation of a common pathway?
Sensitivity analyses can be useful in interpretation, for example, by giving upper and lower bounds of likely results.

Expert 8 Explanation
Selected Answer(s):

	1	2	3	4	5
Adjust for bias due to measurement error in exposure	0	0	0	0	1
Adjust for bias due to measurement error in covariates	0	0	1	0	0
Adjust for bias due to measurement error in outcome	0	0	1	0	0
Adjust for multiple comparisons	0	0	0	1	0
Quantitatively account for unmeasured confounding	0	0	0	1	0
Employ Bayesian methods that quantitatively integrate prior knowledge on the topic	0	0	1	0	0
Other ___________(please specify)					

Adjusting for bias in exposure assessment - where possible - is very important to reduce bias in effect estimates. If non-differential, these errors typically result in attenuation bias and null findings where there may be true effects.

Adjusting for bias due to measurement errors in covariates and outcomes is also important but in my opinion to a lesser extent. For potential confounders there is of course a risk is residual confounding by poorly measured covariates. For outcomes, a main risk is reduced precision in effect estimates. But there are differences for binary versus continuous outcome variables.

Adjusting for multiple testing is important in any epidemiological study design to reduce the risk for false positive (chance) findings. Bonferroni correcation may be too strict/conservative, but other methods are available.

Expert 5 Explanation
Selected Answer(s):

	1	2	3	4	5
Adjust for bias due to measurement error in exposure	0	0	0	0	1
Adjust for bias due to measurement error in covariates	0	0	0	1	0
Adjust for bias due to measurement error in outcome	0	0	0	0	1
Adjust for multiple comparisons	0	1	0	0	0

Quantitatively account for unmeasured confounding	1	0	0	0	0
Employ Bayesian methods that quantitatively integrate prior knowledge on the topic	0	0	1	0	0
Other ___________(please specify)					

I think the adjustment for measurement error is always important regardless of whether the variable is an exposure, confounder, effect modifier or outcome. However, we generally don't have parameter estimates for these associations (i.e. correlations, ICCs etc.). More efforts in pilot studies and exposure validation studies would help to improve this situation, but generally these types of studies are difficult to get funded.

I'm not clear if you mean unmeasured or unknown confounding. If unmeasured (i.e. a smoking variable is not measured in an occupational lung cancer study), it would be very important to account for a smoking confounder by using a proxy (such as SES) or other techniques.

Result 4.6 *(ID: 6589)*
Question 4.6 *(ID: 5974)*

How useful do you think the calculators are to interpretation of published studies in terms of what they can tell us given the known or suspected degree of between- and within-person variance in biomarkers? Consider arguments of question 4.1. (please explain)

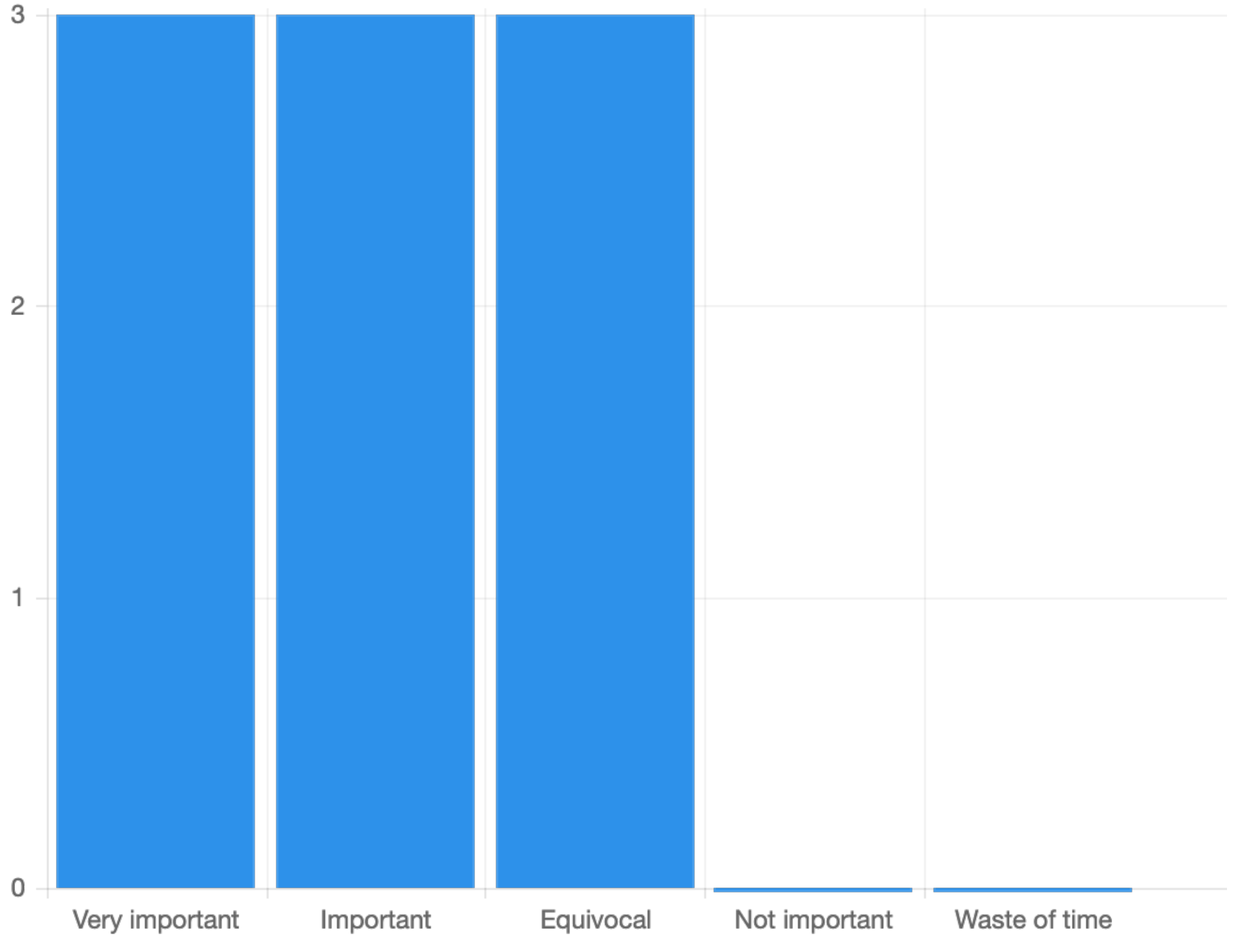

Legend

answers: 9
skips: 0

Answer Explanations

Expert 3 Explanation
Selected Answer(s): Very important

This is a very interesting idea. The calculators could be used to verify and review articles. However, the issue I see is that relevant information (e.g., variance components, raw data) is often not published or cannot be derived from the presented data. Additionally, the calculators are not complete. They need to account for at least the variabilities of biomarkers and exposure and should allow for the entry of parameter ranges.

Expert 2 Explanation
Selected Answer(s): Very important

The calculators are highly useful for interpreting published studies by enhancing the precision, validity, and generalizability of findings, because within-person variability can obscure true associations between exposure and outcomes. These tools may assess the impact of variability on statistical power, informing researchers about potential biases and the need for larger sample sizes or repeated measurements. Additionally, they assist in optimizing study design by guiding resource allocation and improving the reliability of exposure-response relationships, ultimately providing a clearer understanding of the true effects of exposure.

Expert 1 Explanation

Selected Answer(s): Very important

Retrospectively assessing published studies using the calculators would be extremely useful, especially if you were planning on conducting your own study and wanted to improve on the published study. Some of the factors in 4.4 and 4.5 might be of value if the scientist was able to enter basic information about the study and alternative options were provided that may improve on the published study.

Expert 9 Explanation

Selected Answer(s): Important

The calculators could be useful if the relevant information (between- and within-person variance) is made available by the authors. This is additional motivation for recommending that authors include this information in results or appendices; not only can it help evaluate the published study, but can also be useful for researchers designing future studies.

Expert 8 Explanation

Selected Answer(s): Equivocal

Depending on what it reported in the studies they can be useful.

Expert 5 Explanation

Selected Answer(s): Important

I think they are useful, especially for those researchers who do not have a background or understanding of these issues.

Expert 4 Explanation

Selected Answer(s): Equivocal

The calculators are for sample size estimation and may not be important to interpret results with known between- and within-person variance.

Result 4.7 *(ID: 6590)*
Question 4.7 *(ID: 5975)*

What do you think is the acceptable power of epidemiologic design, when type 1 error (p-value) is fixed at 0.05 (two-sided)? Please specify_____ %.

Expert 3

> Isn't there an "an" missing before "epidemiological design"?
> I think the question is ambiguous. Do I not need study design, effect size (d), sample size(n), and standard deviation (sd), too to answer the question? I assume a cross-sectional design and use the sample size equation to calculate power: Zb = sqrt((n*d^2)/(2*sd^2)). If n=100, delta = 0.3, sd = 1, =>
> Zb = 0.161 (hopefully)
> => Zb I am looking up = 56%

Expert 2

> I would suggest a power between 80% and 90% when the type 1 error (alpha level) is fixed at 0.05 (two-sided). This range strikes a balance between reducing the probability of a false negative (type 2 error) and maintaining a reasonable sample size. However, though this range is widely accepted in practice, it is a subjective choice, just like the type 1 error of 0.05. The acceptable power level depends on several factors, including the expected effect size, the importance of the outcome, the feasibility of recruiting a larger sample size, and the potential consequences of missing a true association. In high-stakes research, such as studies related to public health or clinical interventions, higher power (closer to 90%) may be preferred to ensure the findings are robust, while in exploratory or resource-constrained studies, lower power (around 80%) might be acceptable.

Expert 6

> 80% is fine, but more important is the effect size estimated. 80% is too low for a huge effect (e.g., a RR of 5) but perhaps too high for a small effect (RR = 1.1or 1.2). Observational studies are too crude and susceptible to bias to infer causal effects of very small magnitude.

Expert 7

> 90%

Expert 1

> Traditionally, the minimum Power of any epidemiological study is 80%. But given other limitations associated with the study design (e.g., costs, sample size) this is not always possible. In reality, I have only ever seen sample size calculations published for two studies, these studies achieved the required Power. Most Power calculations are used primarily for Grant applications to show that the required sample size is feasible, however, the majority of published studies never meet the required Power to even undertake analysis.

Expert 9

> Between 80% and 90%. I think this depends on several factors. For example, context of the study - is it a novel hypothesis where lower power might be acceptable, or have several similar studies preceded it, and it is trying to answer a question unequivocally where higher power is needed? What are the clinical or public health consequences of failing to find a true association? Is it feasible (given funding,

> other resources, or practical constraints) to conduct a study with very high power?

Expert 8

> 80% is conventional and I would also say that is in general the minimal acceptable level. Higher would of course be better but generally comes at a cost (sample size, resources, participant burden, etc etc)

Expert 5

> I think 80% is the minimum power accepted generally by epidemiologists. However, problematically, they rarely include provisions for the effects of confounding and measurement error in their sample size calculations during design or power calculations post hoc, so many studies in occupational and environmental epidemiology are likely underpowered (i.e. less than 80%).
>
> For long term, costly studies, I believe the power should be higher than 80% as the costs need to be justified and a 20% chance of being "wrong" is too high, especially for exposures that may affect large segments of the population.

Expert 4

> 90%, which means we will fail to detect statistical significance in 1/10 of the analysis.

Result 4.8 *(ID: 6591)*
Question 4.8 *(ID: 5976)*

Do you think that type 1 error (p-value) of 0.05 (two-sided) is a suitable default in epidemiologic research involving biomarkers of environmental exposure? Please note that even within epidemiology some specialties rely on p=0.1 but in other disciplines far smaller p-values deemed “significant”. If you choose "No", please specify the p-value you would propose as an alternative value in the answer explanation box.

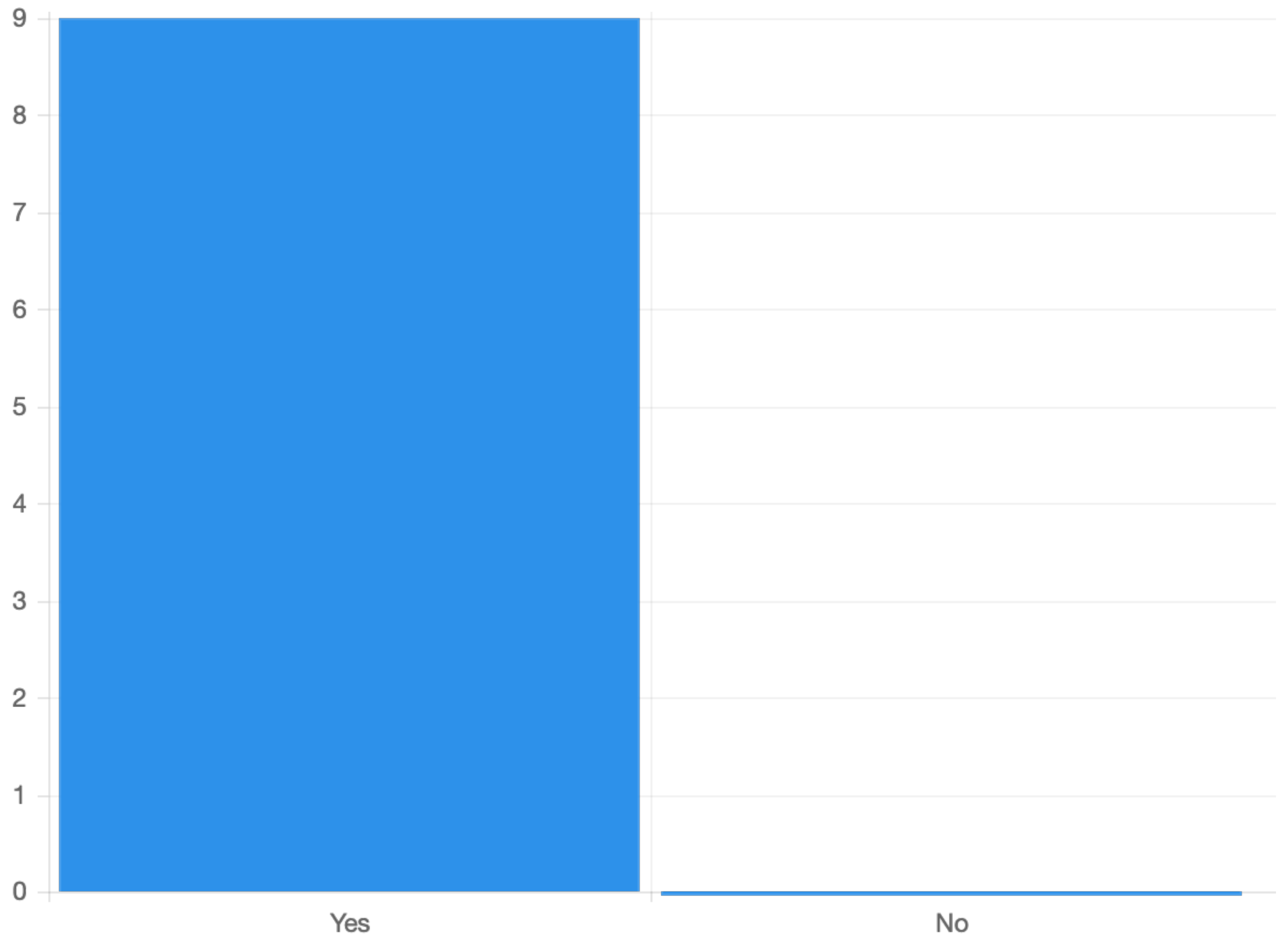

Legend

answers: 9
skips: 0

Answer Explanations

Expert 3 Explanation
Selected Answer(s): Yes

I think that a p-value of 0.05 is as useful as any other value. The real question is: for what fraction of the population of interest do you want to be certain, and what effect size are you considering.

Expert 2 Explanation
Selected Answer(s): Yes

The use of a type 1 error (p-value) of 0.05 (two-sided) as a default in epidemiologic research involving biomarkers of environmental exposure is generally suitable, but it may not be optimal for all situations. The 0.05 threshold is widely accepted because it balances the risks of false positives (type 1 error) and false negatives (type 2 error) in most research contexts. However, it is a somewhat arbitrary standard and might not always reflect the specific requirements of studies involving biomarkers of environmental exposure, where results can have significant public health implications.

In fields where biomarkers are subject to high variability or where public health decisions are made based on findings, a lower p-value threshold (e.g., 0.01) could be more appropriate to reduce the likelihood of false positives and ensure that associations are robust and reliable. Conversely, in exploratory studies or those with limited sample sizes, where detecting weak signals is more challenging, a p-value threshold of 0.1 might be justifiable to avoid missing potential associations.

Personally, while 0.05 is generally accepted, I believe a more context-specific approach is often warranted, with stricter thresholds in confirmatory studies and potentially higher thresholds in exploratory settings.

Expert 7 Explanation

Selected Answer(s): Yes

It is a suitable default. But one should not use ANY number to compare to - significant/non-significant is not a useful dicotomy - one should look at how narrow the confidence interval is and how precisely a parameter of interest is estimated.

Expert 1 Explanation

Selected Answer(s): Yes

I have recently come across this exact problem. A colleague was conducting logistic regression analysis using the p-value cut-off of 0.1. When I reviewed their models, it was clear that too many factors were being included in the analysis. Given that biomonitoring studies have greater limitations this approach would not be acceptable, it would only muddy the waters. Using $p<0.05$ provides a more strict cut-off and allows the inclusion of more definitive variables.

Expert 9 Explanation

Selected Answer(s): Yes

This seems like a suitable default. But "default" suggests that there may be other situations where alternatives are appropriate. For example, exploring novel hypotheses might encourage a looser p value of 0.10, so that promising leads are not excluded. But confirmatory or high-stakes studies might want to use a more stringent p value such as 0.01 or lower. That can also be true if there are many associations being tested (multiple comparisons). Personally and where possible, I prefer using confidence intervals over p values; I think they convey more information.

Expert 8 Explanation

Selected Answer(s): Yes

I would say it is a suitable default (adjusted for multiple testing where needed), but of course, one should look beyond the exact p-value when interpreting results! It's never black or white. Therefore it's crucial to report estimates and measures of variation around effect estimates - these should guide interpretation (together with knowledge of biases in the study, confounding, analyses, etc.), not the p-value.

And of course, a p-value alone should also not guide important policy, regulation, or other decisions beyond the

interpretation of study results in a scientific paper.

Expert 5 Explanation

Selected Answer(s): Yes

I'm not a fan of these hard cut-points for p-values, but generally agree with this.

Expert 4 Explanation

Selected Answer(s): Yes

It is an arbitrary value and I don't think there exists a "best" p-value.

Result 4.9 *(ID: 6592)*
Question 4.9 *(ID: 5977)*

Assuming that there is sufficient statistical power by design after accounting for biomarker variability, how much percent bias due to measurement error do you think is tolerable before adjusting for such bias using a suitable statistical method? As a point of reference, some believed that bias from confounding on the order of less than 10% is tolerable, hence the popular heuristic of adjusting for covariates if they alter point estimate of the effect estimate of interest by more than 10%. Please specify and explain your choice.

Expert 3

> I think that 10% is as useful as any other value. I do believe it would be helpful to correct for any bias present in the study, but I am also curious about how the bias correction would be implemented.

Expert 2

> In my view, when designing epidemiologic studies involving biomarkers of environmental exposure, I consider a bias due to measurement error of around **10% or less** to be tolerable before applying a statistical adjustment. This aligns with the common heuristic used for confounding, where a bias exceeding 10% is typically viewed as a threshold for adjustment.
> The rationale behind this threshold is that smaller biases (under 10%) are unlikely to substantially distort effect estimates or lead to misleading conclusions, especially in studies with sufficient statistical power. However, when bias from measurement error exceeds 10%, it can significantly impact the accuracy of the association between exposure and outcomes, potentially masking true effects or introducing false ones. Given the inherent variability in biomarker levels, even moderate biases can weaken the validity of results if left unadjusted.
> While the 10% threshold is somewhat arbitrary, I find it provides a practical balance—ensuring that we address meaningful bias without over-correcting. In cases where biomarker variability is particularly high, or the study's outcomes have critical public health implications, I might argue for an even lower threshold to maintain confidence in the findings.

Expert 6

> 10% is fine, if supplemented by a sensitivity analysis that considers confounding and within-person variability by age, metabolism, and timing of sample, irrespective of imprecision of exposure measurement on single samples.

Expert 7

> I do not think "cookbook" approach is appropriate. Whether to adjust or not depends on the how much data is available, how many variable you need to adjust, and a change in the estimate due to adjustment. Often it is important to present both unadjusted and adjusted values.

Expert 1

> This is the trick question! How long is a piece of string? Bias should always be minimized as much as possible. Similar to bias assessment used in systematic reviews/meta-analysis, a spectrum approach might be reasonable (i.e., 0-3% Excellent; 4-6% Good; 7-10% fair; >10% not acceptable).

Expert 9

> I think 10% is acceptable. I prefer, where feasible and not overwhelming to the reader, to present results both unadjusted for covariates and adjusted. That way the reader can get a sense of the impact of adjustment for themselves. Again, I like the idea of doing sensitivity analyses using upper and lower bounds of important covariates, for the same reason.

Expert 8

> First, i think the rule of thumb for a 10% change in estimates for choosing covariates is not appropriate. I understand the wish for such a pragmatic rule, but e.g. not including many confounders which all have ~9% effect on the estimates would results in significantly biased estimates. i think choice for including covariates as potential confounders should always be driven by prior knowledge, theory, knowledge from previous studies or biological pathways, etc. DAGs are useful to visualize this and make decisions.
> I guess the same holds for the size of bias in exposure assessment. One would want to reduce it as much as possible and then take what we know about the potential measurement errors into account in interpreting results, whether it's estimated at 1 or 20%. Knowledge of the direction of the bias, whether the error is differential or not, etc. is also crucial. Adding a threshold is too simplistic and could lead to less critical interpretations.

Expert 5

> I think that this depends somewhat on the scale of measurement, the outcome measurement and the risk estimate used (OR vs RR). I think that 10% is reasonable, but these adjustments need to be evaluated with the data at hand (i.e. are estimates changes and precision increased?).

Expert 4

> At most 5%, if larger than 5% it would be about 50% of the effect of a covariate (that affect the effect estimate by 10%).

Result 4.10 *(ID: 6593)*
Question 4.10 *(ID: 5978)*

How important is it to calculate the impact of measurement error in covariates (potential confounders) in estimating power and bias of epidemiologic studies during planning stages? (please explain)

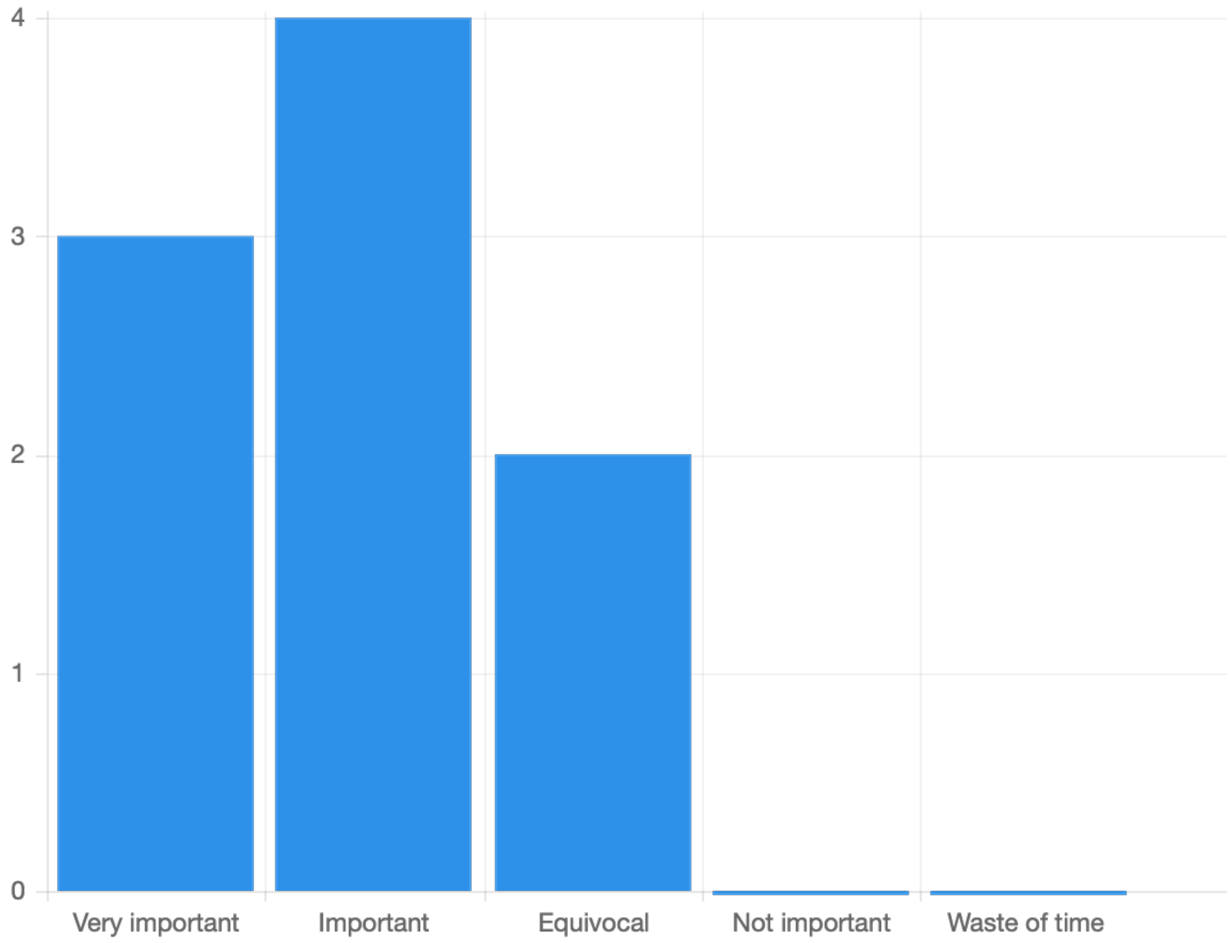

Legend

answers: 9
skips: 0

Answer Explanations

Expert 3 Explanation
Selected Answer(s): Very important

It depends on the effect size of the confounders. For example, confounders affect effect estimates. Without calculating the impact of measurement error in covariates, one cannot be certain of its influence. Therefore, it is necessary to calculate the measurement error in the covariates.

Expert 2 Explanation
Selected Answer(s): Very important

In my experience, it's crucial to calculate the impact of measurement error in covariates, especially potential confounders, when estimating power and bias during the planning stages of an epidemiologic study. Measurement

error in covariates can significantly bias the results by introducing residual confounding. Even small inaccuracies in key confounders can lead to incorrect estimates of the relationship between exposure and outcome. This kind of bias can persist even if exposure measurements are accurate, which is why I always emphasize the need to account for potential errors in covariates.
Measurement error in covariates also affects the study's statistical power. If covariates are misclassified or imprecise, the variability in the data increases, reducing our ability to detect true associations between exposure and outcome. Even with a well-powered study design, covariate measurement error can significantly reduce power if not addressed properly.
By evaluating the potential impact of covariate measurement error early in the planning stage, we can make better decisions about study design. For instance, as a biostatistician, I may recommend increasing the sample size or improving the accuracy of certain covariate measurements. This way, my colleagues can balance resource use with the need for precise and reliable data. Overall, I believe that calculating the effect of covariate measurement error is essential for ensuring the robustness of the study design, and it allows us to provide more accurate estimates of both power and bias upfront.
However, while it is ideal to calculate the impact of measurement error, practically speaking, this can be challenging to achieve. These calculations often require advanced statistical techniques, such as simulation studies or measurement error models, which may not be straightforward to implement. Additionally, these models rely on a number of assumptions about the nature of the error, the structure of the data, and the distribution of covariates, which may not always hold in real-world studies. As a result, the ability to accurately estimate the impact of covariate measurement error can be limited. Therefore, while I always advocate for its consideration, I also recognize that there may be practical limitations, and researchers must carefully assess the feasibility of incorporating such adjustments into their study design.

Expert 7 Explanation
Selected Answer(s): Equivocal

As discussed above, power rarely drives design decisions. I am much more interested in bias though, because it can drive the design.

Expert 1 Explanation
Selected Answer(s): Important

I think it is important to be able to provide estimates for the impact of confounding factors on measurement error. The ability to assess this impact clearly improves the quality of the study and the findings.

Expert 9 Explanation
Selected Answer(s): Important

This can be helpful if the appropriate data are available. If there is considerable measurement error, it may weaken the impact of adjusting for covariates and thus weaken the overall study, especially if it allows considerable bias in estimates to occur.

Expert 8 Explanation
Selected Answer(s): Important

Important, as adjusting for poorly measured confounders will result in residual confounding, but i.m.o. somewhat less than measurement errors in exposure assessment.

Expert 5 Explanation
Selected Answer(s): Important

Generally we don't have a good understanding of these relationships (i.e. correlations between confounders and exposures/outcomes, and their proxy measurements) in order to perform quantitative bias calculations. If we have the information, we should attempt at a minimum sensitivity analyses.

Expert 4 Explanation

Selected Answer(s): Very important

The measurement error will greatly affect the sample size required, and sometimes may even be unable to obtain an unbiased effect, so these impacts should be known to the investigators before conducting the study.

Result 4.11 *(ID: 6594)*
Question 4.11 *(ID: 5979)*

How important is it to calculate the impact of measurement error in effect modifiers in estimating power and bias of epidemiologic studies during planning stages?

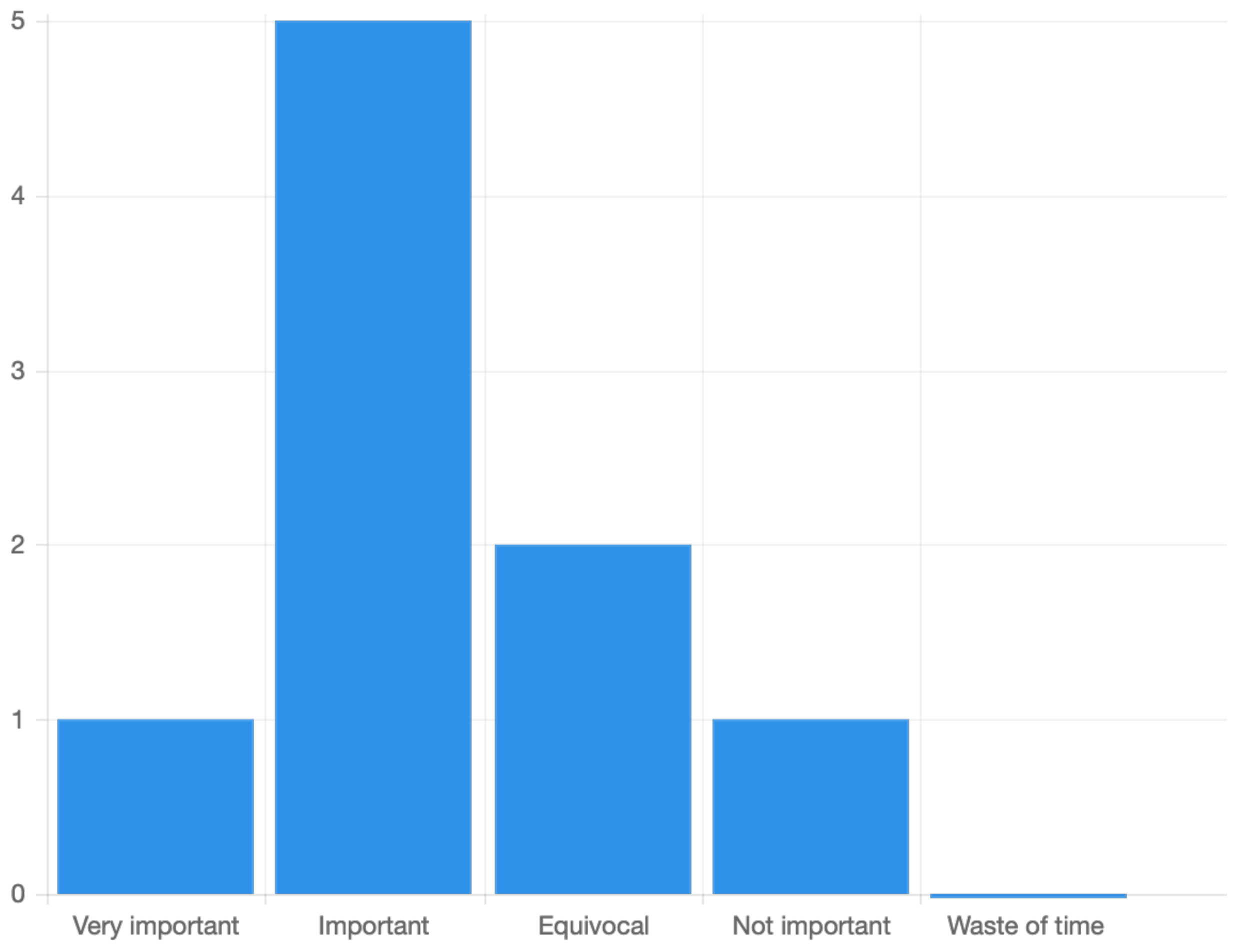

Legend

answers: 9
skips: 0

Answer Explanations

Expert 3 Explanation
Selected Answer(s): Very important

It depends on the size of the measurement error. For example, measurement errors of effect modifiers affect effect estimates. Without calculating the impact of measurement error, one cannot be certain of its influence. Therefore, it is necessary to calculate the measurement error in effect modifiers.

Expert 2 Explanation
Selected Answer(s): Important

In my opinion, it's also important to account for measurement error in effect modifiers during the planning stages of epidemiologic studies. Effect modifiers can influence the relationship between exposure and outcome, and errors in their measurement can obscure interactions, leading to biased estimates and incorrect conclusions

about subgroup effects. Additionally, this type of error can reduce the power to detect true interactions. Addressing this upfront helps design a study that better estimates interaction effects and minimizes the risk of false positives or negatives.
However, when comparing measurement error in confounders and effect modifiers, I believe measurement error in confounders is more critical. Confounders, if mismeasured, can directly bias the overall exposure-outcome estimate, potentially leading to spurious or masked associations. Even small errors in confounders can significantly distort results, whereas effect modifiers primarily affect subgroup or interaction analysis without directly biasing the main exposure-outcome relationship.
Overall, while both are important, I prioritize addressing measurement error in confounders, as it has a more direct and widespread impact on study validity. Measurement error in effect modifiers, while important for subgroup analysis, has a more nuanced effect on overall conclusions.

Expert 6 Explanation
Selected Answer(s): Equivocal

Consideration of effect modifiers (such as age, sex, or race) is more important, especially since these are usually measured with little or no error. This is especially important if there are strong reasons to suspect effect modification by such factors.

Expert 7 Explanation
Selected Answer(s): Not important

Effect modifiers are mostly unknown during planning, even more unknown would be a measurement error of effect modifiers.

Expert 1 Explanation
Selected Answer(s): Important

This again would be important, to be honest, I don't think I've ever seen it done in a biomonitoring study.

Expert 9 Explanation
Selected Answer(s): Equivocal

It seems that assessing the impact of measurement error of effect modifiers could be helpful, but is probably not as important as measurement error of confounders. Also, many of the effect modifiers commonly used in public health (e.g., age, sex, and to some extent race) are measured with little error. However, some other effect modifiers that might be used (e.g. household income, urban/rural status) might have considerable error.

Expert 8 Explanation
Selected Answer(s): Important

Depends on how important the effect modifier is + other factors in the study design. If e.g. differences by different subgroups are very large one will likely pick it up, also if there is some measurement error in the estimation of the effect modifier. If the modification is modest or the study has low power, one could miss potential effect modifiers, not pick them up in interaction analyses.
Also, when they are used for stratification, measurement error can lead to misclassification across strata.
In general, interaction analyses and stratified analyses require larger sample sizes.
Overall: somewhat important but less than measurement error in exposures and often probably also a bit less important than measurement error in confounders and outcomes.

Expert 5 Explanation

Selected Answer(s): Important

For effect modifiers, my answer to this question is similar to my answer re: confounders above. Theoretically, I believe that error in effect modifiers may result in bias in more significant ways as analyses are often stratified based on a level of an effect modifier. If one level has low numbers, this can result in a significant lack of power for estimating the interaction effects. Generally, the greatest power is achieved when the prevalence of dichotomous effect modifier is 50%.

Expert 4 Explanation

Selected Answer(s): Important

As above, very important to have a sense of the impact, but sometimes it is difficult to quantify the impact due to lack of knowledge on the exact measurement error in the study population.

Result 4.12 *(ID: 6595)*
Question 4.12 *(ID: 5980)*

How important is it to calculate the impact of measurement error in the outcome measure in estimating power and bias of epidemiologic studies during planning stages? (please explain)

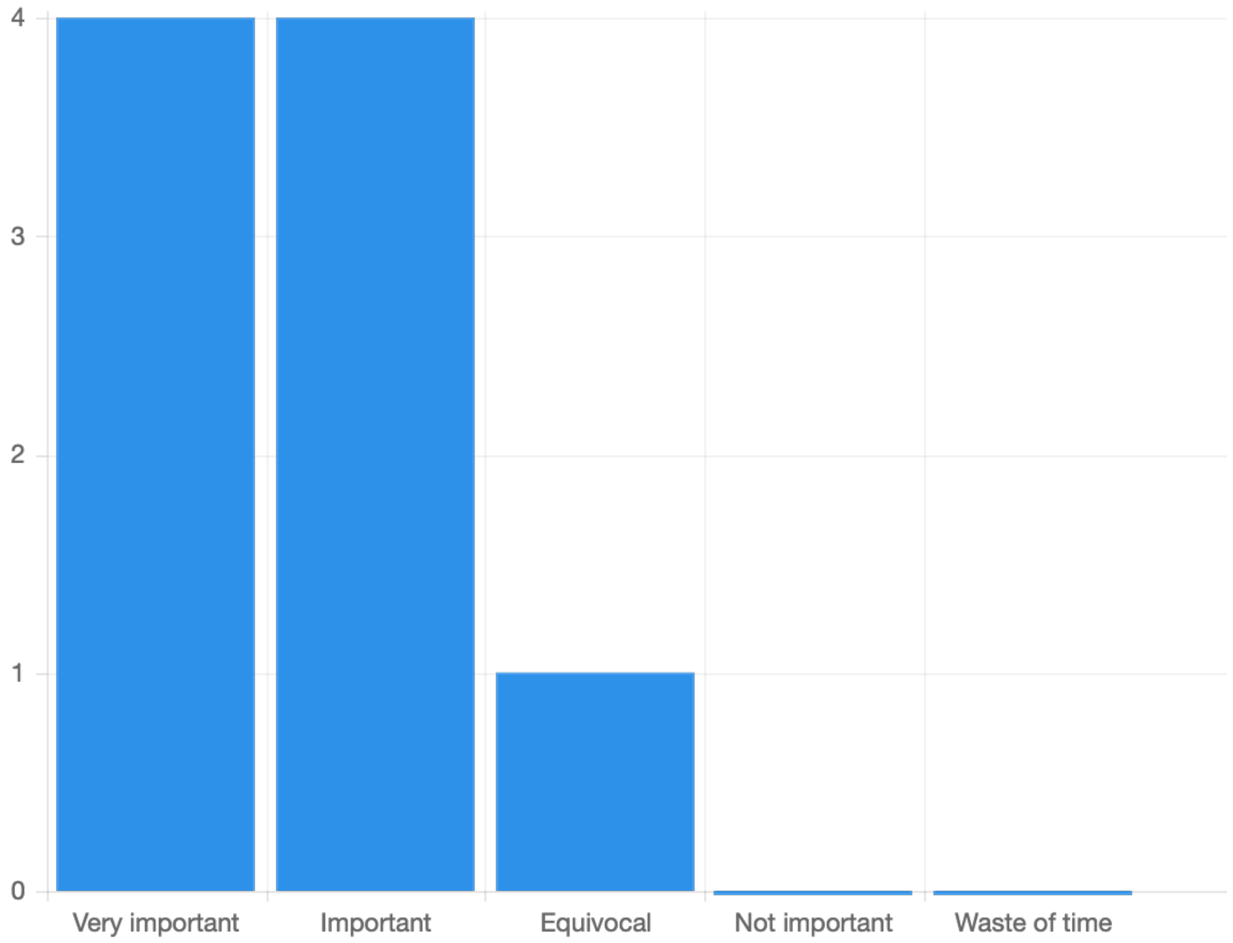

Legend

answers: 9
skips: 0

Answer Explanations

Expert 3 Explanation
Selected Answer(s): Very important

It depends on the effect size of the measurement error in the outcome measure. Without calculating the impact of measurement error, one cannot be certain of its influence. Therefore, it is necessary to calculate the measurement error in the outcomes.

Expert 2 Explanation
Selected Answer(s): Very important

I believe it's very important to calculate the impact of measurement error in the outcome measure during the planning stages of an epidemiologic study. Ignoring measurement error in the outcome can lead to biased effect

estimates. When outcome measurement error is underestimated, it can reduce variability and exaggerate the association between exposure and outcome. This increases the risk of false positives and can significantly undermine the validity of the study's conclusions, particularly when the sample size is small.
By accounting for this error during the planning phase, we can make necessary adjustments to the study design—such as increasing the sample size or improving the accuracy of the outcome measure—to ensure the results are both reliable and unbiased. For this reason, I recommend placing emphasis on the impact of measurement error in the outcome measure when planning a study.

Expert 7 Explanation
Selected Answer(s): Equivocal

Measurement error in the outcome should be minimized at the outset.

Expert 1 Explanation
Selected Answer(s): Important

The measurement error should be minimized so as to provide greater validity to the outcome.

Expert 9 Explanation
Selected Answer(s): Very important

Measurement error in the outcome must be considered when designing a study. First, the researcher should try to take all feasible steps to minimize measurement error in the outcome through study design, data collection techniques, etc. But some measurement error will inevitably remain, and this should be taken into account in estimating power and bias.

Expert 8 Explanation
Selected Answer(s): Important

Important but i.m.o somewhat less than errors in exposure assessment

---

Expert 5 Explanation
Selected Answer(s): Important

The inclusion/exclusion criteria should be clear and properly classify individuals into groups. If a continuous outcome is measured, it is important to understand the relationship between the measured outcome and the true outcome. Sample sizes can be increased and/or repeated measurements within subjects over time can improve study power. Also, statistical adjustments and/or sensitivity analyses can be performed.

Expert 4 Explanation
Selected Answer(s): Very important

I believe any researchers will minimise the measurement error in the outcome measure, but still if error exists it should be estimated.

Result 4.13 *(ID: 6596)*
Question 4.13 *(ID: 5981)*

Biomarkers of exposure typically follow a skewed frequency distribution typified by a lognormal one. It is common to log-transform such measurements in analysis and to plan studies (as we do in our calculators) using normally distributed exposures that are derived from log-transformation of the raw biomarker data. An alternative approach is to use untransformed biomarkers in their original units. Which approach do you think is the best practice in epidemiology studies that employ biomarkers to estimate for each participant their own exposure (individual-based exposure assessment, as is the focus of our calculators)? (please explain)

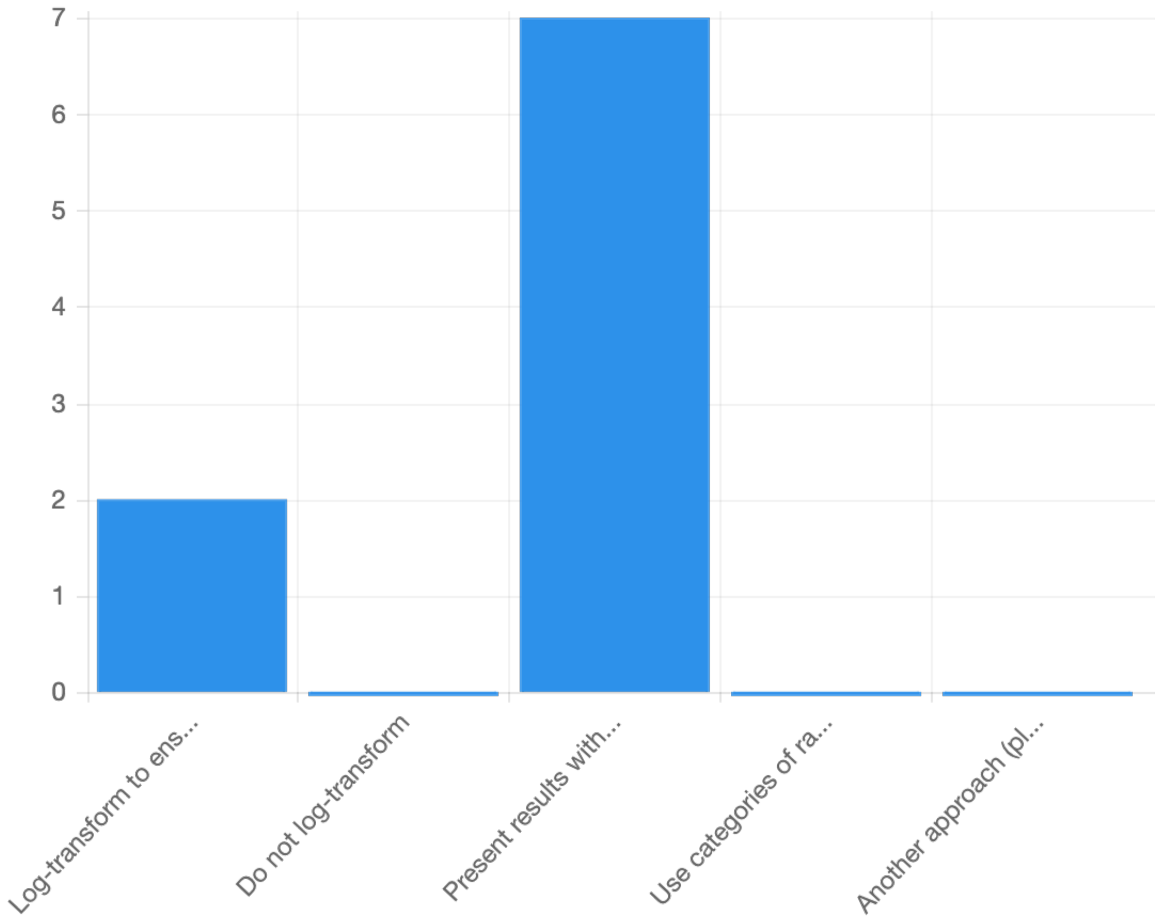

Legend

Log-transform to ensure normality: 2
Do not log-transform: 0
Present results with and without log-transformation of biomarkers: 7
Use categories of ranks of exposure that are the same regardless of transformation: 0
Another approach (please explain): 0

answers: 9
skips: 0

Answer Explanations

Expert 3 Explanation

Selected Answer(s): Log-transform to ensure normality

Environmental exposures are typically log-normally distributed, i.e., high exposures are rare, and low exposures are common. Hence, it is expected that biomarkers will also follow a log-normal distribution. However, log-transforming data is not always a choice—it depends on the type of analysis being used. For example, a simple OLS regression analysis assumes that the data is normally distributed. If the data is log-normally distributed, the results of the regression analysis will be incorrect unless the data is log-transformed. In contrast, logistic regression does not assume normally distributed data, so log transformation is not necessary.

Expert 2 Explanation

Selected Answer(s): Log-transform to ensure normality

In epidemiology studies that use biomarkers for individual-based exposure assessment, log-transforming biomarker data is generally the recommended practice, especially when the data follow a skewed distribution, as is common with exposure biomarkers. Log transformation normalizes the distribution, making it more symmetrical and suitable for parametric statistical models that assume normality. It also stabilizes variance across different exposure levels, improving the precision and consistency of effect estimates. Additionally, log-transformed data can be seen as a more precise version of ranks, preserving the order of data while providing detailed information about the magnitude of differences between values. This makes the interpretation of associations more meaningful in public health contexts. Furthermore, log-transformed data enhances model performance, as statistical methods like linear regression perform better with normally distributed data. While using original units or ranks with non-parametric methods may be considered, it can result in skewed residuals and reduced statistical power. Therefore, I prefer log transformation for yielding more accurate, interpretable, and statistically robust results, though the interpretation of the results should always take into account the original data distribution and the study context.

Expert 7 Explanation

Selected Answer(s): Present results with and without log-transformation of biomarkers

Examine frequency distribution of a biomarker and then decide on approach.

Expert 1 Explanation

Selected Answer(s): Present results with and without log-transformation of biomarkers

I think providing different options is acceptable and provides scientists with alternatives. Some may wish to use log-transformations, others may not, if both are available that would be optimal.

Expert 9 Explanation

Selected Answer(s): Present results with and without log-transformation of biomarkers

I would prefer to first examine the frequency distribution of the biomarker and then decide what to do based on that. If it exhibits a roughly log-normal distribution, then proceed with that transformation. But depending on your study population, it might be something else. For example, it might be sufficiently normal and not require transformation at all. It might be something quite different, like bimodal, For odd distributions, I would consider non-parametrics, ranking/categorizing, or other approaches.

If exposure data are transformed, I like to be transparent and present results with and without the transformation so that the reader can form their own interpretations and appreciate the impact of the transformation.

Expert 8 Explanation

Selected Answer(s): Present results with and without log-transformation of biomarkers

In general, I'm very much in favor of reporting multiple types of estimates, e.g. by different transformations vs not, different units/categories, different levels of adjustment, other analytical approaches, etc. Although this can make tables large (can be presented in supplemental files) and interpretation sometimes challenging, it facilitates comparison between studies and meta-analyses of findings.

More specifically: log-transformation may result in a more normal distribution and therefore meeting assumptions of many statistical tests. I would present these as main results. But adding the untransformed values and estimates will help in interpreting effect sizes by different audiences and help guide e.g policy change. I would see analyses of untransformed values also as a sort of sensitivity analyses, if results are greatly different, one might want to check the underlying data, models and assumptions again (e.g. maybe logtransformation obscured non-linear associations).

Expert 5 Explanation

Selected Answer(s): **Present results with and without log-transformation of biomarkers**

I would not limit myself to log-transformations only. We would typically evaluate different transformations (i.e. sq root, x2 etc.) to normalize the residuals in the analysis (note, we are generally looking for multivariate normal distribution). In addition, we almost always analyze the ranked data (non-parametric) and/or ordinal categories and compare these results. Depending on the analyses, we may only publish some of the results depending on the Journal. I like to include these other analyses in appendices if possible, so that readers/reviewers can evaluate the assumptions/decisions made.

Expert 4 Explanation

Selected Answer(s): **Present results with and without log-transformation of biomarkers**

I don't think there exists a single best solution so I suggest to present both results.
