# Supplementary material for: Improving the design of epidemiology studies that use biomonitoring for exposure assessment: a SciPinion panel recommendation
Source: BMC Med Res Methodol. 2026 Jan 12;26:29. doi: 10.1186/s12874-025-02753-5 (PMC12888676; doi:10.1186/s12874-025-02753-5)
Supplement: Supplementary file 4 — Additional file 4. The white paper that served as the review material for the SciPinion panel of experts. [file 12874_2025_2753_MOESM4_ESM.pdf]

Improving Epidemiology Study Designs for Use of Biomonitoring of Exposure in the Presence of Classical Measurement Error: Calculators of Power and Bias

Authors (to be disclosed after completion of the SciPi)

Abstract

This paper presents a suite of statistical calculators designed to calculate sample sizes (number of individuals sampled, *n*, and number of samples per individual, *m*) and illustrate how power and bias vary with *m* and *n* in epidemiological studies that use biomonitoring for exposure assessment in the presence of classical (additive, normally distributed) measurement errors. Approaches that ignore variability in exposures within and between individuals can bias effect estimates and lead to inaccurate risk assessment. Our tools help to address this gap by estimating the required sample sizes, number of repeated measurements, and the trade-offs between power and bias in linear and logistic regression models under classical measurement error assumptions.

We explain within- and between-individual variability and the intraclass correlation coefficient (ICC) and then introduce calculators for determining the number of repeats per individual needed to achieve a desired validity coefficient, number of individuals to sample for a specified margin of error and number of repeats, and the trade-off between these sample size decisions (*m* and *n*) and the minimum detectable effect (MDE) for linear and logistic regression models.

The practical application of these calculators is demonstrated through motivating examples and real data sets, such as air quality monitoring and NHANES biomarker data. Results from the calculators provide insights into the impact of measurement error on study design, emphasizing the need for adequate number of individuals and number of repeated measurements to achieve robust findings.

Our findings underscore the importance of integrating these calculators into epidemiological research to support better-informed public health decisions and interventions. By providing user-friendly tools that account for measurement error, researchers can design more accurate and reliable studies, ultimately improving public health outcomes.

Table of Contents

1 INTRODUCTION 4
1.1 BACKGROUND: WITHIN- AND BETWEEN-INDIVIDUAL VARIABILITY IN EXPOSURE 4
1.2 PROBLEM STATEMENT 8
1.3 RISK ASSESSMENT AND MANAGEMENT CHALLENGES 9
1.4 DATA-INFORMED DECISIONS 9
1.5 PURPOSE OF THIS PAPER 10
2 MOTIVATING EXAMPLES AND DATA 10
2.1 EXAMPLE 1: ASSESSING THE IMPACT OF AIR POLLUTION ON RESPIRATORY HEALTH 10
2.2 EXAMPLE 2: BIOMARKER VARIABILITY IN NUTRITIONAL STUDIES 11
2.3 EXAMPLE 3: LONGITUDINAL STUDIES ON CHRONIC DISEASE PROGRESSION 12
2.4 EXAMPLE 4: ENVIRONMENTAL EXPOSURE AND REPRODUCTIVE HEALTH 12
2.5 DATA EXAMPLE 1: EPA AIR QUALITY MONITORING DATA 12
2.6 DATA EXAMPLE 2: NHANES BIOMARKER DATA 13
2.7 DATA EXAMPLE 3: LONGITUDINAL COHORT DATA FROM THE FRAMINGHAM HEART STUDY 13
2.8 DATA EXAMPLE 4: BIRTH COHORT STUDIES ON ENVIRONMENTAL EXPOSURES 13
3 METHODS 14
3.1 THE "NUMBER OF REPEATS" CALCULATOR" QUANTIFIES REPEATS REQUIRED TO ACHIEVE A DESIRED VALIDITY COEFFICIENT (HTTPS://SCIPINION.SHINYAPPS.IO/REPEATSFORDVC/) 14
3.2 SAMPLE SIZE CALCULATOR FOR MEAN WITH DESIRED MARGIN OF ERROR (HTTPS://SCIPINION.SHINYAPPS.IO/SAMPLESIZEMOE/) 15
3.3 SAMPLE SIZE AND MINIMUM DETECTABLE EFFECT (MDE) TRADEOFF CALCULATOR FOR LINEAR REGRESSION (HTTPS://SCIPINION.SHINYAPPS.IO/LINEARREGRESSIONN/ AND HTTPS://SCIPINION.SHINYAPPS.IO/LINEARREGRESSIONM/) 16
3.4 SENSITIVITY ANALYSIS EXPLORER FOR LOGISTIC REGRESSION (HTTPS://SCIPINION.SHINYAPPS.IO/SENSITIVITYANALYSISEXPLORER/) 17
4 RESULTS 18
4.1 NUMBER OF REPEATS NEEDED FOR DESIRED VALIDITY COEFFICIENT 18
4.2 SAMPLE SIZE CALCULATOR FOR DESIRED MARGIN OF ERROR IN ESTIMATING EXPOSURE LEVEL 18
4.3 SAMPLE SIZE AND MDE TRADEOFF CALCULATION FOR LINEAR REGRESSION 19
4.4 SAMPLE SIZE AND MDE TRADEOFF CALCULATION FOR LOGISTIC REGRESSION 19
5 DISCUSSION 22
5.1 IMPLICATIONS FOR STUDY DESIGN 23
5.2 PRACTICAL CONSIDERATIONS 24
6 CONCLUSIONS 25
7 REFERENCES 27
8 APPENDIX: INSTRUCTIONS AND SOURCE CODE 30
8.1 USER'S GUIDE FOR CALCULATOR FOR MEAN WITH DESIRED MARGIN OF ERROR 30

8.1.1 Introduction ... 30
8.1.1.1 Terminology and Formula ... 30
8.1.2 Accessing the Application ... 31
8.1.3 Using the Application ... 31
8.1.3.1 Interface Overview ... 31
8.1.3.2 Input Controls ... 31
8.1.3.3 Output Display ... 31
8.1.4 Steps to Use the Calculator for Mean with Desired Margin of Error ... 31
8.1.5 Example Calculation ... 31
8.1.5.1 Interpretation ... 32
8.1.5.2 Practical Implications ... 32
8.2 USER'S GUIDE FOR ONE-SAMPLE CALCULATOR USING FLEISS FORMULA ... 35
8.2.1 Introduction ... 35
8.2.2 Accessing the Application ... 35
8.2.3 Using the Application ... 35
8.2.3.1 Interface Overview ... 35
8.2.3.2 Input Controls ... 35
8.2.3.3 Output Display ... 35
8.2.4 Steps to Use the One-Sample Calculator Application ... 35
8.2.5 Example ... 36
8.3 USER'S GUIDE FOR SIMPLE LINEAR REGRESSION (SLR) N (NUMBER OF SUBJECTS) SAMPLE SIZE CALCULATOR WITH MEASUREMENT ERROR ... 39
8.3.1 Introduction ... 39
8.3.2 Overview ... 39
8.3.2.1 User Interface ... 39
8.3.2.2 Input Panel ... 39
8.3.2.3 Output Panel ... 40
8.3.2.4 How to Use ... 40
8.3.2.5 Calculation Details ... 40
8.3.2.6 Derivations and Justifications ... 40
8.4 USER'S GUIDE FOR SIMPLE LINEAR REGRESSION (SLR) M (NUMBER OF MEASUREMENTS PER SUBJECT) CALCULATOR WITH MEASUREMENT ERROR ... 46
8.4.1 Introduction ... 46
8.4.2 Using the Application ... 46
8.4.2.1 Interface Overview ... 46
8.4.2.2 Input Controls ... 46
8.4.2.3 Output Display ... 46
8.4.3 Steps to Use the SLR Sample Size Calculator Application ... 47
8.4.4 Example ... 47
8.5 USER'S GUIDE FOR LOGISTIC REGRESSION SENSITIVITY ANALYSIS EXPLORER ... 51
8.5.1 Introduction ... 51
8.5.2 Running the Sensitivity Analysis Explorer Application ... 51
8.5.3 Using the Application ... 51
8.5.3.1 Interface Overview ... 51
8.5.3.2 Input Controls ... 51
8.5.3.3 Output Display ... 52
8.5.3.4 Steps to Use the Sensitivity Analysis Explorer Application ... 52
8.5.3.5 Example ... 52

1 Introduction

Developing and applying calculators to determine recommended sample sizes when individual exposures are measured or estimated with error are crucial in epidemiological research, including studies that use biomarkers of exposure. Traditional approaches often do not account for the inherent variability of exposures both within and between individuals. This can bias effects estimates in epidemiologic research and lead to inaccuracies in risk assessment and management. The need for more precise and reliable data to inform decisions in public health and clinical settings drives the creation of these specialized tools to estimate power and bias in two exposure-response models commonly fitted by epidemiologists (linear and logistic) under classical measurement error in exposure – that is, when exposures are measured with independent, normally distributed errors.

1.1 Background: Within- and Between-Individual Variability in Exposure

The use of biomonitoring (detection of chemicals in blood, urine, saliva, nails, hair, etc.) to estimate exposure in epidemiology has exploded in recent decades as new analytical techniques have become available to measure increasingly small concentrations of analytes in biological media (Paustenbach and Galbraith, 2006). We assume here that sampling and analytical techniques have negligible errors, orders of magnitude smaller than those related to between- and within-person variability in exposure and exposure biomarkers. Biomonitoring has important uses in the study of human exposures to chemicals in our environment via air, water, food, and dermal absorption. It can include a range of data collection schemes, from collecting single samples to pooling samples, either within or among individuals. Biomarkers of exposure are often seen as advantageous over measures of external exposure because they are less variable, fluctuating at random around true value to a lesser degree due to "physiological dampening" (Rappaport & Spear RC, 1988) and because biomonitoring of the chemical integrates all sources and routes of exposure (Sexton et al., 2004).

When biomonitoring first started being used in epidemiology studies, the analytes were compounds with relatively long half-lives ranging from months (lead and other

heavy metals) to years (dioxins, PCBs, etc.). More recently, environmental epidemiology studies have started to use biomonitoring to quantify exposures to short-lived compounds with half-lives on the order of minutes to hours or a few days (LaKind et al. 2019). Many of these short-lived compounds have substantial intra-individual variability, especially in urine (Preau et al. 2010, Smolders et al. 2019, Koch et al. 2019). For some compounds, including phthalates and bisphenol-A (BPA), the concentration of parent compound and/or metabolites can vary by several thousand-fold within single individual in a single day. Thus, for some compounds, taking a single urine sample from an individual is likely to yield highly uncertain estimates of the person's typical (average) exposures over the time intervals of interest for most types of effects being studied, e.g., cancer (lifetime) and birth outcomes (months).

Preau et al. (2010) collected every urine void for seven days on eight individuals, and estimated the magnitude of variability in the concentration of certain analytes in urine (Figure 1). Follow-up studies (Koch et al., 2019; Smolders et al., 2019) have confirmed the findings of Preau et al. (2010) and provided data on some additional compounds. For some compounds, such as BPA, it appears that people are exposed to approximately the same level (small between-person variance, denoted by V_b) but still have high within-person variance (denoted by V_w) for analytes in urine. Other compounds, such as triclosan, exhibit smaller within-person variability but larger between-person variability, indicating more varied exposures across the population.

Figure 1: Fluctuations of biomarkers for monoethyl phthalate (MEP) over the course a week among eight persons. (Source: Reproduced from Preau et al. (2010))

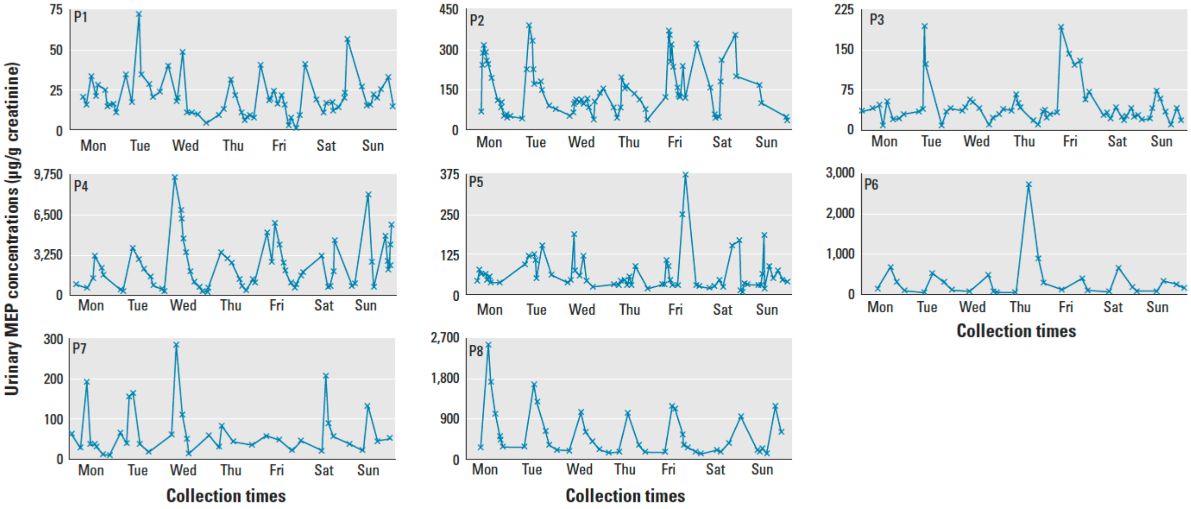

Figure 1. Creatinine-corrected concentrations of MEP (μg/g creatinine) for all study participants (P1–P8) during 1 week.

Between-person variability reflects the differences in exposures between individuals, e.g., because some people use a product containing the chemical (e.g., toothpaste that contains triclosan) while others may not. Within-person variability may reflect time-varying metabolism and pharmacokinetics as well as time-varying exposure. To describe different combinations of within- and between-person variability, it is convenient to compute the *intraclass correlation coefficient* (ICC), defined as the ratio of between-person to total variance (Fleiss, 1986):

$$ICC = \frac{Vb}{(Vb+Vw)} \qquad (1)$$

The statistical estimation of ICC follows fitting of random effects analysis of variance (ANOVA) to the data with repeated measurements of biomarkers on sample of subjects, assuming that both the subjects and times of measurements are chosen at random. ANOVA yields population estimates of V_b and V_w that are then plugged into expression (1).

The value of the ICC ratio can range from 0 (if $V_b = 0$) to 1 (as V_b becomes very large compared to V_w). While the ICC is specific to a chemical, it can also vary as a function of the length of averaging (compositing) time and individual hydration status correction (e.g., use of creatinine adjustment, etc.) (Table 1).

Table 1: Estimated ICCs for analytes included in the CDC (Preau et al., 2010) and CEFIC (Koch et al. 2019) studies across analytes, spot versus 24-hour composites, and hydration status correction. ICC values > 0.8 are shown in red.

	Analyte	Unadjusted, ug/L		Cr-adjusted, ug/g cr	
		Spot samples	24-hr composites	Spot samples	24-hr composites
CDC	MEP	**0.61**	**0.91**	**0.73**	**0.93**
	MEHHP	0.23	0.39	0.21	0.33
	BPA	0.20	0.32	0.12	0.16
CEFIC	Methyl paraben	**0.56**	**0.84**	**0.71**	**0.87**
	Triclosan	**0.93**	**0.98**	**0.96**	**0.99**
	BPA	0.13	0.19	0.26	0.28

In epidemiological studies that employ biomarkers of exposure, it is typical to collect multiple measurements of the biomarkers and average them, with the average value then being plugged into a statistical model that relates exposure estimates to the outcomes. Such approaches have a long history in epidemiologic research and predate use of biomarkers, when measurements of externally measured exposure, which is often even more variable than biomarkers, were averaged to reduce measurement error and associated biases (Preller et al. 1995, Tielemans et al. 1998, Kim et al. 2011). More formally, *random effects models* assume that biomarker measurements are generated by the following process:

$$Z_{ij} = \mu + \alpha_j + \varepsilon_{ij},$$

where Z_{ij} is the ith observation on the jth person, μ is an unobserved overall mean, assumed to be stationary over time, α_j is an unobserved random effect shared by all biomarker measurements of person j, and ε_{ij} is an unobserved error term. For the ith

measurement on person j, the α_j and ε_{ij} are assumed to be normally distributed, have expected value zero, be independent of each other, have identical distributions, and remain stationary. The variance of α_j is denoted V_b and the variance of ε_{ij} is denoted V_w.

When a single sample is collected from each individual, its observed value is used as an exposure estimate in epidemiology (m = 1). If m > 1 samples are collected per individual, then their average for an individual is used as that person's exposure estimate, denoted as $\overline{Z_J}$. The following classical additive measurement error model relates $\overline{Z_J}$ to true unobserved exposure X_j:

$$\overline{Z_J} = X_j + \varepsilon_Z,$$

where X_j and ε_Z are assumed to be independent; exposure is assumed to be normally distributed in the population with a fixed, stationary individual mean $\mu + \alpha_j$, and with a constant inter-individual variance V_b, $X_j \sim N(\mu + \alpha_j, V_b)$; and the error for individual measurements is assumed to be normally distributed with zero mean and constant variance, $\varepsilon_Z \sim N(0, V_e)$, with $V_e = V_w/m$. Note that the correlation (validity coefficient of $\overline{Z_J}$) is $\rho_{xz} = (V_b/(V_b+V_w/m))^{0.5}$, and it follows that $\rho^2_{xz} \neq$ ICC, unless m = 1; following Fleiss (1986) we call ρ^2_{xz} the "reliability of the mean of m independent replicate measurements, or "reliability" for short, and denote it by R_m.

It is known that classical additive measurement error tends to bias slopes of linear regression and odds ratios derived from fitting logistic regression towards the null (no effect) when there is a single independent variable (Gustafson 2004). It is less widely understood that measurement error in exposure degrades statistical power, such that a study designed under the assumption of no measurement error will have higher type II error (false negative) rate than the target one when implemented in presence of measurement error (Armstrong 1996).

1.2 Problem Statement

Accurate measurement and analysis of biomarkers of exposure are pivotal for understanding exposure levels, disease progression, and treatment efficacy. However, the variability in biomarker concentrations within and between individuals poses

significant challenges. This variability can lead to substantial measurement errors in exposure, impacting the validity of study findings and subsequent decision-making processes. The primary problem addressed in this work is the need for calculators that account for such variability, enabling researchers to determine adequate sample sizes and understand the trade-offs between sample sizes (number of individuals sampled, *n*), number of repeats (samples per individual, *m*), measurement error, study power to detect effects of different sizes, and biases in estimated regression coefficients.

1.3 Risk Assessment and Management Challenges

In the context of risk assessment, the variability in biomarkers of exposure can obscure true exposure levels and risk relationships. This can be particularly problematic in studies investigating exposures with “low” ICCs. High ICCs indicate low within-subject variability relative to between-subject variability, while low ICCs suggest the reverse. When ICC = 0 for an exposure, there is no contrast in such an exposure between persons and it is not possible to relate measures of such an exposure to health outcome: everybody has the same exposure, on average. The ICC is a property of the underlying data-generating process and exists independently of how exposures are accessed. This is fortunate, because it creates an opportunity of measure exposures to counter-act bias from low ICC (unless it is equal to zero), by collecting many repeated measurements on a person and thereby reducing the influence of temporal variation in exposure and biomarker levels. Accurately estimating the true exposure levels and exposure-response risk relationships is essential for effective risk management and policy formulation, but accurate estimation requires understanding and adjusting for the ICC. The challenge lies in developing tools that can help researchers and policymakers balance the precision of their estimates with practical considerations such as cost and feasibility.

1.4 Data-Informed Decisions

Effective public health and clinical interventions rely on data-informed decisions. These decisions are only as good as the data and analyses upon which they are based. Inaccurate sample size calculations or misunderstanding the impact of measurement error can lead to either over- or underestimations of risks, rendering interventions less effective or unnecessarily burdensome. The development of calculators for various statistical scenarios, including linear regression and logistic regression models with a single independent variable, exposure, and with classical additive measurement error, provides researchers with the means to make informed choices about study design, ensuring that the collected data will be adequate to meet their objectives.

1.5 Purpose of This Paper

This paper presents a suite of calculators designed to address the challenges of exposure biomonitoring variability and measurement error in epidemiological studies. By providing clear, user-friendly tools, we hope to facilitate more accurate and reliable research, ultimately supporting better public health outcomes through data-informed decisions. Integrating these calculators into epidemiological research during study design allows data analysts and modelers to better account for the inherent variability in biomonitoring data, leading to more precise and actionable insights. This integration enables risk management decisions based on these data to be grounded in robust statistical methodologies, thereby potentially enhancing the overall quality and impact of epidemiological studies.

2 Motivating Examples and Data

2.1 Example 1: Assessing the Impact of Air Pollution on Respiratory Health

One of the most pressing public health concerns is the impact of air pollution on respiratory health. Numerous studies have documented the adverse effects of pollutants such as $PM_{2.5}$ and nitrogen dioxide on conditions like asthma, bronchitis, lung cancer,

adverse birth outcomes, and cardiovascular health. A critical aspect of these studies is the accurate measurement of individual exposure levels, which often exhibit significant variability both within and between individuals. For instance, Schwartz et al. (2016) demonstrated that $PM_{2.5}$ exposure is associated with increased mortality risk, but the variability in exposure levels can introduce substantial measurement error, affecting the robustness of the findings. By employing our calculators, researchers can determine the sample sizes needed to achieve desired confidence intervals for different levels of measurement error, thereby strengthening the validity of their conclusions under the resource constraints that always limit data collection.

2.2 Example 2: Biomarker Variability in Nutritional Studies

In nutritional epidemiology, biomarkers such as blood glucose levels, cholesterol, and micronutrient concentrations are routinely measured to assess dietary intake and its relationship with health outcomes. However, these biomarkers often exhibit high within-subject variability due to factors such as dietary habits, metabolic differences, and daily fluctuations. For example, a study on the relationship between vitamin D levels and bone health must account for the day-to-day variability in vitamin D concentrations to accurately assess the impact. Our calculators help researchers design studies that account for this variability, ensuring that sample sizes are sufficient to detect meaningful associations even without formally adjusting effect estimates for biases due to measurement error.

2.3 Example 3: Longitudinal Studies on Chronic Disease Progression

Longitudinal studies tracking the progression of chronic diseases like diabetes and cardiovascular diseases over time require repeated measurements of various biomarkers. The ICC becomes particularly relevant in these studies, as it reflects the consistency of biomarker measurements within individuals across multiple time points, if we assume that there is no time-trend in the studied exposure. For instance, in a study investigating the progression of diabetes, researchers might measure HbA1c levels quarterly to monitor changes. Variability in these measurements can impact the study's ability to detect true changes in disease status. Using our calculators, researchers can estimate the number of repeated measurements needed to achieve reliable results, balancing the trade-off between study duration, sample size, and measurement precision, number of persons studied, and measurement precision (number of repeated measurements per time point and person).

2.4 Example 4: Environmental Exposure and Reproductive Health

Research on environmental exposures and reproductive health outcomes, such as fertility rates and birth defects, often involves measuring low-level contaminants like phthalates and BPA. These studies face challenges due to the low levels of contaminants and the high variability in individual exposure. For example, a study on the effects of BPA on fertility may need to measure BPA levels in urine samples repeatedly to account for variability due to dietary and environmental sources. By applying our sample size calculators, researchers can design studies that are adequately powered to detect associations between exposure levels and reproductive outcomes, even in the presence of non-ignorable measurement error.

2.5 Data Example 1: EPA Air Quality Monitoring Data

The Environmental Protection Agency (EPA) monitors air quality across various regions, providing detailed data on pollutant levels like $PM_{2.5}$. These data are crucial for studies assessing the health impacts of air pollution. By applying our calculators to EPA

data, researchers can design studies that account for the variability in pollutant levels, ensuring robust findings regarding their health effects.

2.6 Data Example 2: NHANES Biomarker Data

The National Health and Nutrition Examination Survey (NHANES) provides a rich dataset of biomarker measurements across a representative sample of the U.S. population. These data include repeated measurements of blood pressure, cholesterol, and glucose levels, among others. Using NHANES data, we can demonstrate how our calculators can optimize sample size and measurement intervals to achieve desired confidence levels and detect significant health associations.

2.7 Data Example 3: Longitudinal Cohort Data from the Framingham Heart Study

The Framingham Heart Study, a long-term ongoing cardiovascular cohort study, provides extensive data on various health biomarkers measured repeatedly over decades. This dataset is ideal for illustrating how our calculators can be used to determine the number of measurements needed to detect changes in biomarkers like cholesterol and blood pressure over time, considering both within- and between-subject variability.

2.8 Data Example 4: Birth Cohort Studies on Environmental Exposures

Birth cohort studies, such as the Avon Longitudinal Study of Parents and Children (ALSPAC), collect data on environmental exposures and health outcomes from pregnancy through childhood. These datasets include repeated measurements of contaminants like phthalates and BPA in urine. By using our calculators, researchers can optimize study designs to account for the high variability in these exposures, ensuring that sample sizes are sufficient to estimate the hypothesized effects on health outcomes with desired (planned) precision and control for type 1 and 2 errors.

The motivating examples and data provided illustrate the critical importance of accounting for variability and measurement error *during the design of* epidemiological

studies. By using our suite of calculators, researchers can design more accurate and reliable studies, leading to better-informed public health decisions and improved outcomes.

3 Methods

This section describes the development of several calculators designed to address key challenges in epidemiological research, particularly those involving measurement variability and sample size determination. These calculators apply statistical theory from the literature on measurement errors in linear and logistic regression modeling to provide accurate and reliable estimates for study design parameters. Here, we outline the methods used in developing these calculators, along with explanations and examples of their applications.

3.1 The "Number of Repeats" Calculator" Quantifies Repeats Required to Achieve a Desired Validity Coefficient (https://scipinion.shinyapps.io/RepeatsForDVC/)

A researcher planning a study's budget and logistics wants to know how many samples (e.g., urine void samples) must be collected from an individual to achieve a desired level of validity (a measure of how close the measured value is expected to be to the true value) in the person's true mean exposure (e.g. typical urine concentration during specified time) level. The Number of Repeats calculator estimates the number of repeated measurements (m) needed to achieve a desired reliability, R_m (see ρ^2_{xz} above) given an intraclass correlation coefficient (ICC). The validity coefficient is calculated as $|R_m^{0.5}|$ and indicates the correlation between observed and true values, reflecting the precision of measurements if classical additive measurement error model holds.

The key formula used in this calculator is derived from Fleiss (1986), expression (1.31) on page 15:

$$m = (R_m \times (1 - \text{ICC})) / (\text{ICC} \times (1 - R_m))$$

where:

- R_m is the desired reliability, a square of the validity coefficient.
- ICC is the intraclass correlation coefficient.

The calculated value of *m* is then rounded up to the nearest integer to ensure it is a whole number.

Example:

Consider a study where the desired validity coefficient is 0.85, and the ICC is 0.3. The reliability is $0.85^2 = 0.7226$. Using the formula, the number of repeats needed is calculated as follows: m = (0.7226 × (1 - 0.3)) / (0.3 × (1 - 0. 0.7226)) = 6.08 Rounding up, we get *m* = 7.

3.2 Sample Size Calculator for Mean with Desired Margin of Error (https://scipinion.shinyapps.io/SampleSizeMOE/)

Suppose we design a study that estimates population mean of biomarker level with precision that is sufficient for deciding about risk that such exposures entail. This calculator determines the sample size (*n*) required to achieve a specified margin of error (d) for a given standard deviation (σ) and confidence level (1 - α), where α is Type 1 error rate.

The formula for calculating the sample size is:

$$n = (\sigma \times z_{\alpha/2}/d)^2$$

where:

- $z_{\alpha/2}$ is the critical value from the standard normal distribution for the desired confidence level.
- σ is the standard deviation of the population.
- d is the desired margin of error.

Example:

For a standard deviation of 2, a desired margin of error of 0.3, and a 95% confidence level: $z_{0.025}$ = 1.96, n = ((1.96 × 2) / 0.3)^2 = (3.92 / 0.3)^2 = 163

3.3 Sample Size and Minimum Detectable Effect (MDE) Tradeoff Calculator for Linear Regression (https://scipinion.shinyapps.io/LinearRegressionN/ and https://scipinion.shinyapps.io/LinearRegressionM/)

Imagine that a researcher is designing a study aim to estimate relationship between exposure that exists on a continuous scale that can be transformed to follow normal distribution (as is typical) and some health outcome measured also on a continuous scale, like Z-score of birthweights standardized for gestational age and sex of the child. Such a study would have to demonstrate that it can achieve sufficient power and not place undue burden on participants (e.g. by drawing their blood, collecting urine, hair, etc.) to be deemed ethical. How many subjects should one enroll in (e.g. newborns, *n*) and how often should one measure biomarker of exposure of interest in the mother during pregnancy at the time of etiological significance (*m*)? This calculator evaluates the tradeoff between sample size (*n*), number of repeated measurements per subject (*m*) and the minimum detectable effect (MDE = d_a) in linear regression models ($Y|\overline{Z}_j$), accounting for classical additive measurement error.

The primary formula used is:

$n_Z = s^2 \times (z_\beta + z_{\alpha/2})^2/(R_m \times V_b \times d_a^2)$

Where:

- z_β is the critical value for the desired Type 2 error (1-power).
- $z_{\alpha/2}$ is the critical value for the significance level.
- s^2 is the variance of the regression errors, estimated as (V_Y - $V_b \times d_a^2$), where V_y is the variance of the dependent variable (Y).
- R_m is reliability as defined above = $V_b/(V_b + V_w/m)$
- V_b is the between-subject variance of exposure.
- V_w is the within-subject variance of exposure.

- m is the number of repeated measurements per subject used to estimate exposure.
- d_a is the MDE when the null hypothesis is slope = 0.

Example:

For MDE = 0.5, variance of Y of 3, within-subject variance (V_w) of 3, ICC of 0.4, m = 2, α = 0.05, and power of 0.9: $z_{0.025}$ = 1.96, $z_{0.1}$ = 1.28, V_b = (3 × 0.4) / (1 - 0.4) = 2, s^2 = (3 - 2 × 0.5^2) = 2.5, R_m = 2/(2+3/2) = 0.57, n_Z = (2.5 × $(1.96 + 1.28)^2$) / (0.57 × 2 × 0.5^2) = 92.

3.4 Sensitivity Analysis Explorer for Logistic Regression (https://scipinion.shinyapps.io/SensitivityAnalysisExplorer/)

Imagine that a researcher is designing a study to estimate the relationship between a continuous exposure variable that can be transformed to follow a normal distribution (as is typical) and some health outcome that is captured as either present or absent (e.g. a specific birth defect). Such a study would have to demonstrate that it can achieve sufficient power and not place undue burden on participants (e.g. by drawing their blood, collecting urine, hair, etc. too frequently) to be deemed ethical. How many subjects, *n*, should one enroll (e.g. newborns) and how many times should one measure biomarker of exposure of interest in the mother during pregnancy at the time of etiological significance (*m*)? This calculator examines the sensitivity of sample size and power to measurement error in logistic regression models, typically employed for binary outcomes in epidemiology. Unlike linear regression, there is no closed form solution for bias in the logistic regression under classical measurement error model and we therefore use numerical simulations to obtain the answer (Gustafson, 2004). This calculator simulates the power and bias of parameter estimates under varying conditions of between-person variance (V_b), within-person variance (V_w), true odds ratio (OR), and baseline probabilities (p_0).

The simulation process involves generating data based on specified parameters and fitting logistic regression models to estimate the power and bias.

Example:

For a simulation with V_b = 6, V_w = 3, OR = 1.1, p_0 = 0.1, sample size = 200, and m = 5, the simulation runs multiple iterations to estimate the power and bias. The results indicate the percentage of simulations where the p-value is less than 0.05 (power) and the average relative bias of the estimated OR.

The calculators described in this section provide researchers with easy-to-use computational aids to design robust epidemiological studies, help ensure adequate sample sizes, and better understand the impact of measurement error. With these methods, researchers can make better-informed decisions to promote more accurate and reliable public health findings.

4 Results

This section presents illustrative results obtained by using the calculators, focusing on findings for various scenarios. The figures illustrate key insights and demonstrate the functionality of the calculators, emphasizing the relationship between sample size, measurement error, and study power.

4.1 Number of Repeats Needed for Desired Validity Coefficient

The first calculator determines the number of repeated measurements required to achieve a desired validity coefficient given an ICC (Figure 2).

Key Findings:

- The number of repeats needed decreases as the ICC increases.
- Fewer resources are needed to study exposures with high as opposed to low ICC

4.2 Sample Size Calculator for Desired Margin of Error in Estimating Exposure Level

This calculator estimates the sample size required to achieve a specified margin of error for a given standard deviation and confidence level (Figure 3).

Key Findings:

- A higher standard deviation or lower desired margin of error requires a larger sample size.
- When we need more precise estimates and exposure is more variable one can anticipate more resource-intensive research, far more so than if there was no intrinsic variability over time within a person.

4.3 Sample Size and MDE Tradeoff Calculation for Linear Regression

This calculator evaluates the tradeoff between sample size and the minimum detectable effect (MDE) in linear regression models with classical additive measurement error (Figure 4).

Key Findings:

- A smaller MDE requires a larger sample size to achieve the same power compared to larger MDE.
- For exposures with low ICC, the number of repeated measurements per subject needed to detect realistic MDE can be unrealistic when there is a limited number of subjects.
- Increasing the number of subjects may be a more practical way to achieve desired MDE than boosting the number of times a subject is asked to provide biological samples.

4.4 Sample Size and MDE Tradeoff Calculation for Logistic Regression

The sensitivity analysis explorer examines the impact of measurement error on power and bias in logistic regression models. It simulates different conditions of between-person and within-person variance, true odds ratios, and baseline probabilities. Simulation allows one to answer the same questions as for linear regression (above) (Figure 5).

Key Findings:

- The power of detecting true effects increases with number of subjects (*n*) and repeated measurements per subject (*m*).
- Bias in the odds ratio decreases with an increasing number of repeated measurements per subject, when the total number of subjects is fixed.
- The same general patterns of trade-off are apparent for linear and logistic regression.

An unexpected finding from the logistic regression is that bias tends to increase with number of subjects for a fixed number of repeats. This seemingly counterintuitive result suggests that, in the presence of measurement errors, larger sample sizes may not always lead to more accurate estimates. Instead, the accumulation of measurement errors can exacerbate bias. It is well known to statisticians that, in the presence of measurement error, the estimate obtained with very large samples will be biased and converge to a constant. On the other hand, when sample sizes are “small” under the same conditions, the estimates can vary wildly, creating distribution of estimates that, on average, may well be greater than the large sample expectations. This is an interesting area for further investigation. Our observations are based on a very limited number of simulations. It stresses the value of our approach that relies on simulations of realistic, rather than theory-idealized scenarios (such as near-infinite sample size and the similarity of the impact of measurement error in linear and logistic regressions).

These examples illustrate the tradeoffs between sample size, measurement error, and statistical power. Using these tools, researchers can make better-informed decisions to help design more robust and reliable epidemiological studies.

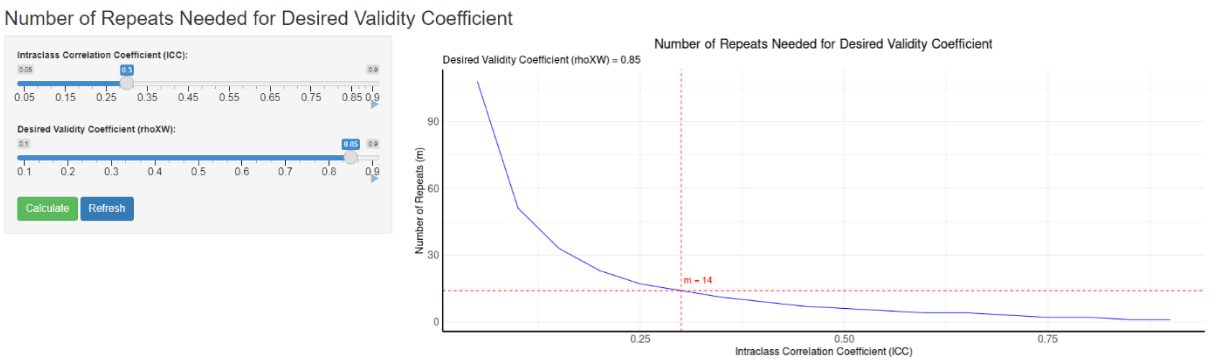

Figure 2: Results from Calculator #1: Number of repeats needed for desired validity coefficient.

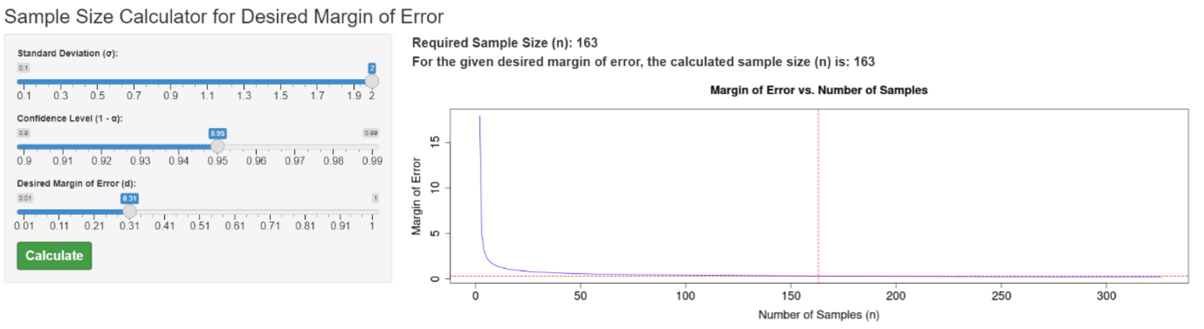

Figure 3: Results from calculator #2: Sample size calculator for desired margin of error

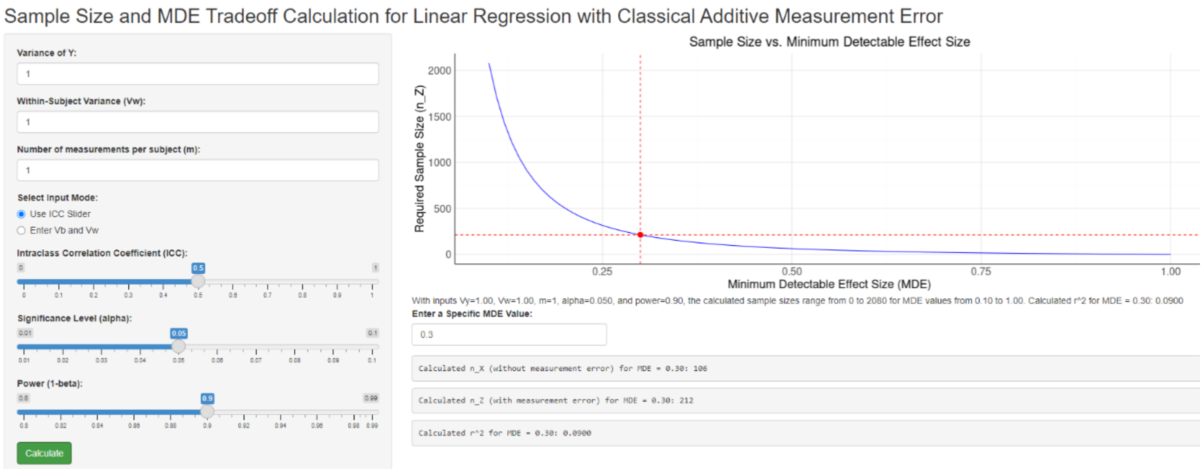

Figure 4: Results from calculator #3: Sample size and MDE tradeoff calculation for linear regression with classical additive measurement error

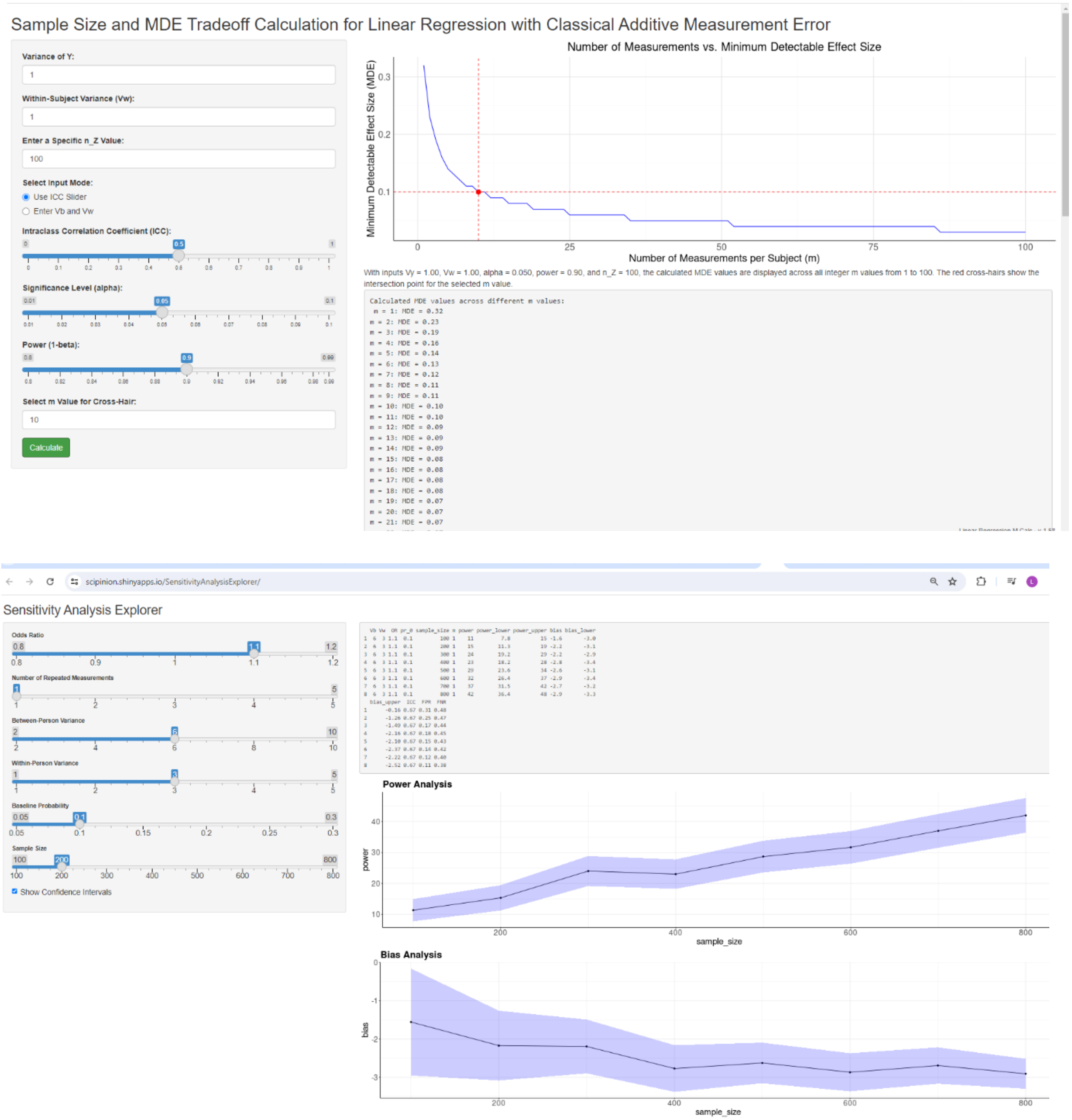

Figure 5: Results from calculator #4:

5 Discussion

The results from our suite of calculators underscore the significant impact of measurement error and variability on study design and outcomes in epidemiological research. This discussion focuses on the implications of our findings, particularly the necessity of an adequately large number of subjects (*n*) and repeated measurements

per subject (*m*) to achieve desired minimum detectable effects (MDEs) for realistic intraclass correlation coefficient (ICC) values.

5.1 Implications for Study Design

1. **Adequate Number of Measurements (*n* × *m*) and Measurement Precision:** Our calculators demonstrate that achieving precise and reliable results often requires larger measurement efforts (many persons *n* with multiple measurements *m* each) than traditionally believed, when measurement error in exposure is not considered. This is particularly evident in studies with high within-subject variability (low ICC). For example, the number of measurements and participants required to detect a small MDE in linear regression models with classical measurement error can be orders of magnitude larger than if the true exposure was known. Researchers must carefully balance the desired level of precision with practical constraints such as cost, time, and available resources.
2. **Number of Repeated Measurements (*m*):** The necessity of repeated measurements to enhance the desired validity of biomarker data is highlighted by our calculator for number of repeats needed to obtain average values per person with sufficiently high correlation with true values. As ICC values decrease, the number of repeats needed increases exponentially. This finding emphasizes that in studies involving exposures with low ICC, obtaining multiple measurements per subject is critical to reducing measurement error and achieving the desired validity coefficient. Alternatively, researchers can explore strategies to increase the ICC (e.g., 24-hour urine void composites, etc.), at the expense of altering the dose (and thus the hypothesis exposure-response association) that these biomarkers estimate.
3. **Impact of Measurement Error on Bias in Linear and Logistic Regressions:** We observed that more subjects (*n*) and more repeated measurements per subjects (*m*) are needed to achieve desired power than one would expect if there was no measurement error (i.e. ICC = 1). Reduction in bias due to measurement error is achieved by increasing number of repeated measurements per subject (*m*) and there may be a hazard of aggregating bias if number of subjects (*n*) is

increased at the expense of reduction in measurement error by increasing the number of repeated measurements per subject (*m*). However, there are practical and ethical limits to how may measurements of biomarkers one may obtain from a person, in which case one would be advised to adjust analyses for the remaining unacceptable measurement error; methods to do so are acceptable though collaboration with statisticians (Keogh et al. 2020, Shaw et al. (2020).

4. **Tradeoffs Between Power, Number of Measurements, and Measurement Error:** Our calculators illustrate the complex interplay between power, number and allocation of measurements, and measurement error. For instance, while increasing the number of subjects (*n*) generally enhances the power to detect true effects, it can also amplify the bias due to measurement error if not properly addressed by a large number of repeated measurements per person (*m*). Researchers must carefully consider these tradeoffs when designing studies, ensuring that measurements are allocated to achieve the desired power without introducing undue bias from measurement errors.

5.2 Practical Considerations

1. **Cost and Feasibility:** While a larger number of subjects (*n*) and repeated measurements per subject (*m*) can improve study accuracy and validity, they also entail higher costs and logistical challenges. Researchers must weigh these factors against the benefits of increased precision and reliability. In some cases, it may be more practical to focus on adjusting for bias due to measurement error that is unavoidable. The cost of collaboration with statisticians who can adjust for measurement error may well be far below the cost of recruiting a large sample of people who need to be very committed to multiple invasive procedures (in the case of biomarkers).

2. **Realistic ICC Values:** Understanding and accurately estimating ICC values is crucial for planning studies. Realistic ICC values, derived from pilot studies or existing literature, should inform study design to ensure that the planned methodologies are both effective and feasible.

3. **Regulatory and Policy Implications:** The findings from our calculators have important implications for regulatory bodies and policymakers. Ensuring that epidemiological studies are designed with adequate sample sizes and measurement strategies can lead to more reliable evidence for public health decisions. This, in turn, can inform regulations and interventions aimed at mitigating health risks associated with environmental exposures, dietary habits, and other factors.

These findings emphasize the need for adequately large numbers of participants and repeated measurements on them to achieve desired MDEs for realistic ICC values. By applying the calculators, researchers can design more robust and reliable epidemiological studies, ultimately contributing to better-informed public health policies and interventions. Addressing measurement error and variability through thoughtful study design is essential for advancing our understanding of complex health relationships and improving population health outcomes. Understanding how measurement errors limit what one can learn from data can also stimulate collaboration with statisticians who know how to compensate for measurement errors during data analysis.

6 Conclusions

The development and application of our statistical calculators reveal several key insights and challenges in the design and analysis of epidemiological studies. Our findings lead to the following conclusions:

1. **Limitations of Current Practice with Small Number of Repeated Measurements per Person:** Current practices that utilize small numbers of repeated measurements per subject (m) can be misleading, especially in studies with low intraclass correlation coefficients (ICCs). When m is small, measurement error can noticeably distort the validity of the results. This distortion arises because small m values fail to capture the within-subject variability adequately, leading to a tendency to underestimate the true effect sizes and an overestimation of the study's precision (although one can never tell just what such impact is in any individual analysis).

2. **Challenges with Large Number of Subjects (n):** While increasing the number of participants (n) generally enhances the power of a study to detect associations, our analysis for logistic regression and general considerations for any regression model indicate that large studies with poorly controlled measurement error introduce their own set of problems. Particularly in the presence of large measurement error there is an apparently paradoxical increase in bias with the increase in number of subjects. In such studies, the effect estimate is biased with an ever-increasing precision, such that eventually one has absolute certainty about effect estimate that is far away from its true value (Gustafson 2010). This may be sufficient for obtaining qualitative answers, but not for risk analysis that requires quantification of exposure-response gradient. However, our limited observations about precise nature of bias we observed require confirmation through more theoretical and numerical research.

3. **Utility of Calculators in Study Planning:** Our suite of calculators provides valuable tools for planning realistic and effective epidemiological studies. By helping researchers estimate the appropriate number of repeated measurements (*m*) and the required number of participants (*n*) to achieve desired minimum detectable effects (MDEs), these calculators facilitate more accurate and reliable study designs. They allow for a nuanced understanding of the trade-offs between sample size, measurement error, and study power, enabling researchers to make informed decisions that balance precision with practical constraints.

4. **Cost Considerations:** Achieving the desired MDEs with adequately large *m* and *n* values can be costly and logistically challenging. While the benefits of increased precision and reliability are clear, the financial and logistical burdens must be considered. Researchers must weigh these costs against the potential for more accurate and valid results, exploring alternative strategies such as selecting populations with large contrast in individual exposures (as is often done in occupational epidemiology, see Tielemans et a. 1998) and statistical adjustments for measurement error through collaboration with statisticians. Work of Armstrong (1996) of "asymptotic relative efficiency" when $m = 1$ can be

extended to *m* >1 in order to explicitly calculate cost trade-offs for a wider range of epidemiologic designs. Such research is similar to calculations used to decide whether resort to measurements of external exposure or biomarkers, e.g. Rappaport et al. (1995).

The findings from our calculators highlight the complexity of designing robust epidemiological studies. Current practices with small number of repeated measurements (often dictated by logistics and budgets) can lead to misleading results, and increasing number of participants without addressing measurement error can exacerbate bias in statistics that assume no measurement error (like linear and logistic regression implemented in standard software). Our calculators provide a means to navigate these challenges, enabling researchers to plan studies that are both feasible and scientifically rigorous. By carefully considering the trade-offs and costs involved, researchers can design studies that yield reliable and actionable insights, ultimately contributing to better public health outcomes.

7 References

Armstrong, B. G. (1996). "Optimizing power in allocating resources to exposure assessment in an epidemiologic study." Am.J.Epidemiol. 144: 192-197.

Fleiss, J.L. 1986. The Design and Analysis of Clinical Experiments. John Wiley & Sons, Inc. New York, NY. DOI:10.1002/9781118032923

Gustafson, P. (2004). Measurement Error and Misclassification in Statistics and Epidemiology, Chapman & Hall/CRC Press.

Gustafson P. Bayesian inference for partially identified models. Int J Biostat. 2010;6(2):Article 17. doi: 10.2202/1557-4679.1206. PMID: 21972432.

Kim HM, Richardson D, Loomis D, Van Tongeren M, Burstyn I. Bias in the estimation of exposure effects with individual- or group-based exposure assessment. J Expo Sci Environ Epidemiol. 2011 Mar-Apr;21(2):212-21. doi: 10.1038/jes.2009.74

Keogh, R. H., P. A. Shaw, P. Gustafson, R. J. Carroll, V. Deffner, K. W. Dodd, H. Kuchenhoff, J. A. Tooze, M. P. Wallace, V. Kipnis and L. S. Freedman (2020). "STRATOS guidance document on measurement error and misclassification of variables in observational epidemiology: Part 1-Basic theory and simple methods of adjustment." Stat Med 39(16): 2197-2231.

Koch HM, Aylward LL, Hays SM, Smolders R, Moos RK, Cocker J, Jones K, Warren N,

LaKind JS, Idri F, Naiman DQ, Verner MA. Biomonitoring and Nonpersistent Chemicals-Understanding and Addressing Variability and Exposure Misclassification. Curr Environ Health Rep. 2019 Mar;6(1):16-21. doi: 10.1007/s40572-019-0227-2. PMID: 30701410.

Levy L, Bevan R. Inter- and intra-individual variation in urinary biomarker concentrations over a 6-day sampling period. Part 2: personal care product ingredients. Toxicol Lett. 2014 Dec 1;231(2):261-9. doi: 10.1016/j.toxlet.2014.06.023. Epub 2014 Jun 20. PMID: 24956590.

Paustenbach D, Galbraith D. Biomonitoring and biomarkers: exposure assessment will never be the same. Environ Health Perspect. 2006 Aug;114(8):1143-9. doi: 10.1289/ehp.8755. PMID: 16882516; PMCID: PMC1552022.

Preau, J. L., Jr., Wong, L. Y., Silva, M. J., Needham, L. L., and Calafat, A. M. 2010. Variability over 1 week in the urinary concentrations of metabolites of diethyl phthalate and di(2-ethylhexyl) phthalate among eight adults: an observational study. Environ. Health Perspect. 118: 1748–1754.

Preller L, Kromhout H, Heederik D, Tielen MJ. Modeling long-term average exposure in occupational exposure-response analysis. Scand J Work Environ Health. 1995 Dec;21(6):504-12. doi: 10.5271/sjweh.67.

Rappaport SM and Spear RC. Physiological dampening of exposure variability during brief periods. Annals of Occupational Hygiene. 1988 32(1): 21-33.

Rappaport SM, Symanski E, Yager JW, Kupper LL. The relationship between environmental monitoring and biological markers in exposure assessment. Environ Health Perspect. 1995 Apr;103 Suppl 3(Suppl 3):49-53.

Sexton, K., Needham, L. L., and Pirkle, J. L. 2004. Human biomonitoring of environmental chemicals. Am. Sci. 92: 38–45.

Shaw, P. A., P. Gustafson, R. J. Carroll, V. Deffner, K. W. Dodd, R. H. Keogh, V. Kipnis, J. A. Tooze, M. P. Wallace, H. Kuchenhoff and L. S. Freedman (2020). "STRATOS guidance document on measurement error and misclassification of variables in observational epidemiology: Part 2-More complex methods of adjustment and advanced topics." Stat Med 39(16): 2232-2263.

Smolders R, Koch HM, Moos RK, Cocker J, Jones K, Warren N, Levy L, Bevan R, Hays SM, Aylward LL. Inter- and intra-individual variation in urinary biomarker concentrations over a 6-day sampling period. Part 1: metals. Toxicol Lett. 2014 Dec 1;231(2):249-60. doi: 10.1016/j.toxlet.2014.08.014. Epub 2014 Aug 13. PMID: 25128590.

Tielemans E, Kupper LL, Kromhout H, Heederik D, Houba R. Individual-based and group-based occupational exposure assessment: some equations to evaluate different

strategies. Ann Occup Hyg. 1998 Feb;42(2):115-9. doi: 10.1016/s0003-4878(97)00051-3.

8 Appendix: Instructions and Source Code

8.1 User's Guide for Calculator for Mean with Desired Margin of Error

8.1.1 Introduction

When conducting a study or experiment, it is crucial to determine the sample size needed to ensure that the results are statistically significant (i.e., confidently distinguishable from chance results) and that they are precise enough to be useful. One common objective is to ensure that the 95% confidence interval for the estimated mean of a population based on a sample is acceptably small – that is, that its width reflects a user-specified desired “margin of error” (MOE), such as ±5% or ±2.5%. This exposition will guide you through the process of calculating the required sample sizes.

8.1.1.1 Terminology and Formula

- Standard Deviation (sigma): A measure of the amount of variation or dispersion in a set of values around their mean (i.e., average) value.
- Margin of Error (d): The maximum acceptable difference (at the specified confidence level) between (a) the sample mean; and (b) the true population mean that the sample mean estimates
- Confidence Level: The proportion of times that an interval constructed from sample data according to specified rules (such as adding and subtracting a specified number of sample standard deviations from the sample mean) is expected to contain the true population mean that is being estimated.

The formula to calculate the required sample size (*n*) for a given margin of error (*d*) is:

n = (z * sigma / d)^2

Where:

- z is the z-score corresponding to the desired confidence level (e.g., 1.96 for 95% confidence when using the normal distribution).
- sigma is the standard deviation of the population.
- d is the desired margin of error.

If the population standard deviation (sigma) is unknown and must be estimated from the sample data, the t-score from the t-distribution is used instead of the z-score. The t-score depends on both the desired confidence level and the degrees of freedom (which is related to the sample size). For large samples, the t-score approximates the z-score. For this calculator, we treat sigma as a user-specified input, so its value is known.

8.1.2 Accessing the Application

The Calculator for Mean with Desired Margin of Error is available online as a Shiny application at https://scipinion.shinyapps.io/SampleSizeMOE/). You can access it directly through your web browser without the need to install R or RStudio.

8.1.3 Using the Application

8.1.3.1 Interface Overview

The Sample Size Calculator for Desired Margin of Error application interface consists of a sidebar panel with various input controls and a main panel displaying the plot containing the outputs.

8.1.3.2 Input Controls

- **Standard Deviation (σ)**: Use the slider to adjust the standard deviation, which ranges from 0.1 to 2.
- **Confidence Level (1 - α)**: Use the slider to set the confidence level, which ranges from 90% to 99%.
- **Desired Margin of Error (d)**: Use the slider to set the desired margin of error, which ranges from 0.01 to 1.
- **Calculate Button**: Press to calculate the required sample size.
- **Refresh Button**: Press to reset the input values to their defaults.
- **Result Text**: Displays the calculated sample size needed.

8.1.3.3 Output Display

- **Margin of Error Plot**: Displays a plot showing the relationship between the number of samples and the margin of error based on the input values.
- **Calculated Sample Size**: Provides a textual output of the calculated sample size based on the input values.

8.1.4 Steps to Use the Calculator for Mean with Desired Margin of Error

1. **Adjust Input Parameters**: Use the input controls on the sidebar to set the desired values for standard deviation (σ), confidence level, and desired margin of error (d).
2. **Calculate**: Press the "Calculate" button to compute the required sample size.
3. **View Results**: The plot and result text will update automatically based on the selected inputs. The plot will show the relationship between the number of samples and the margin of error, highlighting the specific values you have entered.

8.1.5 Example Calculation

Let's use the following values for our example:

- Standard Deviation (sigma): 2
- Desired Margin of Error (d): 0.5
- Confidence Level: 95 percent

For a 95 percent confidence level, the z-score is approximately 1.96.

Step-by-Step Calculation

1. Identify the z-score: For a 95 percent confidence level, the z-score is approximately 1.96.
2. Plug in the values: Substitute the values into the formula: n = (1.96 * 2 / 0.5) ^ 2
3. Calculate the numerator: 1.96 * 2 = 3.92
4. Divide by the margin of error: 3.92 / 0.5 = 7.84
5. Square the result: 7.84 ^ 2 = 61.4656
6. Round up to the nearest whole number: The required sample size (n) should be a whole number, so we round up: n = approximately 62

8.1.5.1 Interpretation

For this example, you would need to collect a sample of at least 62 observations to ensure that the 95 percent confidence interval for the mean is within 0.5 units of the true mean, given a standard deviation of 2.

8.1.5.2 Practical Implications

This calculation helps researchers determine the necessary sample size to achieve their desired precision, which is critical for planning studies and ensuring statistically valid results. Adequate sample size calculation is also essential for justifying the study design to peer reviewers and ensuring the reliability and validity of the findings.

Source code for Sample Size Calculator for Mean with Desired Margin of Error (https://scipinion.shinyapps.io/SampleSizeMOE/)

```
library(shiny)

# Define UI for application
ui <- fluidPage(
  titlePanel("Sample Size Calculator for Desired Margin of Error"),

  tags$head(
    tags$style(HTML("
      .btn-success {
        font-size: 20px;
        font-weight: bold;
      }
      .irs-single, .irs-bar, .irs-from, .irs-to, .irs-min, .irs-max, .irs-grid-text {
        font-size: 16px;
      }
      #sample_size, #sample_size_box {
        font-size: 20px;
        font-weight: bold;
      }
    "))
  ),

  sidebarLayout(
    sidebarPanel(
      sliderInput("std_dev", "Standard Deviation (σ):", min = 0.1, max = 2, value = 2, step = 0.1),
      sliderInput("conf_level", "Confidence Level (1 - α):", min = 0.9, max = 0.99, value = 0.95,
step = 0.01),
      sliderInput("margin_error", "Desired Margin of Error (d):", min = 0.01, max = 1, value = 0.5,
step = 0.01),
      actionButton("calculate", "Calculate", class = "btn-success")
    ),

    mainPanel(
      textOutput("sample_size"),
      textOutput("sample_size_box"),
      plotOutput("plot")
    )
  )
)

# Define server logic
server <- function(input, output) {
  observeEvent(input$calculate, {
    conf_level <- input$conf_level
    sigma <- input$std_dev
    d <- input$margin_error
    alpha <- 1 - conf_level
```

```
    # Use z-value for calculation
    z_value <- qnorm(1 - alpha / 2)
    n <- ceiling((z_value * sigma / d) ^ 2)

    output$sample_size <- renderText({
      paste("Required Sample Size (n):", n)
    })

    output$sample_size_box <- renderText({
      paste("For the given desired margin of error, the calculated sample size (n) is:", n)
    })

    output$plot <- renderPlot({
      sample_sizes <- seq(1, n * 2, by = 1)
      margins <- z_value * sigma / sqrt(sample_sizes)

      plot(sample_sizes, margins, type = "l", col = "blue",
           xlab = "Number of Samples (n)",
           ylab = "Margin of Error",
           main = "Margin of Error vs. Number of Samples",
           cex.lab = 1.5, cex.main = 1.5, cex.axis = 1.5)
      abline(h = d, col = "red", lty = 2)
      abline(v = n, col = "red", lty = 2)
    })
  })
}

# Run the application
shinyApp(ui = ui, server = server)
```

8.2 User's Guide for One-Sample Calculator Using Fleiss Formula

8.2.1 Introduction

The One-Sample Calculator Using Fleiss Formula is a Shiny application designed to help users determine the number of repeated measurements needed to achieve a desired validity coefficient for a given intraclass correlation coefficient (ICC). This tool is useful for researchers and practitioners who need to ensure their measurements are reliable and valid.

8.2.2 Accessing the Application

The One-Sample Calculator is available online as a Shiny application at https://scipinion.shinyapps.io/RepeatsForDVC/). You can access it directly through your web browser without the need to install R or RStudio.

8.2.3 Using the Application

8.2.3.1 Interface Overview

The One-Sample Calculator application interface consists of a sidebar panel with various input controls and a main panel displaying the plot containing the outputs.

8.2.3.2 Input Controls

- **Intraclass Correlation Coefficient (ICC)**: Use the slider to adjust the ICC, which ranges from 0.05 to 0.9.
- **Desired Validity Coefficient (rhoXW)**: Use the slider to set the desired validity coefficient, which ranges from 0.1 to 0.975.
- **Calculate Button**: Press to calculate the number of repeated measurements needed.
- **Refresh Button**: Press to reset the input values to their defaults.
- **Result Text**: Displays the calculated number of repeated measurements needed and the exact value before applying the ceiling function.

8.2.3.3 Output Display

- **Error Plot**: Displays a plot showing the relationship between the ICC and the number of repeated measurements needed to achieve the desired validity coefficient.
- **Calculated Number of Repeats**: Provides a textual output of the calculated number of repeats needed based on the input values.

8.2.4 Steps to Use the One-Sample Calculator Application

4. **Adjust Input Parameters**: Use the input controls on the sidebar to set the desired values for ICC and the desired validity coefficient (rhoXW).
5. **Calculate**: Press the "Calculate" button to compute the required number of repeated measurements.

6. **View Results**: The plot and result text will update automatically based on the selected inputs. The plot will show the relationship between ICC and the number of repeats, highlighting the specific values you have entered.
7. **Refresh**: Press the "Refresh" button to reset the sliders to their default values.

8.2.5 Example

To determine the number of repeated measurements needed to achieve a desired validity coefficient of 0.85 with an ICC of 0.3:

1. Set the **Intraclass Correlation Coefficient (ICC)** to 0.3 using the slider.
2. Set the **Desired Validity Coefficient (rhoXW)** to 0.85 using the slider.
3. Press the **Calculate** button to see the number of repeats needed.
4. The result text will display the calculated number of repeats needed and the exact value before ceiling.
5. The plot will show a dashed red line at the input ICC and another at the calculated number of repeats, with annotations indicating the values.

The One-Sample Calculator should produce output that helps you understand the relationship between ICC, validity coefficient, and the number of repeated measurements needed to ensure reliable and valid results.

```
# Source code for simple one-sample calculator using Fleiss formula, 6-17-24
library(shiny)
library(ggplot2)

# Define UI
ui <- fluidPage(
  tags$head(
    tags$style(HTML("
      .slider-animate-button {
        font-size: 16px;
      }
      .irs-grid-text, .irs-from, .irs-to, .irs-single, .irs-bar, .irs-line {
        font-size: 16px;
      }
      .shiny-text-output {
        font-size: 16px;
      }
      .btn-success, .btn-primary {
        font-size: 16px;
      }
    "))
  ),
  titlePanel("Number of Repeats Needed for Desired Validity Coefficient"),
  sidebarLayout(
    sidebarPanel(
      sliderInput("icc", "Intraclass Correlation Coefficient (ICC):", min = 0.05, max = 0.9, value = 0.3, step = 0.05,
                  animate = animationOptions(interval = 300, loop = FALSE), width = '100%'),
      sliderInput("rhoXW", "Desired Validity Coefficient (rhoXW):", min = 0.1, max = 0.975, value = 0.85, step =
0.05,
                  animate = animationOptions(interval = 300, loop = FALSE), width = '100%'),
      actionButton("calculate", "Calculate", class = "btn-success"),
      actionButton("refresh", "Refresh", class = "btn-primary"),
      textOutput("result")
    ),
    mainPanel(
      plotOutput("errorPlot", height = "500px")
    )
  )
)

# Define server logic
server <- function(input, output, session) {
  reactiveData <- reactiveValues(data = NULL)

  # Calculate the number of repeats needed
  calculateRepeats <- function(icc, rhoXW) {
    numerator <- rhoXW^2 * (1 - icc)
    denominator <- icc * (1 - rhoXW^2)
    m <- numerator / denominator
    ceiling_m <- ceiling(m)  # Ceiling to ensure m is an integer
    list(m = m, ceiling_m = ceiling_m)
  }

  observeEvent(input$calculate, {
    req(input$icc > 0 & input$rhoXW > 0)
```

```
    calculation <- calculateRepeats(input$icc, input$rhoXW)
    calculated_m <- calculation$ceiling_m
    output$result <- renderText({
      paste("Calculated number of repeats needed: ", calculated_m,
            "\nExact value before ceiling: ", calculation$m)
    })
  })

  observeEvent(input$refresh, {
    updateSliderInput(session, "icc", value = 0.3)
    updateSliderInput(session, "rhoXW", value = 0.85)
    output$result <- renderText({ "" })
  })

  output$errorPlot <- renderPlot({
    icc_values <- seq(0.05, 0.9, by = 0.05)
    rhoXW_value <- input$rhoXW
    m_values <- sapply(icc_values, function(icc) {
      calculateRepeats(icc, rhoXW_value)$ceiling_m
    })

    df <- data.frame(ICC = icc_values, m = m_values)

    p <- ggplot(df, aes(x = ICC, y = m)) +
      geom_line(color = "blue") +
      theme_minimal() +
      labs(title = "Number of Repeats Needed for Desired Validity Coefficient",
           x = "Intraclass Correlation Coefficient (ICC)",
           y = "Number of Repeats (m)",
           subtitle = paste("Desired Validity Coefficient (rhoXW) =", rhoXW_value)) +
      geom_vline(xintercept = input$icc, linetype = "dashed", color = "red") +
      geom_hline(yintercept = calculateRepeats(input$icc, rhoXW_value)$ceiling_m, linetype = "dashed", color =
"red") +
      annotate("text", x = input$icc, y = calculateRepeats(input$icc, rhoXW_value)$ceiling_m,
               label = paste("m =", calculateRepeats(input$icc, rhoXW_value)$ceiling_m), color = "red", hjust = -0.1,
vjust = -1, size = 5) +
      theme(plot.title = element_text(hjust = 0.5, size = 20),
            axis.title = element_text(size = 16),
            axis.text = element_text(size = 16),
            axis.line = element_line(size = 1, colour = "black"),
            plot.caption = element_text(size = 14, hjust = 0),
            plot.subtitle = element_text(size = 16),
            legend.text = element_text(size = 14),
            legend.title = element_text(size = 14))

    p
  })
}

# Run the application
shinyApp(ui = ui, server = server)
```

8.3 User's Guide for Simple Linear Regression (SLR) n (Number of subjects) Sample Size Calculator with Measurement Error

8.3.1 Introduction

The Simple Linear Regression (SLR) Sample Size Calculator with Measurement Error (https://scipinion.shinyapps.io/LinearRegressionN/) is a Shiny application designed to help users interactively explore the relationship between sample size and the minimum detectable effect (MDE) in a linear regression model, considering classical additive measurement errors in the exposure estimates. The application allows users to understand how these errors influence the required sample size to achieve desired statistical properties.

8.3.2 Overview

This Shiny application calculates the sample size needed for a simple linear regression (SLR) analysis, accounting for classical additive measurement error. The application provides two primary sample size calculations: one without measurement error (n_X) and one with measurement error (n_Z). Additionally, it calculates the coefficient of determination (r-squared).

8.3.2.1 User Interface

The UI consists of two main sections:

- Input Panel (Left Sidebar)
- Output Panel (Main Area)

8.3.2.2 Input Panel

- Variance of Y (vy): Numeric input for the variance of the dependent variable Y. Default is 1.
- Within-Subject Variance (vw): Numeric input for the within-subject variance Vw. Default is 1.
- Number of Measurements per Subject (m): Numeric input for the number of measurements per subject. Default is 1.
- Input Mode (mode): Radio buttons to select input mode. Options are "Use ICC Slider" and "Enter Vb and Vw".
- Significance Level (alpha): Slider to set the significance level (alpha). Default is 0.05.
- Power (power): Slider to set the desired power (1-beta). Default is 0.90.
- Minimum Detectable Effect Size (mdeInput): Numeric input for entering a specific MDE value. Default is 0.30.
- Calculate Button (calculate): Button to perform the sample size calculation.

Depending on the selected input mode, the UI will dynamically show:

- Intraclass Correlation Coefficient (ICC) Slider (icc): For "Use ICC Slider" mode. Default is 0.50.
- Between-Subject Variance (vb): For "Enter Vb and Vw" mode. Default is 1.

8.3.2.3 Output Panel

- Sample Size vs. MDE Plot (tradeoffPlot): Plot showing the relationship between sample size and MDE.
- Result Text (resultText): Text output summarizing the results of the calculations.
- n_X Output (nXOutput): Text output for the calculated n_X (sample size without measurement error).
- n_Z Output (nZOutput): Text output for the calculated n_Z (sample size with measurement error).
- r-squared Output (rSquaredOutput): Text output for the calculated r-squared.

8.3.2.4 How to Use

1. Enter Variance of Y and Within-Subject Variance: Specify the values for vy and vw.
2. Specify Number of Measurements per Subject: Enter the number of measurements m.
3. Select Input Mode:
    - Use ICC Slider: Adjust the ICC using the slider.
    - Enter Vb and Vw: Directly input the between-subject variance vb.
4. Set Significance Level and Power: Adjust the alpha and power sliders.
5. Enter MDE: Input a specific MDE value or use the default.
6. Calculate: Click the "Calculate" button to perform the sample size calculation.
7. Review Results: The results, including the plot and text summaries, will be displayed in the main panel.

8.3.2.5 Calculation Details

- ICC Calculation: If "Enter Vb and Vw" mode is selected, ICC is calculated as: ICC = Vb / (Vb + Vw)
- Standardized Effect Size (d): Calculated as: d = MDE * Sx / Sres where Sx is the standard deviation of the between-subject variance, and Sres is the standard deviation of the residual variance.
- Sample Size without Measurement Error (n_X): n_X = ((z_beta + z_alpha/2) / d)^2
- Sample Size with Measurement Error (n_Z): n_Z = n_X / (Vb / (Vb + Vw / m))
- Coefficient of Determination (r-squared): r-squared = 1 - (Vres / Vy)

8.3.2.6 Derivations and Justifications

1. **ICC Calculation**: The Intraclass Correlation Coefficient (ICC) is calculated as: ICC = Vb / (Vb + Vw) This formula derives from the definition of ICC as the ratio of between-subject variance (Vb) to the total variance (sum of between-subject and within-subject variance).
2. **Standardized Effect Size (d)**: The standardized effect size (d) is calculated as: d = MDE * Sx / Sres Here, Sx is the standard deviation of the between-subject variance, and Sres is the standard deviation of the residual variance. This formula standardizes the minimum detectable effect size (MDE) by the variability in the residuals, making it a unitless measure of effect size.
3. **Sample Size without Measurement Error (n_X)**: The sample size without measurement error (n_X) is calculated using the formula: n_X = ((z_beta + z_alpha/2) / d)^2 This formula is derived from the requirements for achieving the specified power (1-

beta) and significance level (alpha) in a hypothesis test. The z-values correspond to the critical values of the standard normal distribution for the specified alpha and beta.

4. **Sample Size with Measurement Error (n_Z)**: The sample size with measurement error (n_Z) is adjusted from n_X by accounting for the measurement error. It is calculated as: n_Z = n_X / (Vb / (Vb + Vw / m)) This adjustment reflects the increased sample size needed to achieve the same power when measurement error is present. The denominator represents the proportion of variance due to true between-subject differences when measurement error is considered.
5. **Coefficient of Determination (r-squared)**: The coefficient of determination (r-squared) is calculated as: r-squared = 1 - (Vres / Vy) This formula represents the proportion of the variance in the dependent variable Y that is predictable from the independent variables, adjusting for the residual variance (Vres).

```
# Source code for simple linear regression (SLR) sample size calculator with measurement
error, 4-30-2024


# Setup block to install and load required packages
packages <- c("shiny", "ggplot2", "pwr")
installed_packages <- rownames(installed.packages())

for (package in packages) {
  if (!package %in% installed_packages) {
    install.packages(package)
  }
  library(package, character.only = TRUE)
}

# Define UI
ui <- fluidPage(
  titlePanel("Sample Size and MDE Tradeoff Calculation for Linear Regression with Classical
Additive Measurement Error"),
  sidebarLayout(
    sidebarPanel(
      numericInput("vy", "Variance of Y:", value = 1, min = 0.0001),
      numericInput("vw", "Within-Subject Variance (Vw):", value = 1, min = 0.0001),
      numericInput("m", "Number of measurements per subject (m):", value = 1, min = 1, step =
1),
      radioButtons("mode", "Select Input Mode:",
                   choices = list("Use ICC Slider" = "icc_slider", "Enter Vb and Vw" =
"var_components"),
                   selected = "icc_slider"),
      uiOutput("varInputUI"),
      sliderInput("alpha", "Significance Level (alpha):", min = 0.01, max = 0.1, value = 0.05),
      sliderInput("power", "Power (1-beta):", min = 0.8, max = 0.99, value = 0.9),
      actionButton("calculate", "Calculate", class = "btn btn-success")
    ),
    mainPanel(
      plotOutput("tradeoffPlot", height = "400px"),
      textOutput("resultText"),
      numericInput("mdeInput", "Enter a Specific MDE Value:", value = 0.3, min = 0.01, step =
0.01),
      verbatimTextOutput("nXOutput"),
      verbatimTextOutput("nZOutput"),
      verbatimTextOutput("rSquaredOutput")
    )
  )
)

# Define server logic
server <- function(input, output, session) {
  # Dynamic UI for variance input mode
  output$varInputUI <- renderUI({
    if (input$mode == "icc_slider") {
```

```
      sliderInput("icc", "Intraclass Correlation Coefficient (ICC):", min = 0, max = 1, value = 0.5,
step = 0.01)
    } else {
      numericInput("vb", "Between-Subject Variance (Vb):", value = 1, min = 0.0001)
    }
  })

  # Calculate ICC based on mode
  reactive_icc <- reactive({
    if (input$mode == "icc_slider") {
      input$icc
    } else {
      input$vb / (input$vb + input$vw)  # Convert Vb to ICC for direct entry mode
    }
  })

  # Use reactive_icc for calculations
  reactive_calculate <- eventReactive(input$calculate, {
    z_alpha_2 <- qnorm(1 - input$alpha / 2)
    z_beta <- qnorm(input$power)
    mde <- input$mdeInput
    Vy <- input$vy
    Vw <- input$vw
    m <- input$m
    Vb <- input$vw * reactive_icc() / (1 - reactive_icc())  # Compute Vb using ICC

    Vres <- Vy - mde^2 * Vb
    Sx <- sqrt(Vb)
    Sres <- sqrt(Vres)

    # Calculate standardized effect size
    d <- mde * Sx / Sres

    # Calculate n_X (sample size without measurement error)
    n_X <- ((z_beta + z_alpha_2) / d)^2

    # Calculate n_Z (sample size with measurement error)
    n_Z <- n_X / (Vb / (Vb + Vw / m))

    # Calculate r^2
    r_squared <- 1 - (Vres / Vy)

    list(n_X = n_X, n_Z = n_Z, r_squared = r_squared, mde = mde)
  })

  # Output the calculated n_X, n_Z, and r^2
  output$nXOutput <- renderText({
    calc <- reactive_calculate()
    sprintf("Calculated n_X (without measurement error) for MDE = %.2f: %.0f", calc$mde,
calc$n_X)
  })
```

```
  output$nZOutput <- renderText({
    calc <- reactive_calculate()
    sprintf("Calculated n_Z (with measurement error) for MDE = %.2f: %.0f", calc$mde, calc$n_Z)
  })

  output$rSquaredOutput <- renderText({
    calc <- reactive_calculate()
    sprintf("Calculated r^2 for MDE = %.2f: %.4f", calc$mde, calc$r_squared)
  })

  # Render the plot and result text
  observeEvent(input$calculate, {
    mde_range <- seq(0.1, 1, by = 0.01)
    Vb <- input$vw * reactive_icc() / (1 - reactive_icc())  # Use calculated ICC

    sample_sizes <- sapply(mde_range, function(mde) {
      Vy <- input$vy
      Vw <- input$vw
      m <- input$m
      Vres <- Vy - mde^2 * Vb
      Sx <- sqrt(Vb)
      Sres <- sqrt(Vres)
      d <- mde * Sx / Sres
      n_X <- ((qnorm(1 - input$alpha / 2) + qnorm(input$power)) / d)^2
      n_Z <- n_X / (Vb / (Vb + Vw / m))
      r_squared <- 1 - (Vres / Vy)
      n_Z
    })

    plot_data <- data.frame(MDE = mde_range, SampleSize = sample_sizes)

    output$tradeoffPlot <- renderPlot({
      ggplot(plot_data, aes(x = MDE, y = SampleSize)) +
        geom_line(color = "blue") +
        geom_vline(xintercept = input$mdeInput, linetype = "dashed", color = "red") +
        geom_hline(yintercept = reactive_calculate()$n_Z, linetype = "dashed", color = "red") +
        geom_point(aes(x = input$mdeInput, y = reactive_calculate()$n_Z), color = "red", size = 3)
+
        labs(title = "Sample Size vs. Minimum Detectable Effect Size",
             x = "Minimum Detectable Effect Size (MDE)",
             y = "Required Sample Size (n_Z)") +
        theme_minimal() +
        theme(plot.title = element_text(hjust = 0.5, size = 20),
              axis.title = element_text(size = 18),
              axis.text = element_text(size = 16),
              axis.line = element_line(color = "black"),
              panel.grid.major = element_line(color = "grey90"),
              panel.grid.minor = element_line(color = "grey98"),
              panel.background = element_blank())
    })
```

```
    output$resultText <- renderText({
      calc <- reactive_calculate()
      sprintf("With inputs Vy=%.2f, Vw=%.2f, m=%d, alpha=%.3f, and power=%.2f, the calculated
sample sizes range from %.0f to %.0f for MDE values from %.2f to %.2f. Calculated r^2 for MDE
= %.2f: %.4f",
              input$vy, input$vw, input$m, input$alpha, input$power,
              min(sample_sizes), max(sample_sizes), min(mde_range), max(mde_range),
calc$mde, calc$r_squared)
    })
  })
}

# Run the app
shinyApp(ui = ui, server = server)
```

8.4 User's Guide for Simple Linear Regression (SLR) m (Number of Measurements per Subject) Calculator with Measurement Error

8.4.1 Introduction

The Simple Linear Regression (SLR) Sample Size Calculator with Measurement Error (https://scipinion.shinyapps.io/LinearRegressionM/) is a Shiny application designed to help users interactively explore the relationship between the number of repeated measurements per subject (m) and the minimum detectable effect (MDE) in a linear regression model, considering classical additive measurement errors in the exposure estimates. The application allows users to understand how these errors influence the number of measurements needed to achieve desired statistical properties.

8.4.2 Using the Application

8.4.2.1 Interface Overview

The SLR Sample Size Calculator application interface consists of a sidebar panel with various input controls and a main panel displaying the plots containing the outputs.

8.4.2.2 Input Controls

- **Variance of Y**: Enter the variance of the response variable (Y).
- **Within-Subject Variance (Vw)**: Enter the within-subject variance.
- **Sample Size (n)**: Enter the sample size.
- **Select Input Mode**: Choose between using an ICC slider or entering between-subject and within-subject variance directly.
- **Intraclass Correlation Coefficient (ICC)**: Adjust the ICC using the slider if the ICC slider mode is selected.
- **Between-Subject Variance (Vb)**: Enter the between-subject variance if the variance components mode is selected.
- **Significance Level (alpha)**: Adjust the significance level for the test.
- **Power (1-beta)**: Set the desired statistical power.
- **Calculate Button**: Press to calculate the required number of measurements and other statistics.
- **Enter a Specific m Value**: Enter a specific number of repeated measurements per subject to see the corresponding MDE.

8.4.2.3 Output Display

- **MDE vs. Number of Measurements Plot**: Displays the relationship between the minimum detectable effect size and the number of repeated measurements per subject.
- **Result Text**: Provides a summary of the calculated results, including the range of MDE values for different numbers of measurements.
- **Calculated MDE for Specific m Value**: Displays the MDE for a specific number of measurements per subject.

8.4.3 Steps to Use the SLR Sample Size Calculator Application

1. **Adjust Input Parameters**: Use the input controls on the sidebar to set the desired values for variance of Y, within-subject variance, sample size, input mode, significance level, and power.
2. **Calculate**: Press the "Calculate" button to compute the required number of measurements and other statistics.
3. **View Results**: The plot and result text will update automatically based on the selected inputs. You can adjust the input parameters and recalculate as needed.
4. **Enter Specific m Value**: Enter a specific number of repeated measurements per subject in the provided input box to see the corresponding MDE.

8.4.4 Example

To explore the impact of different numbers of measurements per subject on the minimum detectable effect size with a variance of Y of 2, within-subject variance of 1.5, sample size of 100, a significance level of 0.05, and a power of 0.9:

1. Set the **Variance of Y** to 2.
2. Set the **Within-Subject Variance** to 1.5.
3. Set the **Sample Size** to 100.
4. Set the **Significance Level** to 0.05.
5. Set the **Power** to 0.9.
6. Choose the input mode (e.g., ICC slider) and adjust accordingly.
7. Press the **Calculate** button to see the effect on the MDE.
8. Optionally, enter a specific number of measurements per subject (m) to view the corresponding MDE.

The SLR Sample Size Calculator should produce output that helps you understand the relationship between the number of measurements per subject, MDE, and the influence of measurement errors on the statistical power of your study.

```
# Source code for m calculator, m = number of measurements per subject

# Setup block to install and load required packages
packages <- c("shiny", "ggplot2", "pwr")
installed_packages <- rownames(installed.packages())

for (package in packages) {
  if (!package %in% installed_packages) {
    install.packages(package)
  }
  library(package, character.only = TRUE)
}

# Define UI
ui <- fluidPage(
  titlePanel("Sample Size and MDE Tradeoff Calculation for Linear Regression with Classical
Additive Measurement Error"),
  sidebarLayout(
    sidebarPanel(
      numericInput("vy", "Variance of Y:", value = 2, min = 0.0001),
      numericInput("vw", "Within-Subject Variance (Vw):", value = 1.5, min = 0.0001),
      numericInput("n", "Sample Size (n):", value = 100, min = 1, step = 1),
      radioButtons("mode", "Select Input Mode:",
                   choices = list("Use ICC Slider" = "icc_slider", "Enter Vb and Vw" =
"var_components"),
                   selected = "icc_slider"),
      uiOutput("varInputUI"),
      sliderInput("alpha", "Significance Level (alpha):", min = 0.01, max = 0.1, value = 0.05),
      sliderInput("power", "Power (1-beta):", min = 0.8, max = 0.99, value = 0.9),
      actionButton("calculate", "Calculate", class = "btn btn-success"),
      numericInput("mInput", "Enter a Specific m Value:", value = 1, min = 1, max = 20, step = 1)
    ),
    mainPanel(
      plotOutput("tradeoffPlot", height = "400px"),
      textOutput("resultText"),
      verbatimTextOutput("mdeOutput")
    )
  )
)

# Define server logic
server <- function(input, output, session) {
  # Dynamic UI for variance input mode
  output$varInputUI <- renderUI({
    if (input$mode == "icc_slider") {
      sliderInput("icc", "Intraclass Correlation Coefficient (ICC):", min = 0, max = 1, value = 0.5,
step = 0.01)
    } else {
      numericInput("vb", "Between-Subject Variance (Vb):", value = 1, min = 0.0001)
    }
```

```
  })

  # Calculate ICC based on mode
  reactive_icc <- reactive({
    if (input$mode == "icc_slider") {
      input$icc
    } else {
      input$vb / (input$vb + input$vw)  # Convert Vb to ICC for direct entry mode
    }
  })

  # Use reactive_icc for calculations
  reactive_calculate <- eventReactive(input$calculate, {
    z_alpha_2 <- qnorm(1 - input$alpha / 2)
    z_beta <- qnorm(input$power)
    Vy <- input$vy
    Vw <- input$vw
    n_Z <- input$n
    ICC <- reactive_icc()
    Vb <- Vw * ICC / (1 - ICC)  # Compute Vb using ICC

    mde_range <- seq(1, 20, by = 1)  # Fixed range for m

    mde_values <- sapply(mde_range, function(m) {
      sqrt((z_beta + z_alpha_2)^2 * Vy / (n_Z * (Vb / (Vb + Vw / m)) + (z_beta + z_alpha_2)^2 *
Vb))
    })

    list(mde_range = mde_range, mde_values = mde_values, Vb = Vb)
  })

  # Calculate MDE for a specific m value
  reactive_specific_mde <- reactive({
    calc <- reactive_calculate()
    m <- input$mInput
    Vb <- calc$Vb
    z_alpha_2 <- qnorm(1 - input$alpha / 2)
    z_beta <- qnorm(input$power)
    Vy <- input$vy
    Vw <- input$vw
    n_Z <- input$n

    specific_mde <- sqrt((z_beta + z_alpha_2)^2 * Vy / (n_Z * (Vb / (Vb + Vw / m)) + (z_beta +
z_alpha_2)^2 * Vb))
    specific_mde
  })

  # Output the calculated MDE for a specific m value
  output$mdeOutput <- renderText({
    specific_mde <- reactive_specific_mde()
    sprintf("Calculated MDE for m = %d: %.4f", input$mInput, specific_mde)
```

```
  })

  # Render the plot and result text
  observeEvent(input$calculate, {
    calc <- reactive_calculate()
    plot_data <- data.frame(M = calc$mde_range, MDE = calc$mde_values)
    specific_mde <- reactive_specific_mde()

    output$tradeoffPlot <- renderPlot({
      ggplot(plot_data, aes(x = M, y = MDE)) +
        geom_line(color = "blue") +
        geom_vline(xintercept = input$mInput, linetype = "dashed", color = "red") +
        geom_hline(yintercept = specific_mde, linetype = "dashed", color = "red") +
        geom_point(aes(x = input$mInput, y = specific_mde), color = "red", size = 3) +
        labs(title = "MDE vs. Number of Measurements per Subject (m)",
             x = "Number of Measurements per Subject (m)",
             y = "Minimum Detectable Effect Size (MDE)") +
        theme_minimal() +
        theme(plot.title = element_text(hjust = 0.5, size = 20),
              axis.title = element_text(size = 18),
              axis.text = element_text(size = 16),
              axis.line = element_line(color = "black"),
              panel.grid.major = element_line(color = "grey90"),
              panel.grid.minor = element_line(color = "grey98"),
              panel.background = element_blank())
    })

    output$resultText <- renderText({
      sprintf("With inputs Vy=%.2f, Vw=%.2f, n=%d, alpha=%.3f, and power=%.2f, the calculated
MDE values range from %.2f to %.2f for m values from 1 to 20.",
              input$vy, input$vw, input$n, input$alpha, input$power, min(calc$mde_values),
max(calc$mde_values))
    })
  })
}

# Run the app
shinyApp(ui = ui, server = server)
```

8.5 User's Guide for Logistic Regression Sensitivity Analysis Explorer

8.5.1 Introduction

The Sensitivity Analysis Explorer (https://scipinion.shinyapps.io/SensitivityAnalysisExplorer/) is a Shiny application designed to help users interactively examine the sensitivity of power and bias of simple logistic regression model to errors in exposure estimates (covariate). It uses simulated data to provide insights into how different factors influence the outcomes of logistic regression with classical additive measurement errors in the exposure estimates.

8.5.2 Running the Sensitivity Analysis Explorer Application

Steps

1. Open RStudio: If you are using RStudio, open it.
2. Set Working Directory: Set your working directory to the location where app.R and simdata.csv are saved. You can do this in RStudio by selecting Session > Set Working Directory > Choose Directory... and then navigating to the appropriate folder.
3. Run the Application: In the R console, run the following command to start the Shiny app:

```
shiny::runApp("app.R")
```

This command will launch the Sensitivity Analysis Explorer in your default web browser.

8.5.3 Using the Application

8.5.3.1 Interface Overview

The Sensitivity Analysis Explorer application interface consists of a sidebar panel with various input controls and a main panel displaying the plots containing the outputs.

8.5.3.2 Input Controls

- **Odds Ratio:** Change the presumed true odds ratio using the slider to see its effect on the power and bias analyses.
- **Number of Repeated Measurements:** Use the slider to set the number of repeated measurements per person (subject).
- **Between-Person Variance:** Adjust the between-person variance using the slider.
- **Within-Person Variance:** Adjust the within-person variance using the slider.
- **Baseline Probability:** Use the slider to set the baseline probability for the response variable. The response variable in a logistic regression model is assumed to be binary, e.g., 1 = adverse effect occurs, 0 = it does not occur. The baseline probability is the probability that it occurs when exposure is equal to 0 (assuming non-negative exposure values).
- **Sample Size:** Adjust the desired sample size (number of individuals selected for study) using the slider.

- **Show Confidence Intervals:** Check this box to display confidence intervals around the power and bias curves.
- **Type 1 error (p-**value) is fixed at 5% (two-sided).

8.5.3.3 Output Display

- **Power Analysis Plot:** Shows the power curve based on the selected input parameters. The power curve represents the probability of correctly rejecting the null hypothesis of no effect (i.e., detecting a true effect) with a give level of type 1 error (p-value) as a function of sample size. A higher power value indicates a greater likelihood of detecting an actual effect when it exists.
- **Bias Analysis Plot:** Shows the bias curve based on the selected input parameters. The bias curve indicates the average percentage deviation of the estimated effect size from the true effect size. Lower bias values indicate more accurate (less biased) estimates.
- **Table Output:** Displays a table of the filtered data based on the selected input parameters. This table includes detailed simulation results such as power, bias, intra-class correlation (ICC), false positive rate (FPR), and false negative rate (FNR) for each combination of parameters. FPR and FNR are calculated under prior assumption of probability of 50% of the null hypothesis being true.

8.5.3.4 Steps to Use the Sensitivity Analysis Explorer Application

1. **Adjust Input Parameters:** Use the sliders on the sidebar to adjust the input parameters to your desired values.
2. **View Results:** The power and bias analysis plots and the table output will update automatically based on the selected inputs. If the "Show Confidence Intervals" checkbox is checked, confidence intervals will be displayed on the plots.

8.5.3.5 Example

To explore the impact of different sample sizes on power and bias with an odds ratio of 1.1, between-person variance of 6, within-person variance of 3, baseline probability of 0.1, and number of repeated measurements set to 2:

1. Set the Odds Ratio slider to 1.1.
2. Set the Between-Person Variance slider to 6.
3. Set the Within-Person Variance slider to 3.
4. Set the Baseline Probability slider to 0.1.
5. Set the Number of Repeated Measurements slider to 2.
6. Adjust the Sample Size slider to see the effect on the power and bias curves.
7. Optionally, check the "Show Confidence Intervals" box to view the confidence bands.

The Sensitivity Analysis Explorer should produce output such as the following.

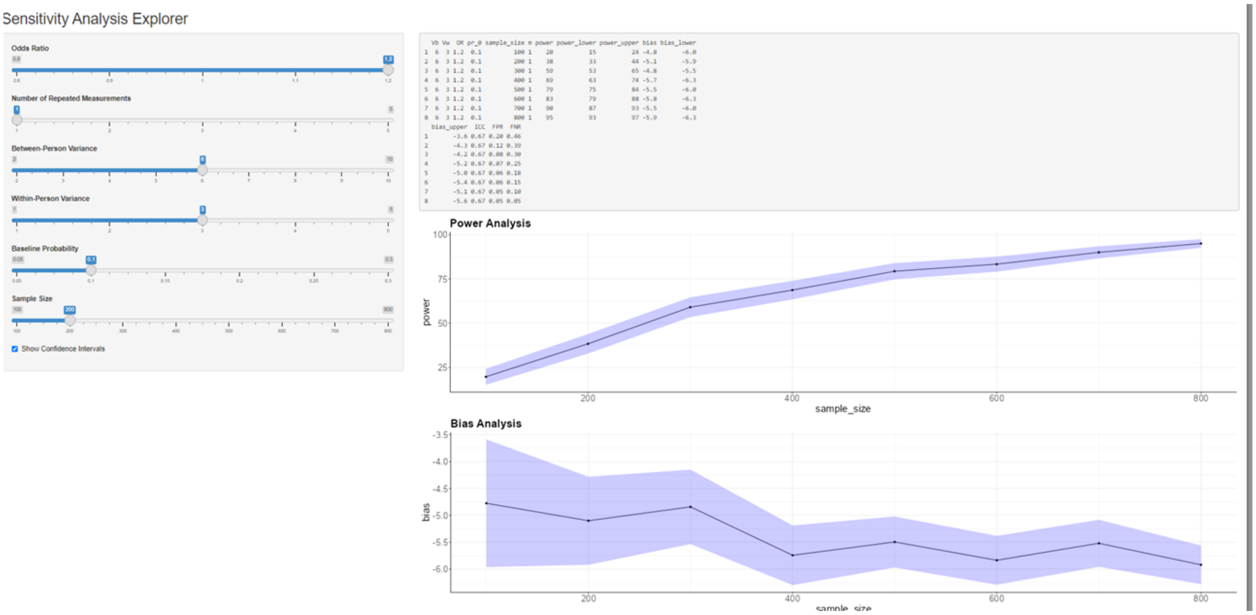
Sensitivity Analysis Explorer
Odds Ratio
Number of Repeated Measurements
Between-Person Variance
Within-Person Variance
Baseline Probability
Sample Size
Show Confidence Intervals
Vb Vw OR pr_0 sample_size m power power_lower power_upper bias bias_lower
1 6 3 1.2 0.1 100 1 20 15 24 -4.8 -6.0
2 6 3 1.2 0.1 200 1 38 33 44 -5.1 -5.9
3 6 3 1.2 0.1 300 1 59 53 65 -4.8 -5.5
4 6 3 1.2 0.1 400 1 69 63 74 -5.7 -6.3
5 6 3 1.2 0.1 500 1 79 75 84 -5.5 -6.0
6 6 3 1.2 0.1 600 1 83 79 88 -5.8 -6.3
7 6 3 1.2 0.1 700 1 90 87 93 -5.5 -6.0
8 6 3 1.2 0.1 800 1 95 93 97 -5.9 -6.3
bias_upper ICC FPR FNR
1 -3.6 0.67 0.20 0.46
2 -4.3 0.67 0.12 0.39
3 -4.2 0.67 0.08 0.30
4 -5.2 0.67 0.07 0.25
5 -5.0 0.67 0.06 0.18
6 -5.4 0.67 0.06 0.15
7 -5.1 0.67 0.05 0.10
8 -5.6 0.67 0.05 0.05
Power Analysis
power
sample_size
Bias Analysis
bias

```
#  Source code for Sensitivity Analysis Explorer code( L-calculator), 6-21-24
library(shiny)
library(ggplot2)
library(dplyr)

# Load the simulation results
results_df <- read.csv("simdata.csv")

# Define UI
ui <- fluidPage(
  titlePanel("Sensitivity Analysis Explorer"),
  sidebarLayout(
    sidebarPanel(
      sliderInput("OR", "Odds Ratio", min = 0.8, max = 1.2, value = 1.0, step = 0.1),
      sliderInput("m", "Number of Repeated Measurements", min = 1, max = 5, value = 1, step =
1),
      sliderInput("Vb", "Between-Person Variance", min = 2, max = 10, value = 6, step = 2),
      sliderInput("Vw", "Within-Person Variance", min = 1, max = 5, value = 3, step = 1),
      sliderInput("pr_0", "Baseline Probability", min = 0.05, max = 0.3, value = 0.1, step = 0.05),
      sliderInput("sample_size", "Sample Size", min = 100, max = 800, value = 200, step = 100),
      checkboxInput("show_ci", "Show Confidence Intervals", value = FALSE)
    ),
    mainPanel(
      verbatimTextOutput("debug_output"),
      plotOutput("plot_power"),
      plotOutput("plot_bias")
    )
  ),
  tags$style(HTML("
    .irs-grid-text, .irs-min, .irs-max, .irs-from, .irs-to, .irs-single {
      font-size: 18px !important;
    }
    .irs-slider {
      width: 20px;
      height: 20px;
    }
    .checkbox label {
      font-size: 18px;
    }
  "))
)

# Define server
server <- function(input, output) {
  filtered_data <- reactive({
    results_df %>%
      filter(OR == input$OR,
             m == input$m,
             Vb == input$Vb,
             Vw == input$Vw,
             pr_0 == input$pr_0)
```

```
})

output$debug_output <- renderPrint({
  data <- filtered_data()
  data <- data %>%
    mutate(across(everything(), round, 2))
  print(data, digits = 2)
})

output$plot_power <- renderPlot({
  data <- filtered_data()
  if (nrow(data) > 0) {
    p <- ggplot(data, aes(x = sample_size, y = power, group = 1)) +
      geom_line(color = "black") +
      geom_point(color = "black") +
      ggtitle("Power Analysis") +
      theme_minimal() +
      theme(
        axis.title = element_text(size = 20),
        axis.text = element_text(size = 18),
        plot.title = element_text(size = 22, face = "bold"),
        axis.line = element_line(color = "black"),
        axis.ticks = element_line(color = "black")
      )
    if (input$show_ci) {
      p <- p +
        geom_ribbon(aes(ymin = power_lower, ymax = power_upper), alpha = 0.2, fill = "blue")
    }
    print(p)
  } else {
    ggplot() +
      ggtitle("No data available for selected parameters") +
      theme_minimal() +
      theme(
        plot.title = element_text(size = 22, face = "bold")
      )
  }
})

output$plot_bias <- renderPlot({
  data <- filtered_data()
  if (nrow(data) > 0) {
    p <- ggplot(data, aes(x = sample_size, y = bias, group = 1)) +
      geom_line(color = "black") +
      geom_point(color = "black") +
      ggtitle("Bias Analysis") +
      theme_minimal() +
      theme(
        axis.title = element_text(size = 20),
        axis.text = element_text(size = 18),
        plot.title = element_text(size = 22, face = "bold"),
```

```
        axis.line = element_line(color = "black"),
        axis.ticks = element_line(color = "black")
      )
    if (input$show_ci) {
      p <- p +
        geom_ribbon(aes(ymin = bias_lower, ymax = bias_upper), alpha = 0.2, fill = "blue")
    }
    print(p)
  } else {
    ggplot() +
      ggtitle("No data available for selected parameters") +
      theme_minimal() +
      theme(
        plot.title = element_text(size = 22, face = "bold")
      )
  }
 })
}

# Run the application
shinyApp(ui = ui, server = server)
```
